# Ancient origin of Jingchuvirales derived glycoproteins integrated in arthropod genomes

**DOI:** 10.1590/1678-4685-GMB-2022-0218

**Published:** 2023-04-07

**Authors:** Filipe Zimmer Dezordi, Gutembergmann Batista Coutinho, Yago José Mariz Dias, Gabriel Luz Wallau

**Affiliations:** ¹ Fundação Oswaldo Cruz (FIOCRUZ), Instituto Aggeu Magalhães (IAM), Departamento de Entomologia, Recife, PE, Brazil.; ² Fundação Oswaldo Cruz (FIOCRUZ), Instituto Aggeu Magalhães (IAM), Núcleo de Bioinformática, Recife, PE, Brazil.; 3 WHO Collaborating Center for Arbovirus and Hemorrhagic Fever Reference and Research, Bernhard Nocht Institute for Tropical Medicine, Department of Arbovirology, Hamburg, Germany.; 4 Universidade Federal de Pernambuco, Centro de Biociências, Recife, PE, Brazil.

**Keywords:** Jingchuvirales, endogenous viruses, genome, insects

## Abstract

Endogenous virus elements (EVEs) are viral-derived sequences integrated into their host genomes. EVEs of the *Jingchuvirales* order were detected in a wide range of insect genomes covering several distantly related families. Moreover, *Jingchuvirales*-derived glycoproteins were recently associated by our group with the origin of a putative new retrovirus based on a glycoprotein captured by a mosquito retrotransposon. But, except for mosquitoes, there is a lack of a more detailed understanding of the endogenization mechanism, timing, and frequency per *Jingchuvirales* viral lineages. Here we screened *Jingchuvirales* glycoprotein-derived EVEs (Jg-EVEs) in eukaryotic genomes. We found six distinct endogenization events of Jg-EVEs, that belong to two out of five known *Jingchuvirales* families (*Chuviridae* and *Natareviridae*). For seven arthropod families bearing Jg-EVEs there is no register of *bona fide* circulating chuvirus infection. Hence, our results show that *Jingchuvirales* viruses infected or still infect these host families. Although we found abundant evidence of LTR-Gypsy retrotransposons fragments associated with the glycoprotein in Hymenoptera and other insect orders, our results show that the widespread distribution of *Jingchuvirales* glycoproteins in extant Arhtropods is a result of multiple ancient endogenization events and that these virus fossils are being vertically inherited in Arthropods genomes for millions of years.

## Introduction

Viruses are the most abundant and diverse nucleic acid-based replicating units on Earth (Koonin and Krupovic, 2018). These parasitic replicating units rely on infection and exploitation of cellular organism’s molecular machinery for their own replication. Because of this intimate and critical relationship with host cells, viruses and hosts undergo several interaction steps, even at the genome level (Weiss, 2017; Coffin et al., 2021). Retroviruses are known to integrate their genome into their host genome giving origin to Endogenous Retrovirus (ERVs). When integration takes place in germline cells, ERVs can be inherited by the next host generation (Feschotte and Gilbert, 2012; Johnson, 2019). Interestingly, non-retroviral viruses can also leave traces of past infection in their host genomes, and evidence of Non-retroviral Integrated RNA Virus Sequences (NIRVs), more broadly known as Endogenous Viral Elements (EVEs), can be found in a wide range of multicellular eukaryotic species (Katzourakis and Gifford, 2010). Increasing evidence shows that EVEs insertions impact on the host organism range from deleterious, neutral or positive fitness advantage (Ito et al., 2013; Armezzani et al., 2014; Ter Horst et al., 2019; Suziki *et al*., 2020) and that the presence of EVEs in population or species depends on their host fitness impact and endogenization rate (Feschotte and Gilbert, 2012; Johnson, 2019).

The integration mechanism of non-retroviral sequences lacking retro transcriptases and integrases is still an open and intriguing phenomenon. It is notorious that the major part of EVEs identified in insects are derived from non-retroviruses (Gilbert and Belliardo, 2022). One of the most likely hypotheses is that integration is mediated by reverse transcriptase and integrases encoded by endogenous retrotransposons (Katzourakis and Gifford, 2010; Holmes, 2011). Retrotransposons are abundant and active in insect genomes and may provide proteins *in trans* for viral cDNA synthesis and integration (Tassetto et al., 2019). Although integration may also occur through non-homologous recombination mediated by the double-strand break repair mechanism of the host (Katzourakis and Gifford, 2010). Moreover, there are clear discrepancies regarding EVEs viral families diversity in insects, that is, the most widespread and abundant EVEs derive from two (*Rhabdoviridae* and *Chuviridae*) out of 49 known viral families (Blair et al., 2020, NCBI Virus). This raises at least two interesting and related questions: Are those viral families infecting insects more frequently than other viral taxa, thus increasing the chance of leaving more EVEs in their host genomes? Do viral genomes from these families interact more frequently with endogenous retrotransposon proteins increasing their endogenization rate relative to other viral families? The availability of several insect genomes and detailed characterization of EVEs may provide indirect evidence to answer these questions. 

The *Jingchuvirales* order was first characterized in 2015 based on several complete genomes grouping into a large, well-supported and distinct monophyletic group of viruses found majoritarially in hosts of the orders Araneae, Neuroptera, Decapoda, Diptera and Ixodida (Li et al., 2015). These viruses were initially grouped into only one family (*Chuviridae*) with a negative-sense single-stranded RNA (ssRNA (-)) genome with distinct genomic structure conformations such as unsegmented, segmented, linear or circular genomes (Li *et al*., 2015). Up to now, no viral isolation has been performed for viruses from that family and its description is restricted to viral genome sequences. In 2018, the International Committee on Taxonomy of Viruses (ICTV) created the *Jingchuvirales* order represented by the *Chuviridae* family only (Wolf et al., 2018) and more recently this family was split into 5 families and 19 genera based on RNA-dependent RNA polymerase (RdRp) similarity thresholds (Di Paola et al., 2021). Interestingly, several homologous glycoprotein sequences of Chuviruses - the proteins that form the viral envelope - were found integrated into different host genomes, including mosquitoes (Li *et al*., 2015; Whitfield et al., 2017; Russo et al., 2019; Palatini et al., 2020; Dezordi et al., 2020), ticks (Li *et al*., 2015; Russo *et al*., 2019), flies (Li *et al*., 2015) and ants (Flynn and Moreau, 2019). Previous studies identified a higher number of endogenous glycoproteins of the *Chuviridae* family in different insect genomes when compared with endogenous nucleoproteins and polymerases (Whitfield *et al*., 2017; Russo *et al*., 2019). Our group recently showed that, in mosquitoes, such discrepancy occurred due to *Chuviridae* glycoproteins captured by endogenous retrotransposons followed by intragenomic replication and hence amplification of the glycoprotein sequences (Dezordi *et al*., 2020).

The new *Jingchuvirales* order and following family and genus level classification, and the higher proportion of *Chuviridae* glycoproteins endogenized in insect genomes led us to investigate a number of related questions in this study: Are there differences of Jg-EVE endogenization origin from the five *Jingchuvirales* families? Are there specific associations of viral taxa (family/genus), host taxa and endogenous retrotransposons that explain the Jg-EVEs emergence and maintenance through evolutionary time? What was the timing of endogenization events in the evolutionary history of arthropods? Here, we performed an extensive literature review to catalog all complete genomes of *Jingchuvirales* order available, screened *Jingchuvirales* glycoprotein in eukaryotic genomes and reconstructed the phylogenetic history of complete genomes and endogenized glycoproteins. We detected that two out of five viral families of the order *Jingchuvirales* were involved in six ancient endogenization events and that all extant and widespread Jg-EVEs found in this study are derived from these events.

## Material and Methods

### Data collection

We performed a literature review using the database PubMed Central® (PMC). Initially, the identification of the published papers was carried out using the keywords “Chuvirus”, “Chuviridae” and “*Jingchuvirales*” and the Boolean operator “OR” for the combination of these three terms. With the results from this search, we performed a screening based on reading the title and abstract. The papers that corresponded with the manuscript goal were selected for a full reading, while those that did not, were removed from the study.

Only papers with the description of new chuvirus genomes published up to May 2021 were selected. The exclusion criteria were: i - papers describing only genomes from other viral families or with chuvirus genomes already available in previous papers; ii - published before 2015, the year of publication of the first original chuvirus genomes; iii - review articles, notes, and letters to the editor.

### Glycoprotein putative EVEs search

To identify putative *Jingchuvirales* glycoprotein-derived EVEs (Jg-EVEs) we used a BLASTp online approach. In this step, all *Jingchuvirales* glycoproteins identified in this study through literature mining were used as queries against the non-redundant (nr) protein database updated in May 2021 excluding all viruses from the subjects. The results were clustered to remove redundant hits using cd-hit with a sequence identity threshold and an alignment coverage threshold of 100% (-c 1 and -s 1) (Fu et al., 2012). The matching regions were reverse searched (tblastn) with correspondent genomes to select EVEs copies considering only matches with flanking regions of at least 10 kb. To analyze the Jg-EVEs boundaries, 10 kb upstream and downstream flanking regions were extracted using the bedtools flank (Quinlan and Hall, 2010).

The flanking regions were used in three analyses to understand the genomic context of each Jg-EVEs. The repeat content was evaluated using the RepeatMasker (Chen, 2004) online tool (default parameters, DNA source: fruit fly) and the results were analyzed using an in-house R script (repeatmasker2heatmap.R) to evaluate the frequency of repeat classes into Jg-EVEs boundaries, this evaluation considered the total region length with some repeat or transposon signature by each Jg-EVE, and the region length of each repeat or transposon associated to the specific Jg-EVE. The same flanking regions were submitted to a domain signature analysis to identify putative hybrid elements originating from the capture of Jg-EVE by retrotransposons, where the ORFs were extracted using Getorf (Emboss, 2022) (default parameters) and then analyzed with BATCH-CD-SEARCH (Marchler-Bauer and Bryant, 2004) (default parameters). Furthermore, we used cd-hit-est to identify clusters of Jg-EVEs plus flanking regions with a sequence identity threshold => 80% (-c 0.8) and an alignment coverage threshold of 20% based on the longer sequence (-aL 0.2) in the most accurate mode of clusterization (-g 1). Then, we used MAFFT (Katoh and Standley, 2013) with a global strategy to align the clusters to search for orthologous regions between arthropods genomes.

### Phylogenetic analyses

The RdRp protein was used to define the clades of the *Jingchuvirales* order and the glycoprotein was used to investigate the endogenization process across different eukaryotic groups. To reconstruct the RdRp phylogeny, RdRp from viruses belonging to Mononegavirales order according to ICTV (https://talk.ictvonline.org/) were recovered and clusterized (ictv_ncbi.py) from NCBI (https://www.ncbi.nlm.nih.gov/, last update at 2021 May) and were aligned separately by family. Each family alignment was automatically edited with CIAlign (Tumescheit et al., 2022), following the same strategy: an initial amino acid distance analysis followed by the automatic edition using the mean distance threshold for each family, the alignments were then concatenated and re-aligned. The RdRp of *Jingchuvirales* genomes bearing the three hallmark proteins (glycoprotein, nucleoprotein, and RNA-dependent RNA-Polymerase) or with genome length equal to or greater than 9 kb are aligned with the Mononegavirales reference alignment. Phylogenetic analysis of glycoproteins was performed with alignments encompassing all reference chuvirus glycoproteins retrieved from the literature, glycoproteins recovered from a previous study (Dezordi et al., 2020) and glycoproteins retrieved through the aforementioned strategy. 

Both nucleotide and amino acid alignments were performed with MAFFT, the substitution models were evaluated with ModelFinder (Kalyaanamoorthy et al., 2017). The RdRp of Mono-chu sequences was reconstructed with MrBayes 3.2.7a (Ronquist et al., 2012) with two independent runs, stop value equals to 0.0049 and 25% of burnin. The glycoprotein of *bona fide* viruses and putative EVEs was used to reconstruct a phylogenetic tree using IQ-TREE2 (Minh et al., 2020). Branch support was assessed by the ultrafast bootstrap method (Hoang et al., 2018) with 1000 replicates. All trees were rooted using midpoint-root and the annotation and visualization were performed using iTOL (Letunic and Bork, 2021).

## Results

**Figure 1 -:**
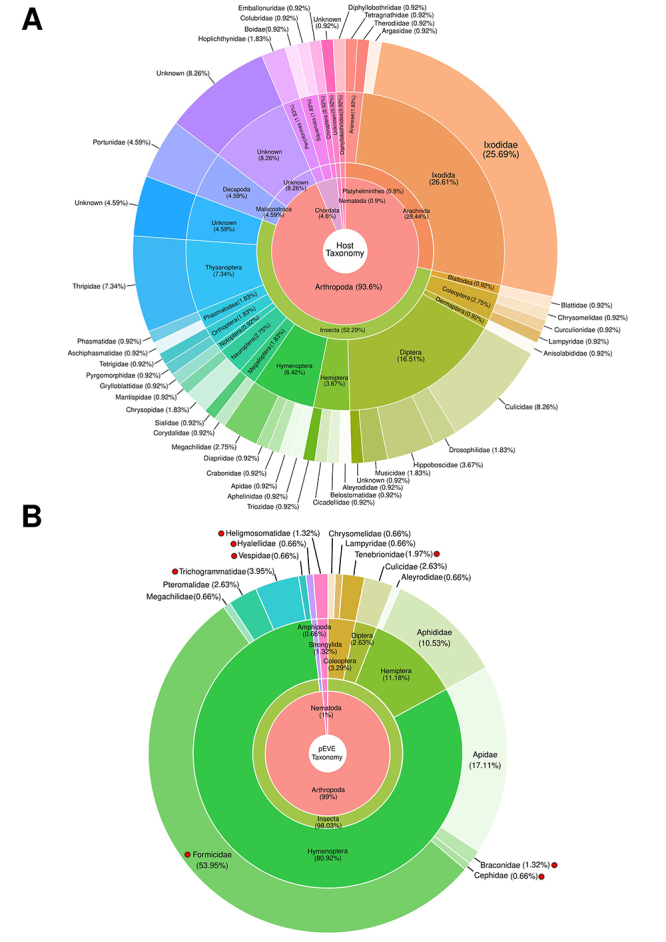
Host taxonomy of the *Jingchuvirales* order. **A.** Analyzed *Jingchuvirales* genomes (n = 109) sorted by different levels of host taxonomy. **B.** Jg-EVEs (n = 158), red circles represent host families where EVEs were found but there is no current evidence of exogenous *Jingchuvirales* infection. The percentage in each section of donut plots represents the total percentage of the section corresponding to the original entries of the innermost donut.

Keyword searches on the literature database resulted in 54 published studies (Table S1). After the full reading, only 21 met the inclusion criteria (Table 1). Since the first identification of chuviruses, 109 genomes associated with the *Chuviridae* family (Table S2) have been published of which 60 are complete genomes (carrying the tree hallmark proteins of the order - 73 nucleoproteins, 98 RNA-dependent RNA polymerase (RdRp), 79 glycoproteins Table S3). Of these, 49 were sequenced from samples of Araneae, Blattodea, Decapoda, Diptera, Hemiptera, Ixodida and Neuroptera orders which accounts for seven out of 26 known insect orders besides Perciformes and Squamata*.* The remaining eleven were obtained from unspecified hosts (Figure 1A).

**Table 1 -:** List of included studies that reported *Chuvirus* genomes.

ID^*^.Study	Year	New genomes	Countries	Host (Phylum)	Genome structure
1. Li et al., 2015	2015	22	China	Arthropoda	Circular: 17; linear: 5
2. Shi et al., 2016	2016	18	China	Arthropoda and nematoda	Linear: 17 P-circular: 1
3. Hang et al., 2016	2016	1	South Korea	Arthropoda	P-circular: 1
4. Pinto et al., 2017	2017	4	Brazil	Arthropoda	Linear: 4;
5. Souza et al., 2018	2018	8	Brazil	Arthropoda	P-circular: 8
6. Aiewsakun Simmonds, 2018	2018	18	China; USA	Arthropoda	Circular: 15 Linear: 3
7. Tokarz et al., 2018	2018	2	USA	Arthropoda	Circular: 2
8. Medd et al., 2018	2018	2	Japan; UK	Arthropoda	Linear: 2
9. Shi et al., 2018	2018	3	China	Chordata	Linear: 3
10. Harvey et al., 2019	2019	2	Australia	Arthropoda	P-circular: 2
11. Sameroff et al., 2019	2019	2	Trinidad and Tobago	Arthropoda	Circular: 2
12. Maia et al., 2019	2019	1	Brazil	Arthropoda	Linear: 1
13. Temmam et al., 2019	2019	2	Thailand	Arthropoda	Circular: 2
14. Käfer et al., 2019	2019	33	Germany; Austria; Venezuela; France; China; Japan; Australia; France; USA; Unknown**	Arthropoda	Linear: 33
15. Mishra et al., 2019	2019	1	Saudi Arabia	Chordata	Linear: 1
16. Thekke-Veetil *et al*., 2020	2020	9	USA	Arthropoda	Linear: 7 P-circular: 2
17. Gondard et al., 2020	2020	2	Guadeloupe: Martinique	Arthropoda	Circular: 2
18. Hahn et al., 2020	2020	1	USA	Platyhelminthes	Circular: 1
19. Argenta et al., 2020	2020	1	Brazil	Chordata	Linear: 1
20.Han et al., 2020	2020	1	China	Arthropoda	Linear: 1
21. Wu et al., 2021	2021	3	USA; Finland	Arthropoda	Linear: 3

**Figure 2 -:**
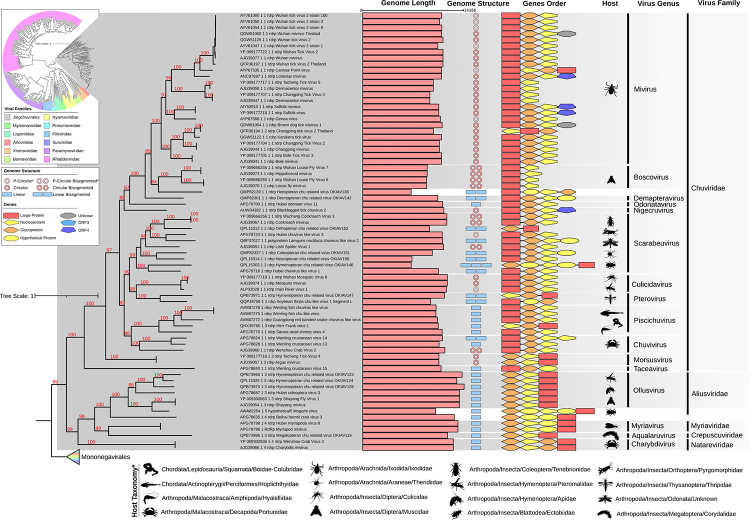
Bayesian phylogenetic tree of RdRp protein of *Jingchuvirales* and *Mononegavirales* orders. *Host Taxonomy updated at 2021 May according to NCBI information.

The RdRp phylogenetic tree confirmed the clustering of the new taxonomy of *Jingchuvirales* order proposed by ICTV (Figure 2), where the *Chuviridae* family comprises circular unsegmented genomes (Mivirus genus), circular bi-segmented genomes (Boscovirus), and a diversity genomes with linear or circular genomes and having 1 to 3 segments (other 10 genera). The *Aliusviridae, Myriaviridae*, *Crepuscuviridae* and *Natareviridae* are represented by viruses with linear segmented genomes.

**Figure 3 -:**
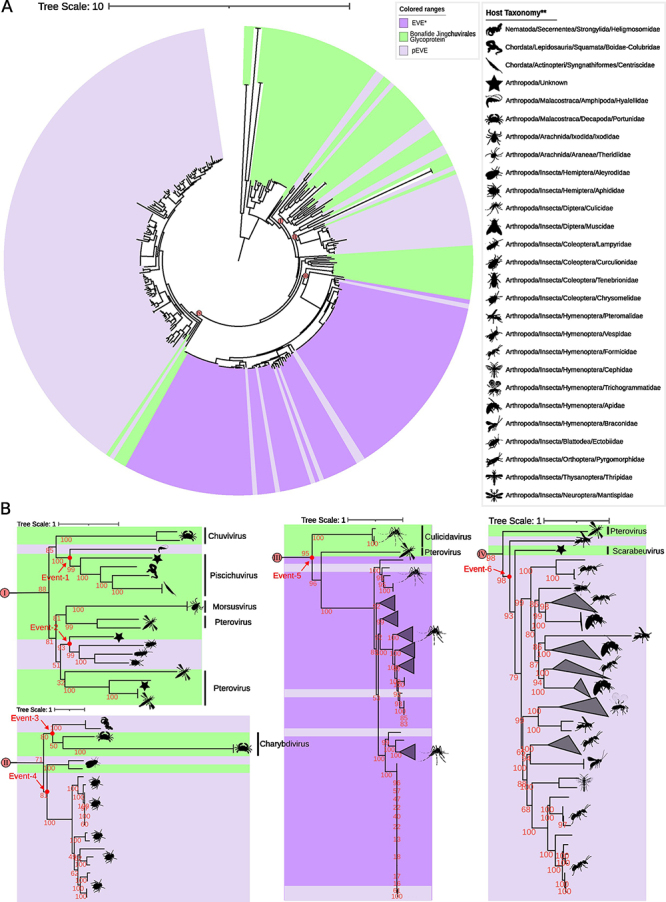
Maximum likelihood phylogenetic tree of the glycoprotein of Jingchuvirales order and putative representative Jg-EVEs. *EVEs recovered from Dezordi, et al., 2020. **Host Taxonomy updated at 2021 May according to NCBI information.

We found 158 EVEs (initial protein screening) representing 939 copies (screening against respective genome) in 38 species (Table S4). The glycoproteins phylogeny from *Jingchuvirales* and putative Jg-EVEs showed the existence of 6 distinct clades associated with endogenization events (Figure 3). From the six clades, one of them represents endogenization in Malacostraca with one Jg-EVE (Figure 3B, Event-1), one in Nematoda with two Jg-EVE (Figure 3B, Event-3), and four in Insecta (Figure 3B, Event-2, 4, 5 and 6). We found Jg-EVEs of the different genera on the identified events. The Event-1 is related to the Chuvivirus and Piscichuvirus genus of the *Chuviridae* family, Event-2, 5 and 6 are related to Pterovirus and other genera of the *Chuviridae* family, while the Event-3 to Charybdivirus genus (*Natareviridae* family) and the Event-4 to an unknown taxon of *Jingchuvirales* order.

**Figure 4:**
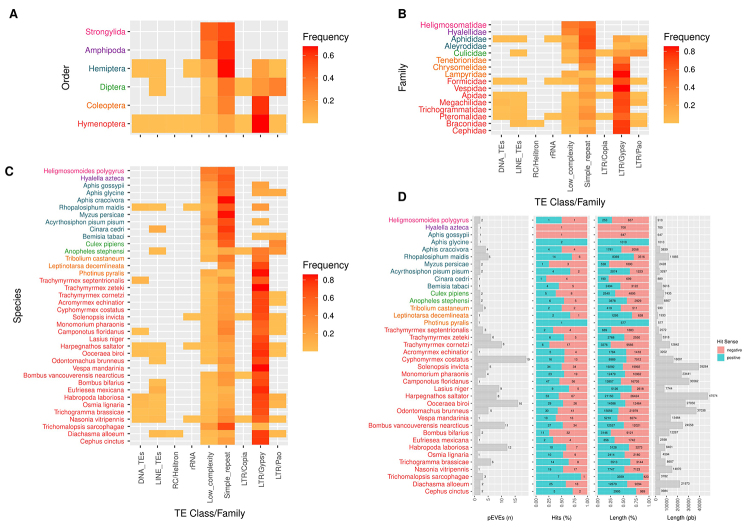
Jg-EVEs information. Heatmaps of frequency of TEs on EVEs boundaries at different taxonomy levels: (**A**) Order; (**B**) Family**;** (**C**) Species. (**D**) General information of representative EVEs hits against host genomes.

The Jg-EVEs of Malacostraca, Nematoda and some Insecta families are flanked by simple repeat and low complexity regions (Figure 4). On the other hand, several Jg-EVEs were flanked by LTR-Retrotranposon of Gypsy and BEL-Pao superfamilies in Hymenoptera, Culicidae and Coleoptera (Figure 4). The large majority of these associations occur between Jg-EVEs and fragmentary LTR retrotransposons copies (Table S5). However, we found two cases of Jg-EVEs in complete transposons boundaries, one in the species *Bemisia tabaci* (Unclassified element) and one Anakin (Dezordi et al., 2020) on *Anopheles stepehensi.*

Comparing a dataset of 939 Jg-EVEs copies and their flanking regions, we found 8 clusters with host genome ortholog regions in different species (Table 2). The multiple alignments of each cluster showed conserved flanking regions between species of the same genus (*Bombus* in clusters 263, 264, 265, 450, 451, 453 and 454) and from different genera of the same family (Pteromalidae in cluster 597) suggesting that the endogenization event involving these Jg-EVEs occurred in the ancestral species of the genus *Bombus* around 36~2 MyA and in the ancestor of the Pteromalidae family around 155~54.8 MyA (Kumar et al., 2017).

**Table 2 -:** Clusters of Jg-EVEs plus flanking regions.

Cluster number	Identity (%)	Genus	Sequences	LCA
263	89.72-99.86	*Bombus*	4	36~2MyA
264	83.64-94.28	*Bombus*	7	36~2MyA
265	90.08-91.89	*Bombus*	3	36~2MyA
450	81-57-86.88	*Bombus*	4	36~2MyA
451	84.88-99.52	*Bombus*	4	36~2MyA
453	82.85-87.06	*Bombus*	3	36~2MyA
454	80.96-99.53	*Bombus*	10	36~2MyA
597	80.24	*Cyphomyrmex* and *Odontomachus*	2	155~54.8MyA

## Discussion

Viruses leave traces of past infection in their host genomes as EVEs (Patel et al., 2011). These elements have only recently received considerable attention and extensive characterization revealed that all known viral families can be found integrated into diverse host genomes (Katzourakis and Gifford, 2010; Feschotte and Gilbert, 2012; Blair et al., 2020). Insects are infected by a large diversity of viral families and cognate EVEs have been found in many genomes, but EVEs from two viral families are particularly prevalent: *Rhabdoviridae* and *Chuviridae* (mostly glycoproteins) (Gilbert and Belliardo, 2022), raising questions about which host-virus features may be generating EVEs endogenization disparities between viral families (Wallau, 2022). But the large majority of studies did not characterize EVEs in detail to be able to investigate such questions (Palatini et al., 2022). Based on previous findings regarding the capture and amplification of Chuvirus glycoprotein by a retrotransposon in mosquito genomes we sought to investigate if the high content of Chuvirus glycoprotein EVEs also found in other arthropod genomes could be derived from the same retrotransposon capture phenomenon and more broadly characterize the timing and number of events within the insect’s evolutionary history.

Our results showed four Jingchuvirales glycoprotein endogenization events in 38 eukaryote genomes investigated. We were able to characterize four endogenization events that took place in the ancestors of several Insecta taxa. Jg-EVEs widespread distribution in extant insects may be a consequence of long-term vertical transmission since ancestral endogenizations. Ortholog copies of Jg-EVEs found between *Bombus* species (LCA 36~2 MYA) and *Cyphomyrmex* (Myrmicinae subfamily) and *Odontomachus* (Ponerinae subfamily) (LCA 155~54.8 MyA) add further evidence for ancient integration events and long term vertical transmission since the Eocene and lower Cretaceous (Kumar et al., 2017). But studies focusing on high-quality genomes of specific host taxa should be performed to obtain more precise endogenization timing estimates. All EVEs characterized were derived from two out of five currently recognized families of the order Jungchuvirales (*Chuviridae* and *Netaviridae*). Therefore, there is no specific association between EVE and host taxa and the higher number of endogenization events derived from the *Chuviridae* family may be simply a result of its larger host range (Figure 1). Transposable elements and other repetitive sequences have been found in association with EVEs in several insect species (Whitfield et al., 2017; Ter Horst et al., 2019) suggesting that these repetitive endogenous sequences are mediating viral segment integration in the host genome (Tassetto et al., 2019) and that EVEs sequences and proteins may be co-opted as new genes of the host genome or captured by endogenous retrotransposons (Feschotte and Gilbert, 2012). Our analysis of Jg-EVEs showed that LTR retrotransposons of Gypsy, Copia, and Pao families are particularly enriched in their flanking regions. The first is highly prevalent in Hymenoptera and Coleoptera species while Copia and Pao are more clearly associated with Diptera species. However, despite such association, we found no clear evidence of Jungchuvirales glycoprotein capture by retrotransposons for Gypsy and Copia family other than the Pao retrotransposon capture of a Chuvirus-derived protein previously found in mosquitos by our group (Dezordi et al., 2020).

EVEs are equivalent to genetic fossils, and as such, they store information about past or extant viral infections as well as providing additional information about viral host range (Katzourakis and Gifford, 2010). Based on the phylogenetic relationships of Jg-EVEs and circulating viruses it is possible to infer that certain virus lineages may infect previously unknown host taxa. We found several EVEs in wasps (Vespidae) and ants (Formicidae) species (Figure 1B and Figure 3) that were not found naturally infected by Jingchuviralres viruses so far (Figure 2, Figure 1A) suggesting that these species were or still are infected by viruses from this order (Figure 2, Ollusvirus clade identified in Pteromalidae and Apidae, and Culicidavirus clade indentied in Pteromalidae).

The diversity of genomic structures of the *Chuviridae f*amily cover circular, linear, segmented and non-segmented genomes, which is particularly unusual for RNA viruses (Li et al., 2015). These authors proposed a phylogenetic model in which segmented and non-segmented chuviruses genomes are in an intermediate position between linear and circular genomes. However, it’s possible that such an organization follows an intrinsic evolutionary pattern within the *Jingchuvirales* order. Our RdRp phylogeny (Figure 2 **)** shows a different clade organization with clades exclusively with linear genomes and others with circular genomes. The new families *Aliusviridae, Myriaviridae, Crepuscuviridae* and *Natareviridae* proposed by ICTV are composed only of linear genomes arranged in specific clades of the *Jingrchuvirales* order (Figure 2, Figure S1).

In this study, we identified several Jg-EVEs across eukaryote genomes. These elements originated in the ancient past through six distinct integration events, the majority occurring in insects. Despite the presence of TEs on EVEs boundaries, we found no evidence of glycoprotein capture by retrotransposons in other insect species except by the already characterized event in Culicidae. Therefore, new studies are warranted to better understand the deep relationships and long-term maintenance of *Jingchuvirales* glycoproteins EVEs in insect genomes.

## Supplementary material

The following online material is available for this article:

Table S1 - PMC results of keyword research.

Table S2 - Studies included in the genomes collection.

Table S3 - Viruses included in this study.

Table S4 - Host genomes and EVEs copies.

Table S5 - Endogenous viruses flanking regions structures.

Figure S1 - Jingchuvirales phylogeny from different studies.
