# Supplementary material for: Ancient origin of Jingchuvirales derived glycoproteins integrated in arthropod genomes
Source: Genet Mol Biol. 2023 Apr 7;46(1):e20220218. doi: 10.1590/1678-4685-GMB-2022-0218 (PMC10084718; doi:10.1590/1678-4685-GMB-2022-0218)
Supplement: Table S1 - [file 1415-4757-GMB-46-1-e20220218-s1.pdf]

Genetics and Molecular Biology
https://doi.org/10.1590/1678-4685-GMB-2022-0218

Supplementary Material to "Ancient origin of Jingchuvirales derived glycoproteins integrated in arthropod genomes"

Table S1 - PMC results of keyword research.

Study	PMCID
Virome composition in marine fish revealed by meta-transcriptomics	PMC7887440
Decoding the RNA viromes in rodent lungs provides new insight into the origin and evolutionary patterns of rodent-borne pathogens in Mainland Southeast Asia	PMC7818139
Soybean Thrips (Thysanoptera: Thripidae) Harbor Highly Diverse Populations of Arthropod, Fungal and Plant Viruses	PMC7761488
In and Outs of Chuviridae Endogenous Viral Elements: Origin of a Potentially New Retrovirus and Signature of Ancient and Ongoing Arms Race in Mosquito Genomes	PMC7642597
Unmapped RNA Virus Diversity in Termites and Their Symbionts	PMC7650761
Stability of the Virome in Lab- and Field-Collected Aedes albopictus Mosquitoes across Different Developmental Stages and Possible Core Viruses in the Publicly Available Virome Data of Aedes Mosquitoes	PMC7527137
Improved reference genome of the arboviral vector Aedes albopictus	PMC7448346
Abundant and Diverse RNA Viruses in Insects Revealed by RNA-Seq Analysis: Ecological and Evolutionary Implications	PMC7343303
Comparative Analysis of RNA Virome Composition in Rabbits and Associated Ectoparasites	PMC7269439
Identification of Reptarenaviruses, Hartmaniviruses, and a Novel Chuvirus in Captive Native Brazilian Boa Constrictors with Boid Inclusion Body Disease	PMC7269426
Characterization of viruses in a tapeworm: phylogenetic position, vertical transmission, and transmission to the parasitized host	PMC7305300
Special Issue “Emerging Viruses: Surveillance, Prevention, Evolution, and Control”	PMC7150905
Global Organization and Proposed Megataxonomy of the Virus World	PMC7062200
RNA Viruses of Amblyomma variegatum and Rhipicephalus microplus and Cattle Susceptibility in the French Antilles	PMC7077237
Analysis of the RNA virome of basal hexapods	PMC6955108
Identification and characterisation of common glow-worm RNA viruses	PMC7093385
Novel Viruses Found in Antricola Ticks Collected in Bat Caves in the Western Amazonia of Brazil	PMC7019218
Re-assessing the diversity of negative strand RNA viruses in insects	PMC6932829
Monitoring Silent Spillovers Before Emergence: A Pilot Study at the Tick/Human Interface in Thailand	PMC6812269
Novel Viruses in Mosquitoes from Brazilian Pantanal	PMC6832572
Metaviromics Reveals Unknown Viral Diversity in the Biting Midge Culicoides impunctatus	PMC6784199

Study	PMCID
Stable distinct core eukaryotic viromes in different mosquito species from Guadeloupe, using single mosquito viral metagenomics	PMC6714450
Viral Diversity of Tick Species Parasitizing Cattle and Dogs in Trinidad and Tobago	PMC6639388
RNA Synthesis and Capping by Non-segmented Negative Strand RNA Viral Polymerases: Lessons From a Prototypic Virus	PMC6636387
Novel insights into endogenous RNA viral elements in Ixodes scapularis and other arbovirus vector genomes	PMC6580184
The Ecology of New Constituents of the Tick Virome and Their Relevance to Public Health	PMC6630940
Assessing the Diversity of Endogenous Viruses Throughout Ant Genomes	PMC6540820
Fecal Viral Diversity of Captive and Wild Tasmanian Devils Characterized Using Virion-Enriched Metagenomics and Metatranscriptomics	PMC6532096
A viral metagenomic survey identifies known and novel mammalian viruses in bats from Saudi Arabia	PMC6457491
Extensive Diversity of RNA Viruses in Australian Ticks	PMC6340049
Mapping Arbovirus-Vector Interactions Using Systems Biology Techniques	PMC6330711
Genomic characterization, phylogenetic position and in situ localization of a novel putative mononegavirus in Lepeophtheirus salmonis	PMC6394706
Viral diversity of Rhipicephalus microplus parasitizing cattle in southern Brazil	PMC6218518
Metagenomic sequencing suggests a diversity of RNA interference-like responses to viruses across multicellular eukaryotes	PMC6085071
The Expanding Diversity of RNA Viruses in Vertebrates	PMC7125577
Viral Diversity of House Mice in New York City	PMC5904411
A58 Identification of novel viruses in the families Flaviviridae (Jigmenvirus), Chuviridae, and Bunyaviridae (phlebovirus-like) in ticks from the south of Brazil	PMC5905370
The virome of Drosophila suzukii, an invasive pest of soft fruit	PMC5888908
Mapping the virome in wild-caught Aedes aegypti from Cairns and Bangkok	PMC5856816
Tick-Borne Viruses	PMC5866268
Identification of Novel Viruses in Amblyomma americanum, Dermacentor variabilis, and Ixodes scapularis Ticks	PMC5853492
The genomic underpinnings of eukaryotic virus taxonomy: creating a sequence-based framework for family-level virus classification	PMC5819261
A global ocean atlas of eukaryotic genes	PMC5785536
Presence of Apis Rhabdovirus-1 in Populations of Pollinators and Their Parasites from Two Continents	PMC5732965
The diversity, structure and function of heritable adaptive immunity sequences in the Aedes aegypti genome	PMC5698160
Novel viruses in salivary glands of mosquitoes from sylvatic Cerrado, Midwestern Brazil	PMC5678729
Metagenomics reshapes the concepts of RNA virus evolution by revealing extensive horizontal virus transfer	PMC5801114
An RNA Virome Associated to the Golden Orb-Weaver Spider Nephila clavipes	PMC5660997
A decade of RNA virus metagenomics is (not) enough	PMC7114529
High-Resolution Metatranscriptomics Reveals the Ecological Dynamics of Mosquito-Associated RNA Viruses in Western Australia	PMC5553174
2016 International meeting of the Global Virus Network	PMC7113740

Study	PMCID
Genome Sequences of Five Arboviruses in Field-Captured Mosquitoes in a Unique Rural Environment of South Korea	PMC4732341
The evolution, diversity, and host associations of rhabdoviruses	PMC5014481
Unprecedented genomic diversity of RNA viruses in arthropods reveals the ancestry of negative-sense RNA viruses	PMC4384744
