# Supplementary material for: Ancient origin of Jingchuvirales derived glycoproteins integrated in arthropod genomes
Source: Genet Mol Biol. 2023 Apr 7;46(1):e20220218. doi: 10.1590/1678-4685-GMB-2022-0218 (PMC10084718; doi:10.1590/1678-4685-GMB-2022-0218)
Supplement: Table S2 - [file 1415-4757-GMB-46-1-e20220218-s2.pdf]

Genetics and Molecular Biology

https://doi.org/10.1590/1678-4685-GMB-2022-0218

Supplementary Material to "Ancient origin of Jingchuvirales derived glycoproteins integrated in arthropod genomes"

Table S2 - Studies included in the genomes collection.

paper_id	virus_id	virus_access	virus_name	host	host_phylum	host_class	host_order	host_family	country	methods
1	1	NC_028259	Bole_Tick_Virus_3	Hyalomma asiaticum	Arthropoda	Arachnida	Ixodida	Ixodidae	China	RNA-Seq; Denovo Assembly; BLASTx comparrison
1	2	NC_028260	Changping_Tick_Virus_2	Dermacentor sp.	Arthropoda	Arachnida	Ixodida	Ixodidae	China	RNA-Seq; Denovo Assembly; BLASTx comparrison
1	3	NC_028261	Changping_Tick_Virus_3	Dermacentor sp.	Arthropoda	Arachnida	Ixodida	Ixodidae	China	RNA-Seq; Denovo Assembly; BLASTx comparrison
1	04.01	KM817597.1	Lishi_Spider_Virus_1	Parasteatoda tepidariorum	Arthropoda	Arachnida	Araneae	Theridiidae	China	RNA-Seq; Denovo Assembly; BLASTx comparrison
1	04.02	KM817596.1	Lishi_Spider_Virus_1	Parasteatoda tepidariorum	Arthropoda	Arachnida	Araneae	Theridiidae	China	RNA-Seq; Denovo Assembly; BLASTx comparrison
1	5	NC_031214	Shayang_Fly_Virus_1	Atherigona orientalis	Arthropoda	Insecta	Diptera	Musicidae	China	RNA-Seq; Denovo Assembly; BLASTx comparrison
1	6	KM817612	Shuangao_Fly_Virus_1	Unidentified Diptera	Arthropoda	Insecta	Diptera	Unknown	China	RNA-Seq; Denovo Assembly; BLASTx comparrison
1	7	KM817614	Shuangao_Insect_Virus_5	Unidentified Diptera, Abraxas tenuisuffusa, Unidentified Chrysopidae	Arthropoda	Insecta	Unknown	Unknown	China	RNA-Seq; Denovo Assembly; BLASTx comparrison
1	8	KR902734.1	Shuangao_Lacewing_Virus	Unidentified Chrysopidae	Arthropoda	Insecta	Neuroptera	Chrysopidae	China	RNA-Seq; Denovo Assembly; BLASTx comparrison

paper_id	virus_id	virus_access	virus_name	host	host_phylum	host_class	host_order	host_family	country	methods
1	9	NC_028263.1	Tacheng_Tick_Virus_4	Argas miniatus	Arthropoda	Arachnida	Ixodida	Ixodidae	China	RNA-Seq; Denovo Assembly; BLASTx comparrison
1	10	NC_028264.1	Tacheng_Tick_Virus_5	Dermacentor marginatus	Arthropoda	Arachnida	Ixodida	Ixodidae	China	RNA-Seq; Denovo Assembly; BLASTx comparrison
1	11.1	KM817601.1	Wenzhou_Crab_Virus_2	Charybdis japonica, Charybdis lucifera, Charybdis hellerii	Arthropoda	Malacostraca	Decapoda	Portunidae	China	RNA-Seq; Denovo Assembly; BLASTx comparrison
1	11.2	KM817602.1	Wenzhou_Crab_Virus_2	Charybdis japonica, Charybdis lucifera, Charybdis hellerii	Arthropoda	Malacostraca	Decapoda	Portunidae	China	RNA-Seq; Denovo Assembly; BLASTx comparrison
1	12	NC_031248.1	Wenzhou_Crab_Virus_3	Charybdis japonica	Arthropoda	Malacostraca	Decapoda	Portunidae	China	RNA-Seq; Denovo Assembly; BLASTx comparrison
1	13.1	NC_043472.1	Wuchang_Cockroach_Virus_3	Blattella germanica	Arthropoda	Malacostraca	Decapoda	Portunidae	China	RNA-Seq; Denovo Assembly; BLASTx comparrison
1	13.2	NC_043473.1	Wuchang_Cockroach_Virus_3	Blattella germanica	Arthropoda	Malacostraca	Decapoda	Portunidae	China	RNA-Seq; Denovo Assembly; BLASTx comparrison
1	14.1	NC_043476.1	Wuhan_Louse_Fly_Virus_6	Unidentified Hippoboscidae	Arthropoda	Insecta	Diptera	Hippoboscidae	China	RNA-Seq; Denovo Assembly; BLASTx comparrison
1	14.2	NC_043475	Wuhan_Louse_Fly_Virus_6	Unidentified Hippoboscidae	Arthropoda	Insecta	Diptera	Hippoboscidae	China	RNA-Seq; Denovo Assembly; BLASTx comparrison
1	15.1	NC_043471.1	Wuhan_Louse_Fly_Virus_7	Unidentified Hippoboscidae	Arthropoda	Insecta	Diptera	Hippoboscidae	China	RNA-Seq; Denovo Assembly; BLASTx comparrison
1	15.2	NC_043470.1	Wuhan_Louse_Fly_Virus_7	Unidentified Hippoboscidae	Arthropoda	Insecta	Diptera	Hippoboscidae	China	RNA-Seq; Denovo Assembly; BLASTx comparrison

paper_id	virus_id	virus_access	virus_name	host	host_phylum	host_class	host_order	host_family	country	methods
1	16	NC_028265.1	Wuhan_Mosquito_Virus_8	Culex tritaeniorhynchus, Cx. Quinquefasciatus, Anopheles sinensis, Armigeres subalbatus	Arthropoda	Insecta	Diptera	Culicidae	China	RNA-Seq; Denovo Assembly; BLASTx comparrison
1	17	NC_028266	Wuhan_Tick_Virus_2	Rhipicephalus microplus	Arthropoda	Arachnida	Ixodida	Ixodidae	China	RNA-Seq; Denovo Assembly; BLASTx comparrison
2	18.1	KX884424	Hubei_chuvirus-like_virus_1	Odonata	Arthropoda	Insecta	Unknown	Unknown	China	Trinityrnaseq v. 2.0.6; Illumina sequencing
2	18.2	KX884425	Hubei_chuvirus-like_virus_1	Odonata	Arthropoda	Insecta	Unknown	Unknown	China	Trinityrnaseq v. 2.0.6; Illumina sequencing
2	19	KX884427	Hubei_chuvirus-like_virus_3	Odonata	Arthropoda	Insecta	Unknown	Unknown	China	Trinityrnaseq v. 2.0.6; Illumina sequencing
2	20	KX884438	Hubei_chuvirus-like_virus_4	Tetragnatha maxillosa	Arthropoda	Arachnida	Araneae	Tetragnathidae	China	Trinityrnaseq v. 2.0.6; Illumina sequencing
2	21	KX884449	Wenling_chuvirus-like_virus_1	crustacean	Arthropoda	Unknown	Unknown	Unknown	China	Trinityrnaseq v. 2.0.6; Illumina sequencing
2	22	KX884455	Wenling_chuvirus-like_virus_2	crustacean	Arthropoda	Unknown	Unknown	Unknown	China	Trinityrnaseq v. 2.0.6; Illumina sequencing
2	23	KX884419	Hubei_odonate_virus_11	Odonata	Arthropoda	Insecta	Unknown	Unknown	China	Trinityrnaseq v. 2.0.6; Illumina sequencing
2	24	KX884439	Sanxia_atyid_shrimp_virus_4	freshwater atyid shrimp	Arthropoda	Unknown	Unknown	Unknown	China	Trinityrnaseq v. 2.0.6; Illumina sequencing
2	25.1	KX884451	Wenling_crustacean_virus_14	crustacean	Arthropoda	Unknown	Unknown	Unknown	China	Trinityrnaseq v. 2.0.6; Illumina sequencing
2	25.2	KX884452	Wenling_crustacean_virus_14	crustacean	Arthropoda	Unknown	Unknown	Unknown	China	Trinityrnaseq v. 2.0.6; Illumina sequencing
2	26.1	KX884453	Wenling_crustacean_virus_13	crustacean	Arthropoda	Unknown	Unknown	Unknown	China	Trinityrnaseq v. 2.0.6; Illumina sequencing

paper_id	virus_id	virus_access	virus_name	host	host_phylum	host_class	host_order	host_family	country	methods
2	26.2	KX884454	Wenling_crustacean_virus_13	crustacean	Arthropoda	Unknown	Unknown	Unknown	China	Trinityrnaseq v. 2.0.6; Illumina sequencing
2	27	KX884409	Beihai_barnacle_virus_9	barnacle	Arthropoda	Unknown	Unknown	Unknown	China	Trinityrnaseq v. 2.0.6; Illumina sequencing
2	28	KX884458	Wenling_crustacean_virus_15	crustacean	Arthropoda	Unknown	Unknown	Unknown	China	Trinityrnaseq v. 2.0.6; Illumina sequencing
2	29	KX884461	Xinzhou_nematode_virus_5	snake-associated nematodes	Nematoda	Unknown	Unknown	Unknown	China	Trinityrnaseq v. 2.0.6; Illumina sequencing
2	30	KX884444	Hubei_myriapoda_virus_8	Myriapoda	Arthropoda	Unknown	Unknown	Unknown	China	Trinityrnaseq v. 2.0.6; Illumina sequencing
2	31	KX884404	Beihai_hermit_crab_virus_3	hermit crab	Arthropoda	Unknown	Unknown	Unknown	China	Trinityrnaseq v. 2.0.6; Illumina sequencing
2	32	KX884416	Hubei_coleoptera_virus_3	Coleoptera	Arthropoda	Insecta	Unknown	Unknown	China	Trinityrnaseq v. 2.0.6; Illumina sequencing
3	33	KU095839	Imjin_River_virus_1	Culex bitaeniorhynchus	Arthropoda	Insecta	Diptera	Culicidae	South Korea	Roche GS analysis software v2.9; Ray de novo genome assembler v2.2; Illumina sequencing
4	34	MF344589	Kaiowa_virus	Stegomyia albopicta and Ochlerotatus sp. pool	Arthropoda	Insecta	Diptera	Culicidae	Brazil	Geneious v. 9.1.5; Illumina sequencing
4	35	MF344596	Cumbaru_virus	Mansonia wilsoni	Arthropoda	Insecta	Diptera	Culicidae	Brazil	Geneious v. 9.1.5; Illumina sequencing
4	36	MF344588	Croada_virus	Psorophora spp.	Arthropoda	Insecta	Diptera	Culicidae	Brazil	Geneious v. 9.1.5; Illumina sequencing
4	37	MF344590	Kaiowa_virus	Stegomyia albopicta	Arthropoda	Insecta	Diptera	Culicidae	Brazil	Geneious v. 9.1.5; Illumina sequencing
4	38	MH155927	Wuhan_tick_virus_2_strain_100	Rhipicephalus microplus	Arthropoda	Arachnida	Ixodida	Ixodidae	Brazil	Spades v. 3.10; IDBA-UD v. 1.1.1.; GARM v. 0.7.3; Illumina sequencing

paper_id	virus_id	virus_access	virus_name	host	host_phylum	host_class	host_order	host_family	country	methods
5	39.1	MH155926	Wuhan_tick_virus_2_strain_11	Rhipicephalus microplus	Arthropoda	Arachnida	Ixodida	Ixodidae	Brazil	Spades v. 3.10; IDBA-UD v. 1.1.1.; GARM v. 0.7.3; Illumina sequencing
5	39.2	MH155925	Wuhan_tick_virus_2_strain_11	Rhipicephalus microplus	Arthropoda	Arachnida	Ixodida	Ixodidae	Brazil	Spades v. 3.10; IDBA-UD v. 1.1.1.; GARM v. 0.7.3; Illumina sequencing
5	39.3	MH155924	Wuhan_tick_virus_2_strain_11	Rhipicephalus microplus	Arthropoda	Arachnida	Ixodida	Ixodidae	Brazil	Spades v. 3.10; IDBA-UD v. 1.1.1.; GARM v. 0.7.3; Illumina sequencing
5	40	MH155923	Wuhan_tick_virus_2__strain_9	Rhipicephalus microplus	Arthropoda	Arachnida	Ixodida	Ixodidae	Brazil	Spades v. 3.10; IDBA-UD v. 1.1.1.; GARM v. 0.7.3; Illumina sequencing
5	41	MH155922	Wuhan_tick_virus_2_strain_5	Rhipicephalus microplus	Arthropoda	Arachnida	Ixodida	Ixodidae	Brazil	Spades v. 3.10; IDBA-UD v. 1.1.1.; GARM v. 0.7.3; Illumina sequencing
5	42	MH155921	Wuhan_tick_virus_2_strain_3	Rhipicephalus microplus	Arthropoda	Arachnida	Ixodida	Ixodidae	Brazil	Spades v. 3.10; IDBA-UD v. 1.1.1.; GARM v. 0.7.3; Illumina sequencing
5	43	MH155920	Wuhan_tick_virus_2_strain_1	Rhipicephalus microplus	Arthropoda	Arachnida	Ixodida	Ixodidae	Brazil	Spades v. 3.10; IDBA-UD v. 1.1.1.; GARM v. 0.7.3; Illumina sequencing
6	44	KM817599	Argas_mivirus	Argas miniatus	Arthropoda	Arachnida	Ixodida	Argasidae	China	Sanger dideoxy sequencing
6	45	KM817593	Bole_mivirus	Hyalomma asiaticum	Arthropoda	Arachnida	Ixodida	Ixodidae	China	Sanger dideoxy sequencing
6	46	KM817594	Changping_mivirus	Dermacentor sp	Arthropoda	Arachnida	Ixodida	Ixodidae	China	Sanger dideoxy sequencing
6	47	KM817603	Charybdis_mivirus	Charybdis japonica	Arthropoda	Malacostraca	Decapoda	Portunidae	China	Sanger dideoxy sequencing
6	48.1	KM817604	Cockroach_mivirus	Blattella germanica	Arthropoda	Insecta	Blattodea	Portunidae	China	Sanger dideoxy sequencing
6	48.2	KM817605	Cockroach_mivirus	Blattella germanica	Arthropoda	Insecta	Blattodea	Portunidae	China	Sanger dideoxy sequencing
6	49.1	KM817595	Dermacentor_mivirus	Dermacentor sp	Arthropoda	Arachnida	Ixodida	Ixodidae	China	Sanger dideoxy sequencing

paper_id	virus_id	virus_access	virus_name	host	host_phylum	host_class	host_order	host_family	country	methods
6	49.2	KM817600	Dermacentor_mivirus	Dermacentor sp unidentified	Arthropoda	Arachnida	Ixodida	Ixodidae	China	Sanger dideoxy sequencing
6	50.1	KM817609	Hippoboscid_mivirus	Hippoboscidae unidentified	Arthropoda	Insecta	Diptera	Hippoboscidae	China	Sanger dideoxy sequencing
6	50.2	KM817608	Hippoboscid_mivirus	Hippoboscidae unidentified	Arthropoda	Insecta	Diptera	Hippoboscidae	China	Sanger dideoxy sequencing
6	51	KM817613	Lacewing_mivirus	Chrysopidae	Arthropoda	Insecta	Neuroptera	Chrysopidae	China	Sanger dideoxy sequencing
6	52	KU230451	Lonestar_mivirus	Amblyomma americanum	Arthropoda	Arachnida	Ixodida	Ixodidae	USA	Geneious v. 6.1.5; Illumina sequencing
6	53.1	KM817606	Louse_fly_mivirus	unidentified Hippoboscidae	Arthropoda	Insecta	Diptera	Hippoboscidae	China	Sanger dideoxy sequencing
6	53.2	KM817607	Louse_fly_mivirus	unidentified Hippoboscidae	Arthropoda	Insecta	Diptera	Hippoboscidae	China	Sanger dideoxy sequencing
6	54	KM817610	Mosquito_mivirus	Culex tritaeniorhynchus	Arthropoda	Insecta	Diptera	Culicidae	China	Sanger dideoxy sequencing
6	55	KM817598	Shayang_mivirus	Atherigona orientalis	Arthropoda	Insecta	Diptera	Musicidae	China	Sanger dideoxy sequencing
6	56	KM460042	Suffolk_mivirus	Ixodes scapularis	Arthropoda	Arachnida	Ixodida	Ixodidae	USA	Geneious v. 6.1.5; Sanger dideoxy sequencing
6	57	KM817611	Wuhan_mivirus	Rhipicephalus microplus	Arthropoda	Arachnida	Ixodida	Ixodidae	China	Sanger dideoxy sequencing
7	58	NC028243	Suffolk_virus	Ixodes scapularis	Arthropoda	Arachnida	Ixodida	Ixodidae	USA	Geneious v. 6.1.5; Illumina sequencing
7	59	MF360789	Blacklegged_tick_chuvirus_2	Ixodes scapularis	Arthropoda	Arachnida	Ixodida	Ixodidae	USA	Geneious v. 10.1.2; Illumina sequencing
8	60	MF893252	Mogami_virus	Drosophila suzukii	Arthropoda	Insecta	Diptera	Drosophilidae	Japan	Trinity v. 2.2.0; Illumina sequencing; Detected by RT-PCR
8	61	MF893248	Kiln_Barn_virus	Drosophila suzukii	Arthropoda	Insecta	Diptera	Drosophilidae	United Kingdom	Trinity v. 2.2.0; Illumina sequencing; Detected by RT-PCR
9	62	MK026566.1	Cannae_Point_virus	Amblyomma moreliae	Arthropoda	Arachnida	Ixodida	Ixodidae	Australia	Trinity v. 2.1.1; Illumina sequencing

paper_id	virus_id	virus_access	virus_name	host	host_phylum	host_class	host_order	host_family	country	methods
9	63	MK026591.1	Genoa_virus	Ixodes holocyclus	Arthropoda	Arachnida	Ixodida	Ixodidae	Australia	Trinity v. 2.1.1; Illumina sequencing
10	64	MN025520.1	Brown_dog_tick_mivirus_1	Rhipicephalus sanguineus	Arthropoda	Arachnida	Ixodida	Ixodidae	Trinidad and Tobago	MIRA v. 4.0; Megahit v. 1.1.X; Illumina sequencing
10	65	MN025521.1	Wuhan_mivirus_(Trinidad)	Rhipicephalus microplus	Arthropoda	Arachnida	Ixodida	Ixodidae	Trinidad and Tobago	MIRA v. 4.0; Megahit v. 1.1.X; Illumina sequencing
11	66	MK780200	Uru_chuvirus	Sabethes gymnothorax; female	Arthropoda	Insecta	Diptera	Culicidae	Brazil	Geneious v. 11.1.5; Illumina sequencing
12	67	MN095546	Wuhan_tick_virus_2-Thailand	Boophilus sp	Arthropoda	Arachnida	Ixodida	Ixodidae	Thailand	Megahit v. 1.1.2; Illumina sequencing
12	68	MN095545	Changping_tick_virus_2-Thailand	Rhipicephalus sanguineus	Arthropoda	Arachnida	Ixodida	Ixodidae	Thailand	Megahit v. 1.1.2; Illumina sequencing
13	69.1	MT153403.1	Coleopteran_chu-related_virus_OKIAV127	Oreina cacaliae	Arthropoda	Insecta	Coleoptera	Chrysomelidae	Germany	SOAPdenovo-Trans-31kmer v. 1.01; Illumina sequencing
13	69.2	MT153467.1	Coleopteran_chu-related_virus_OKIAV127	Oreina cacaliae	Arthropoda	Insecta	Coleoptera	Chrysomelidae	Germany	SOAPdenovo-Trans-31kmer v. 1.01; Illumina sequencing
13	70	MW288217.1	Megalopteran_chu-related_virus_OKIAV130	Sialis lutaria	Arthropoda	Insecta	Megaloptera	Sialidae	Austria	SOAPdenovo-Trans-31kmer v. 1.01; Illumina sequencing
13	71	MT153458.1	Orthopteran_chu-related_virus_OKIAV116	Tetrix subulata	Arthropoda	Insecta	Orthoptera	Tetrigidae	Germany	SOAPdenovo-Trans-31kmer v. 1.01; Illumina sequencing
13	72	MT153417.1	Blattodean_chu-related_virus_OKIAV148	Periplaneta americana	Arthropoda	Insecta	Blattodea	Blattidae	Germany	SOAPdenovo-Trans-31kmer v. 1.01; Illumina sequencing
13	73	MW039261.1	Megalopteran_chu-related_virus_OKIAV119	Corydalidae sp.	Arthropoda	Insecta	Megaloptera	Corydalidae	Venezuela	SOAPdenovo-Trans-31kmer v. 1.01; Illumina sequencing
13	74	MW039254.1	Hymenopteran_chu-related_virus_OKIAV123	Dioxys cincta	Arthropoda	Insecta	Hymenoptera	Megachilidae	France	SOAPdenovo-Trans-31kmer v. 1.01; Illumina sequencing
13	75	MW039256.1	Hymenopteran_chu-related_virus_OKIAV126	Oxybelus bipunctatus	Arthropoda	Insecta	Hymenoptera	Crabronidae	Germany	SOAPdenovo-Trans-31kmer v. 1.01; Illumina sequencing
13	76.1	MW288199.1	Hemipteran_chu-related_virus_OKIAV140	Diplonychus rusticus	Arthropoda	Insecta	Hemiptera	Belostomatidae	China	SOAPdenovo-Trans-31kmer v. 1.01; Illumina sequencing

paper_id	virus_id	virus_access	virus_name	host	host_phylum	host_class	host_order	host_family	country	methods
13	76.2	MW288213.1	Hemipteran_chu-related_virus_OKIAV140	Diplonychus rusticus	Arthropoda	Insecta	Hemiptera	Belostomatidae	China	SOAPdenovo-Trans-31kmer v. 1.01; Illumina sequencing
13	77.1	MW288180.1	Neuropteran_chu-related_virus_OKIAV150	Eumantispa harmandi	Arthropoda	Insecta	Neuroptera	Mantispidae	Japan	SOAPdenovo-Trans-31kmer v. 1.01; Illumina sequencing
13	77.2	MW288234.1	Neuropteran_chu-related_virus_OKIAV150	Eumantispa harmandi	Arthropoda	Insecta	Neuroptera	Mantispidae	Japan	SOAPdenovo-Trans-31kmer v. 1.01; Illumina sequencing
13	78.1	MT153371.1	Hemipteran_chu-related_virus_OKIAV138	Trialeurodes vaporariorum	Arthropoda	Insecta	Hemiptera	Aleyrodidae	Germany	SOAPdenovo-Trans-31kmer v. 1.01; Illumina sequencing
13	78.2	MT153353.1	Hemipteran_chu-related_virus_OKIAV138	Trialeurodes vaporariorum	Arthropoda	Insecta	Hemiptera	Aleyrodidae	Germany	SOAPdenovo-Trans-31kmer v. 1.01; Illumina sequencing
13	78.3	MT153489.1	Hemipteran_chu-related_virus_OKIAV138	Trialeurodes vaporariorum	Arthropoda	Insecta	Hemiptera	Aleyrodidae	Germany	SOAPdenovo-Trans-31kmer v. 1.01; Illumina sequencing
13	79	MT153369.1	Hemipteran_chu-related_virus_OKIAV139	Acanthocasuarina muellerianae	Arthropoda	Insecta	Hemiptera	Triozidae	Australia	SOAPdenovo-Trans-31kmer v. 1.01; Illumina sequencing
13	80.1	MW288229.1	Hymenopteran_chu-related_virus_OKIAV146	Trichopria drosophilae	Arthropoda	Insecta	Hymenoptera	Diapriidae	France	SOAPdenovo-Trans-31kmer v. 1.01; Illumina sequencing
13	80.2	MW288211.1	Hymenopteran_chu-related_virus_OKIAV146	Trichopria drosophilae	Arthropoda	Insecta	Hymenoptera	Diapriidae	France	SOAPdenovo-Trans-31kmer v. 1.01; Illumina sequencing
13	80.3	MW288173.1	Hymenopteran_chu-related_virus_OKIAV146	Trichopria drosophilae	Arthropoda	Insecta	Hymenoptera	Diapriidae	France	SOAPdenovo-Trans-31kmer v. 1.01; Illumina sequencing
13	81.1	MT153533.1	Coleopteran_chu-related_virus_OKIAV151	Larinus minutus	Arthropoda	Insecta	Coleoptera	Curculionidae	USA	SOAPdenovo-Trans-31kmer v. 1.01; Illumina sequencing
13	81.2	MT153523.1	Coleopteran_chu-related_virus_OKIAV151	Larinus minutus	Arthropoda	Insecta	Coleoptera	Curculionidae	USA	SOAPdenovo-Trans-31kmer v. 1.01; Illumina sequencing
13	82	MT153480.1	Phasmatodean_chu-related_virus_OKIAV118	Abrosoma johorensis	Arthropoda	Insecta	Phasmatodea	Phasmatidae	Germany	SOAPdenovo-Trans-31kmer v. 1.01; Illumina sequencing
13	83	MW288231.1	Hymenopteran_chu-related_virus_OKIAV122	Chelostoma florisomne	Arthropoda	Insecta	Hymenoptera	Megachilidae	Germany	SOAPdenovo-Trans-31kmer v. 1.01; Illumina sequencing
13	84	MT153518.1	Grylloblattodean_chu-related_virus_OKIAV120	Grylloblatta bifratrilecta	Arthropoda	Insecta	Notoptera	Grylloblattidae	USA	SOAPdenovo-Trans-31kmer v. 1.01; Illumina sequencing
13	85.1	MW288235.1	Orthopteran_chu-related_virus_OKIAV152	Phymateus viridipes	Arthropoda	Insecta	Orthoptera	Pyrgomorphidae	South Africa	SOAPdenovo-Trans-31kmer v. 1.01; Illumina sequencing

paper_id	virus_id	virus_access	virus_name	host	host_phylum	host_class	host_order	host_family	country	methods
13	85.2	MW288178.1	Orthopteran_chu-related_virus_OKIAV152	Phymateus viridipes	Arthropoda	Insecta	Orthoptera	Pyrgomorphidae	South Africa	SOAPdenovo-Trans-31kmer v. 1.01; Illumina sequencing
13	86.1	MW039258.1	Hymenopteran_chu-related_virus_OKIAV147	Aphelinus abdominalis	Arthropoda	Insecta	Hymenoptera	Aphelinidae	Lab culture of unknown geographical origin	SOAPdenovo-Trans-31kmer v. 1.01; Illumina sequencing
13	86.2	MW039257.1	Hymenopteran_chu-related_virus_OKIAV147	Aphelinus abdominalis	Arthropoda	Insecta	Hymenoptera	Aphelinidae	Lab culture of unknown geographical origin	SOAPdenovo-Trans-31kmer v. 1.01; Illumina sequencing
13	87	MT153495.1	Phasmatodean_chu-related_virus_OKIAV1344	Orthomeria sp.	Arthropoda	Insecta	Phasmatodea	Aschiphasmatidae	Germany	SOAPdenovo-Trans-31kmer v. 1.01; Illumina sequencing
13	88	MW039255.1	Hymenopteran_chu-related_virus_OKIAV125	Megachile willughbiella	Arthropoda	Insecta	Hymenoptera	Megachilidae	Germany	SOAPdenovo-Trans-31kmer v. 1.01; Illumina sequencing
13	89.1	MT153506.1	Dermapteran_chu-related_virus_OKIAV142	Gonolabis marginalis	Arthropoda	Insecta	Dermaptera	Anisolabididae	Japan	SOAPdenovo-Trans-31kmer v. 1.01; Illumina sequencing
13	89.2	MT153494.1	Dermapteran_chu-related_virus_OKIAV142	Gonolabis marginalis	Arthropoda	Insecta	Dermaptera	Anisolabididae	Japan	SOAPdenovo-Trans-31kmer v. 1.01; Illumina sequencing
13	90	MW288189.1	Hymenopteran_chu-related_virus_OKIAV124	Nomada lathburiana	Arthropoda	Insecta	Hymenoptera	Apidae	Germany	SOAPdenovo-Trans-31kmer v. 1.01
14	91	MH396473	Bat_chuvirus_isolate_KSA431	Taphozous perforatus	Chordata	Mammalia	Chiroptera	Emballonuridae	Saudi Arabia	Mira v. 4; Illumina sequencing
15	92.1	MT224150	Soybean_thrips_chu-like_virus_1,_Segment_L_	Neohydatothrips variabilis	Arthropoda	Insecta	Thysanoptera	Thripidae	USA	SPAdes v. 3.14.0; Illumina sequencing
15	92.2	MT293148	Soybean_thrips_chu-like_virus_1,_Segment_S__	Neohydatothrips variabilis	Arthropoda	Insecta	Thysanoptera	Thripidae	USA	SPAdes v. 3.14.0; Illumina sequencing
15	93	MT293147	Soybean_thrips_chu-like_virus_2_	Neohydatothrips variabilis	Arthropoda	Insecta	Thysanoptera	Thripidae	USA	SPAdes v. 3.14.0; Illumina sequencing
15	94	MT293151	Soybean_thrips_chu-like_virus_3	Neohydatothrips variabilis	Arthropoda	Insecta	Thysanoptera	Thripidae	USA	SPAdes v. 3.14.0; Illumina sequencing

paper_id	virus_id	virus_access	virus_name	host	host_phylum	host_class	host_order	host_family	country	methods
15	95	MW033643	Soybean_thrips_chu-like_virus_4	Neohydatothrips variabilis	Arthropoda	Insecta	Thysanoptera	Thripidae	USA	SPAdes v. 3.14.0; Illumina sequencing
15	96	MW033644	Soybean_thrips_chu-like_virus_5	Neohydatothrips variabilis	Arthropoda	Insecta	Thysanoptera	Thripidae	USA	SPAdes v. 3.14.0; Illumina sequencing
15	97	MW033645	Soybean_thrips_chu-like_virus_6	Neohydatothrips variabilis	Arthropoda	Insecta	Thysanoptera	Thripidae	USA	SPAdes v. 3.14.0; Illumina sequencing
15	98	MW033646	Soybean_thrips_chu-like_virus_7	Neohydatothrips variabilis	Arthropoda	Insecta	Thysanoptera	Thripidae	USA	SPAdes v. 3.14.0; Illumina sequencing
15	99	MW033647	Soybean_thrips_chu-like_virus_8	Neohydatothrips variabilis	Arthropoda	Insecta	Thysanoptera	Thripidae	USA	SPAdes v. 3.14.0; Illumina sequencing
16	100	MN599998.1	Karukera_tick_virus	Rhipicephalus microplus and Amblyomma variegatum	Arthropoda	Arachnida	Ixodida	Ixodidae	Guadeloupe : Martinique	CLC Genomics package v. 2017; Illumina sequencing
16	101	MN599999.1	Wuhan_tick_virus_2	Rhipicephalus microplus and Amblyomma variegatum	Arthropoda	Arachnida	Ixodida	Ixodidae	Guadeloupe : Martinique	CLC Genomics package v. 2017; Illumina sequencing
17	102	MN803434	Schistocephalus_solidus_jingchuvirus	Schistocephalus solidus	Platyhelminthes	Cestoda	Diphyllobothriidea	diphyllobothriidae	USA	SPAdes v. 3.11.1; Geneious v. R7; Illumina sequencing; Sanger dideoxy sequencing
18	103	MN567051	Herr_Frank_virus-1	Boa constrictor	Chordata	Reptilia	Squamata	Boidae	Brazil	MIRA v. 4.9.5; Illumina sequencing; Sanger dideoxy sequencing
19	104	KX924630.1	Chuvirus_Mos8Chu0	Culiseta minnesotae	Arthropoda	Insecta	Diptera	Culicidae	USA	SPAdes v. 3.8.0; Illumina sequencing
19	105.1	MH620818.1	Lampyris_noctiluca_chuvirus-like_virus_1	Lampyris noctiluca	Arthropoda	Insecta	Coleoptera	lampyridae	Finland	Trinity v. 2.6.5; CAP3 v. 3; NOVOplasty v. 2.7.1; Illumina sequencing
19	105.2	MH620819.1	Lampyris_noctiluca_chuvirus-like_virus_1	Lampyris noctiluca	Arthropoda	Insecta	Coleoptera	lampyridae	Finland	Trinity v. 2.6.5; CAP3 v. 3; NOVOplasty v. 2.7.1; Illumina sequencing

paper_id	virus_id	virus_access	virus_name	host	host_phylum	host_class	host_order	host_family	country	methods
20	106	MG600009.1	Guangdong_snake_chuvirus-like_virus	Lycodon rufozonatus	Chordata	Lepidosauria	Squamata	Colubridae	China	Illumina sequencing, Trinity v.2.1
20	107	MG600011.1	Wenling_fish_chuvirus-like_liver	Hoplichthys sp.	Chordata	Actinopterygii	Perciformes	Hoplichthyidae	China	Illumina sequencing, Trinity v.2.1
20	108	MG600010.1	Wenling_fish_chuvirus-like_gill	Hoplichthys sp.	Chordata	Actinopterygii	Perciformes	Hoplichthyidae	China	Illumina sequencing, Trinity v.2.1
21	109	MN190034.1	Hancheng_Leafhopper_Mivirus	Psammotettix alienus	Arthropoda	Insecta	Hemiptera	Cicadellidae	China	Illumina sequencing, Velvet v. 1.2.10
