# Supplementary material for: Ancient origin of Jingchuvirales derived glycoproteins integrated in arthropod genomes
Source: Genet Mol Biol. 2023 Apr 7;46(1):e20220218. doi: 10.1590/1678-4685-GMB-2022-0218 (PMC10084718; doi:10.1590/1678-4685-GMB-2022-0218)
Supplement: Table S3 - [file 1415-4757-GMB-46-1-e20220218-s3.pdf]

Genetics and Molecular Biology

https://doi.org/10.1590/1678-4685-GMB-2022-0218

Supplementary Material to "Ancient origin of Jingchuvirales derived glycoproteins integrated in arthropod genomes"

Table S3 - Viruses included in this study

virus_id	virus_access	genome_length	structure	segments	gene_order	blast	gene_order_curation	complete
1	NC_028259	11183	circular	1	L-G-N	-	L-G-N	TRUE
2	NC_028260	10877	circular	1	L-G-N	-	L-G-N	TRUE
3	NC_028261	9527	circular	1	L-N	-	L-N	FALSE
04.01	KM817597.1	7051	circular	2	L	-	L	TRUE
04.02	KM817596.1	4426	circular	2	G-N	-	G-N	
5	NC_031214	13732	linear	1	G-N-L	-	G-N-L	TRUE
6	KM817612	5647	linear	1	L	-	L	FALSE
7	KM817614	8658	linear	1	L	-	L	FALSE
8	KR902734.1	18554	linear	1	POL	-	POL	FALSE
9	NC_028263.1	10815	circular	1	G-N-L	-	G-N-L	TRUE
10	NC_028264.1	10017	circular	1	L-N	-	L-N	FALSE
11.1	KM817601.1	6959	circular	2	L	-	L	TRUE
11.2	KM817602.1	4698	circular	2	G-N	-	G-N	
12	NC_031248.1	12860	linear	1	G1-N-G2-L	-	G1-N-G2-L	TRUE
13.1	NC_043472.1	7152	circular	2	L	-	L	TRUE
13.2	NC_043473.1	4788	circular	2	G-N	-	G-N	
14.1	NC_043476.1	6777	circular	2	L	-	L	FALSE
14.2	NC_043475	2291	circular	2	N	-	N	
15.1	NC_043471.1	6808	circular	2	L	-	L	FALSE
15.2	NC_043470.1	2343	circular	2	N	-	N	
16	NC_028265.1	12004	circular	1	L-G-N	-	L-G-N	TRUE
17	NC_028266	11395	circular	1	L-G-N	-	L-G-N	TRUE

virus_id	virus_access	genome_length	structure	segments	gene_order	blast	gene_order_curation	complete
18.1	MT293148	6783	linear	2	L	-	L	TRUE
18.2	KX884425	3958	linear	2	G-HP	QPL15354.1	G-N	
19	KX884427	11280	p-circular	1	L-G-HP	QMP82322.1	L-G-N	TRUE
20	KX884438	5150	linear	1	L	-	L	FALSE
21	KX884449	6589	linear	1	L	-	L	FALSE
22	KX884455	6877	linear	1	L	-	L	FALSE
23	KX884419	11349	linear	1	L-G-HP	QMP82322.1	L-G-N	TRUE
24	KX884439	11270	linear	1	L-G-HP	QHX39758.1/AVM87277.1/AVM87274.1/QUF61511.1/AJG39062.1	L-G-N	TRUE
25.1	KX884451	6699	linear	2	L	-	L	TRUE
25.2	KX884452	4615	linear	2	G-HP1-HP2	HP1:AJG39062.1/HP2:AJG39062.1	G-N-N	
26.1	KX884453	6746	linear	1	L	-	L	TRUE
26.2	KX884454	4083	linear	1	G-HP	AJG39062.1	G-N	
27	KX884409	11058	linear	1	G-HP-L	-	G-HP-L	FALSE
28	KX884458	11119	linear	1	L-G-HP	-	L-G-HP	FALSE
29	KX884461	7977	linear	1	HP-L	-	HP-L	FALSE
30	KX884444	13517	linear	1	G-HP1-HP2-L	HP1:QHA33912.1	G-N-HP-L	TRUE
31	KX884404	12996	linear	1	G-HP1-HP2-L	-	G-HP1-HP2-L	FALSE
32	KX884416	13741	linear	1	G-HP-L	QIH31160.1	G-N-L	TRUE
33	KU095839	11877	p-circular	1	L-G-N	-	L-G-N	TRUE
34	MF344589	705	linear	1	G	-	G	FALSE
35	MF344596	472	linear	1	G	-	G	FALSE
36	MF344588	558	linear	1	G	-	G	FALSE
37	MF344590	1353	linear	1	G	-	G	FALSE
38	MH155927	11208	p-circular	1	L-G-N	-	L-G-N	TRUE

virus_id	virus_access	genome_length	structure	segments	gene_order	blast	gene_order_curation	complete
39.1	MH155926	1077	p-circular	3	L	-	L	TRUE
39.2	MH155925	993	p-circular	3	N	-	N	
39.3	MH155924	747	p-circular	3	G	-	G	
40	MH155923	11231	p-circular	1	L-G-N	-	L-G-N	TRUE
41	MH155922	924	p-circular	1	L	-	L	FALSE
42	MH155921	10910	p-circular	1	L-G-N	-	L-G-N	TRUE
43	MH155920	10658	p-circular	1	L-G-N	-	L-G-N	TRUE
44	KM817599	10815	circular	1	G-N-L	-	G-N-L	TRUE
45	KM817593	11183	circular	1	L-G-N	-	L-G-N	TRUE
46	KM817594	10877	circular	1	L-G-N	-	L-G-N	TRUE
47	KM817603	12860	linear	1	G-N-G-L	-	G-N-G-L	TRUE
48.1	KM817604	7152	circular	2	L	-	L	TRUE
48.2	KM817605	4788	circular	2	G-N	-	G-N	
49.1	KM817595	9527	circular	1	L-N	-	L-N	FALSE
49.2	KM817600	10017	circular	1	L-N	-	L-N	FALSE
50.1	KM817609	6808	circular	2	L	-	L	FALSE
50.2	KM817608	2343	circular	2	N	-	N	
51	KM817613	6714	linear	1	L	-	L	FALSE
52	KU230451	11163	circular	1	L-G-ORF3-ORF4	ORF3:AYV61049.1	L-G-N-ORF4	TRUE
53.1	KM817606	6777	circular	2	L	-	L	FALSE
53.2	KM817607	2291	circular	2	N	-	N	
54	KM817610	12004	circular	1	L-G-N	-	L-G-N	TRUE
55	KM817598	13732	linear	1	G-N-L	-	G-N-L	TRUE
56	KM460042	10795	circular	1	L-G-ORF3-ORF4	ORF3:YP_009177706.1/QFR36196.1	L-G-N-ORF4	TRUE
57	KM817611	11395	circular	1	L-G-N	-	L-G-N	TRUE

virus_id	virus_access	genome_length	structure	segments	gene_order	blast	gene_order_curation	complete
58	NC028243	10795	circular	1	L-G-ORF3-ORF4	ORF3:YP_009177706.1/QFR36196.1	L-G-N-ORF4	TRUE
59	MF360789	11518	circular	1	L-G-ORF3-ORF4	ORF3: YP_009666258.1/QMP82283.1	L-G-N-ORF4	TRUE
60	MF893252	10333	linear	1	HP1-HP2-HP3-HP4-HP5	HP1:YP_009300661.1/HP3:YP_009300662.1/HP5: YP_009300663.1	G-HP2-N-HP4-L	TRUE
61	MF893248	3744	linear	1	HP	QDB75014.1	L	FALSE
62	MK026566.1	11192	p-circular	1	L-N-G-HP	AYV61054.1	L-N-G-L	TRUE
63	MK026591.1	11018	p-circular	1	L-G-N	-	L-G-N	TRUE
64	MN025520.1	11272	circular	1	L-G-N-Unk	-	L-G-N-Unk	TRUE
65	MN025521.1	11187	circular	1	L-G-N-Unk	-	L-G-N-Unk	TRUE
66	MK780200	864	linear	1	G	-	G	FALSE
67	MN095546	11392	circular	1	L-G-N	-	L-G-N	TRUE
68	MN095545	10031	circular	1	N-L-G	-	N-L-G	TRUE
69.1	MT153403.1	1852	linear	2	L	-	L	FALSE
69.2	MT153467.1	633	linear	2	G	-	G	
70	MW288217.1	1856	linear	1	L	-	L	FALSE
71	MT153458.1	2218	linear	1	L	-	L	FALSE
72	MT153417.1	2382	linear	1	L	-	L	FALSE
73	MW039261.1	12047	linear	1	G-N-L	-	G-N-L	TRUE
74	MW039254.1	14106	linear	1	G-N-L	-	G-N-L	TRUE
75	MW039256.1	14386	linear	1	G-N-L	-	G-N-L	TRUE
76.1	MW288199.1	1999	linear	2	N-HP	-	N-HP	FALSE
76.2	MW288213.1	2548	linear	2	L	-	L	
77.1	MW288180.1	2721	linear	2	L	-	L	TRUE
77.2	MW288234.1	4272	linear	2	G-N	-	G-N	

virus_id	virus_access	genome_length	structure	segments	gene_order	blast	gene_order_curation	complete
78.1	MT153371.1	3324	linear	2	L	-	L	TRUE
78.2	MT153353.1	2837	linear	2	HP-N	-	HP-N	
78.3	MT153489.1	716	linear	2	G	-	G	
79	MT153369.1	3325	linear	1	L	-	L	FALSE
80.1	MW288229.1	3299	linear	3	G-HP	-	G-HP	TRUE
80.2	MW288211.1	2592	linear	3	N-HP	-	N-HP	
80.3	MW288173.1	5210	linear	3	L	-	L	
81.1	MT153533.1	5478	linear	2	L	-	L	TRUE
81.2	MT153523.1	4395	linear	2	G-N-HP	-	G-N-HP	
82	MT153480.1	5657	linear	1	L	-	L	FALSE
83	MW288231.1	5882	linear	1	N-L	-	N-L	FALSE
84	MT153518.1	5993	linear	1	L	-	L	FALSE
85.1	MW288235.1	2649	linear	2	G	-	G	FALSE
85.2	MW288178.1	6828	linear	2	L	-	L	
86.1	MW039258.1	4519	linear	2	N-G	-	N-G	TRUE
86.2	MW039257.1	7082	linear	2	L	-	L	
87	MT153495.1	1363	linear	1	L	-	L	FALSE
88	MW039255.1	7530	linear	1	L	-	L	FALSE
89.1	MT153506.1	8463	linear	2	L-G	-	L-G	TRUE
89.2	MT153494.1	2182	linear	2	N-HP	-	N-HP	
90	MW288189.1	13339	linear	1	G-N-L	-	G-N-L	TRUE
91	MH396473	762	linear	1	L	-	L	FALSE
92.1	MT224150	7386	p-circular	2	L	-	L	FALSE
92.2	MT293148	2263	p-circular	2	HP-G	-	HP-G	
93	MT293147	4327	linear	1	G-N	-	G-N	FALSE
94	MT293151	2020	linear	1	HP-G	-	HP-G	FALSE
95	MW033643	2603	linear	1	G	-	G	FALSE
96	MW033644	2369	linear	1	HP-G	-	HP-G	FALSE
97	MW033645	1503	linear	1	G	-	G	FALSE
98	MW033646	1275	linear	1	HP-G	-	HP-G	FALSE

virus_id	virus_access	genome_length	structure	segments	gene_order	blast	gene_order_curation	complete
99	MW033647	1755	linear	1	G	-	G	FALSE
100	MN599998.1	11177	circular	1	L-G-N	-	L-G-N	TRUE
101	MN599999.1	11393	circular	1	L-G-N	-	L-G-N	TRUE
102	MN803434	6863	circular	1	L	-	L	FALSE
103	MN567051	10718	linear	1	N-G-L	-	N-G-L	TRUE
104	KX924630.1	6844	linear	1	L	-	L	FALSE
105.1	MH620818.1	6829	linear	2	L	-	L	TRUE
105.2	MH620819.1	4489	linear	2	G-N-HP	-	G-N-HP	
106	MG600009.1	10625	linear	1	L-G-N	-	L-G-N	TRUE
107	MG600011.1	10407	linear	1	L-G-N	-	L-G-N	TRUE
108	MG600010.1	10385	linear	1	L-G-N	-	L-G-N	TRUE
109	MN190034.1	9852	linear	1	N-L	-	L-N	FALSE
