# Supplementary material for: Ancient origin of Jingchuvirales derived glycoproteins integrated in arthropod genomes
Source: Genet Mol Biol. 2023 Apr 7;46(1):e20220218. doi: 10.1590/1678-4685-GMB-2022-0218 (PMC10084718; doi:10.1590/1678-4685-GMB-2022-0218)
Supplement: Table S4 - [file 1415-4757-GMB-46-1-e20220218-s4.pdf]

Genetics and Molecular Biology
https://doi.org/10.1590/1678-4685-GMB-2022-0218

Supplementary Material to "Ancient origin of Jingchuvirales derived glycoproteins integrated in arthropod genomes"

Table S4 - Host genomes and EVEs copies.

Assembly	species	EVEs	Total Copies	Total copies with TEs
GCF_000204515.1	Acromyrmex echinatior	1	9	7
GCF_005508785.1	Acyrthosiphon pisum	2	6	5
GCF_013141755.1	Anopheles stephensi	2	11	11
GCA_009835225.1	Aphis craccivora	4	8	1
GCA_009761285.1	Aphis glycines	1	2	2
GCF_004010815.1	Aphis gossypii	1	1	1
GCF_001854935.1	Bemisia tabaci	1	9	8
GCF_011952205.1	Bombus bifarius	2	43	36
GCF_011952275.1	Bombus vancouverensis nearcticus	11	71	60
GCF_003227725.1	Camponotus floridanus	1	103	80
GCF_000341935.1	Cephus cinctus	2	7	7
GCA_902439185.1	Cinara cedri	1	5	2
GCF_016801865.1	Culex pipiens pallens	2	13	13
GCF_001594065.1	Cyphomyrmex costatus	19	29	25
GCF_001412515.2	Diachasma alloeum	2	43	34
GCF_001483705.1	Eufriesea mexicana	1	6	5
GCF_001263275.1	Habropoda laboriosa	12	17	10
GCF_003227715.1	Harpegnathos saltator	8	120	114
GCA_900096555.1	Heligmosomoides polygyrus bakeri	2	2	1
GCF_000764305.1	Hyalella azteca	1	1	0
GCA_001045655.1	Lasius niger	9	14	7
GCF_000500325.1	Leptinotarsa decemlineata	1	3	2
GCF_013373865.1	Monomorium pharaonis	4	42	38

Assembly	species	EVEs	Total Copies	Total copies with TEs
GCF_001856785.1	Myzus persicae	2	4	2
GCF_009193385.2	Nasonia vitripennis	1	36	34
GCF_010583005.1	Odontomachus brunneus	5	71	46
GCA_003672135.1	Ooceraea biroi	16	55	38
GCA_012274295.1	Osmia lignaria	1	19	18
GCF_008802855.1	Photinus pyralis	1	1	1
GCF_003676215.2	Rhopalosiphum maidis	5	20	19
GCF_016802725.1	Solenopsis invicta	5	68	66
GCF_001594075.1	Trachymyrmex cornetzi	10	23	20
GCF_001594115.1	Trachymyrmex septentrionalis	3	6	5
GCF_001594055.1	Trachymyrmex zeteki	6	11	11
GCF_000002335.3	Tribolium castaneum	3	4	2
GCA_902806795.1	Trichogramma brassicae	6	22	18
GCA_002249905.1	Trichomalopsis sarcophagae	3	8	2
GCF_014083535.2	Vespa mandarinia	1	26	25
