# Supplementary material for: Ancient origin of Jingchuvirales derived glycoproteins integrated in arthropod genomes
Source: Genet Mol Biol. 2023 Apr 7;46(1):e20220218. doi: 10.1590/1678-4685-GMB-2022-0218 (PMC10084718; doi:10.1590/1678-4685-GMB-2022-0218)
Supplement: Table S5 - [file 1415-4757-GMB-46-1-e20220218-s5.pdf]

Genetics and Molecular Biology
https://doi.org/10.1590/1678-4685-GMB-2022-0218

Supplementary Material to "Ancient origin of Jingchuvirales derived glycoproteins integrated in arthropod genomes"

Table S5 - Endogenous viruses flanking regions structures.

Sequence	LTR5	LTR3	TSR	PPT	pos_gag	pos_protease	pos_rnase	pos_rt	pos_integrase	pos_transposase	complete	TE	Structure
acromyrmex_echinatior_NW_011627030.1:469725-490867	NA	NA	NA	NA	NA	NA	NA	NA	15688-15206	NA	FALSE		
acromyrmex_echinatior_NW_011627053.1:53271-74203	NA	NA	NA	NA	NA	NA	7259-7645	7259-7645	NA	NA	FALSE		
acromyrmex_echinatior_NW_011627112.1:209919-231037	NA	NA	NA	NA	NA	19025-19552, 14941-9185	14941-9185	14941-9185	14941-9185	14941-9185	FALSE		
acromyrmex_echinatior_NW_011627123.1:3723-24682	NA	NA	NA	NA	NA	NA	1913-2218	1913-2218	3428-3814	NA	FALSE		
acromyrmex_echinatior_NW_011627221.1:23513-44472	NA	NA	NA	NA	NA	NA	5869-6198	5869-6198	NA	NA	FALSE		
acromyrmex_echinatior_NW_011627320.1:236047-257090	NA	NA	NA	NA	NA	NA	NA	6952-7395	5991-4855	5991-4855	FALSE		
acromyrmex_echinatior_NW_011627401.1:58551-79537	NA	NA	NA	NA	NA	NA	13602-13288, 13130-12726	14323-13991, 13602-13288	NA	NA	FALSE		
acyrthosiphon_pisum_NC_042494.1:149706572-149728452	NA	NA	NA	NA	NA	NA	NA	NA	NA	20729-20382	FALSE		
acyrthosiphon_pisum_NC_042495.1:22174253-22195647	NA	NA	NA	NA	NA	NA	7539-9914	7539-9914	7539-9914	NA	FALSE		
acyrthosiphon_pisum_NC_042495.1:22191452-22213272	NA	NA	NA	NA	NA	5469-5984	7875-9941	7355-7888	7875-9941	NA	FALSE		
acyrthosiphon_pisum_NW_021771311.1:84163-105947	NA	NA	NA	NA	17650-16313	16283-15135	14215-11891	15220-14228, 14215-11891	14215-11891	NA	FALSE		
acyrthosiphon_pisum_NW_021771402.1:1-16965	NA	NA	NA	NA	NA	NA	NA	2807-3211	NA	NA	FALSE		
anopheles_stephensi_NC_050201.1:15748001-15769794	NA	NA	NA	NA	NA	6244-7164	NA	NA	NA	2198-2539, 7577-8137	FALSE		
anopheles_stephensi_NC_050201.1:16357814-16379826	NA	NA	NA	NA	4499-7393	7240-9465	NA	4499-7393, 20853-20260	7240-9465	NA	FALSE		
anopheles_stephensi_NC_050201.1:16377136-16398407	NA	NA	NA	NA	NA	NA	17881-14909	17881-14909, 6154-5435, 1531-938	9257-8892	9058-8546	FALSE		
anopheles_stephensi_NC_050201.1:16521872-16543884	NA	NA	NA	NA	14851-10001	NA	NA	NA	NA	NA	FALSE		
anopheles_stephensi_NC_050201.1:19895175-19917043	NA	NA	NA	NA	15968-15627, 15623-15312	2055-2369, 7094-11899, 21848-19416, 13853-13317	2388-5174, 17625-16714	2388-5174, 7094-11899, 21848-19416, 18037-17603, 17625-16714	2388-5174, 7094-11899, 19409-18666	NA	FALSE		
anopheles_stephensi_NC_050202.1:82482790-82504802	NA	NA	NA	NA	14851-10001	NA	NA	NA	NA	NA	FALSE		
anopheles_stephensi_NC_050203.1:46573021-46594763	NA	NA	NA	NA	15506-14682	14540-14226	NA	NA	13802-13233	NA	FALSE		
anopheles_stephensi_NC_050203.1:77813456-77835468	NA	NA	NA	NA	7163-12013	NA	NA	NA	NA	NA	FALSE		
anopheles_stephensi_NW_023404986.1:42165-63907	NA	NA	NA	NA	4-1068, 14277-13837	NA	NA	3694-5574	NA	NA	FALSE		
anopheles_stephensi_NW_023405001.1:1-15558	NA	NA	NA	NA	708-5558, 8630-6648	NA	NA	8630-6648	NA	8630-6648	FALSE		
anopheles_stephensi_NW_023405363.1:19663-41591	3564:4401	11988:12823	3559:3563/12824:12828	11973:11987	5099-10621	5099-10621	14634-17003	17-742, 436-1329, 5099-10621, 14634-17003	5099-10621, 17003-17740	NA	TRUE	Bel/Pao	LTR-[GAG-PR-RT-RH-INT]-ENV-LTR
aphis_craccivora_VUJU01003749.1:1-20969	11:352	11924:12262	7:10/12263:12266	11824:11838	NA	NA	NA	NA	NA	NA	FALSE		
aphis_glycines_VYZN01000008.1:2582231-2603742	NA	NA	NA	NA	NA	NA	NA	NA	NA	13145-15073, 19759-20265, 3600-2788	FALSE		
aphis_glycines_VYZN01003027.1:13334-34851	NA	NA	NA	NA	NA	2-2623	5208-8009	2-2623, 5208-8009	2-2623, 7975-8511	NA	FALSE		
aphis_gossypii_NW_021006118.1:1222-23162	NA	NA	NA	NA	17887-16604	16634-12252	16634-12252	16634-12252	16634-12252	16634-12252	FALSE		
bemisia_tabaci_NW_017547116.1:2545645-2567591	NA	NA	NA	NA	18781-16667	16502-13242	16502-13242	16502-13242	16502-13242	NA	FALSE		
bemisia_tabaci_NW_017547118.1:1876453-1898399	NA	NA	NA	NA	3171-5282	5513-9460	5513-9460	5513-9460	5513-9460	5513-9460	FALSE		
bemisia_tabaci_NW_017547204.1:782040-803803	7275:7814	18994:19534	7271:7274/19535:19538	18941:18955	18243-16210	16246-12182	16246-12182	16246-12182	16246-12182	16246-12182	TRUE	Ty3/Gypsy	LTR-EXTENSIN-ENV-[INT-

Sequence	LTR5	LTR3	TSR	PPT	pos_gag	pos_protease	pos_rnase	pos_rt	pos_integrase	pos_transposase	complete	TE	Structure
													RH-RT-PR-GAG]-GAG-LTR
bemisia_tabaci_NW_017547602.1:14156-35919	NA	NA	NA	NA	NA	16242-12178	16242-12178	16242-12178	16242-12178	16242-12178	FALSE		
bemisia_tabaci_NW_017547915.1:1896286-1918223	NA	NA	NA	NA	19296-17059	17083-12719	17083-12719	17083-12719	17083-12719	17083-12719	FALSE		
bemisia_tabaci_NW_017548708.1:2458834-2480771	NA	NA	NA	NA	NA	NA	NA	16576-15887	NA	NA	FALSE		
bemisia_tabaci_NW_017557114.1:1-16994	NA	NA	NA	NA	NA	3726-7853	3726-7853	3726-7853	3726-7853	3726-7853	FALSE		
bemisia_tabaci_NW_017564154.1:1028221-1050095	7039:7260	12399:12620	7034:7038/12621:12625	7305:7319	NA	NA	NA	NA	NA	NA	FALSE		
bombus_bifarius_NW_022884553.1:1-14469	NA	NA	NA	NA	NA	NA	652-2	1614-688, 652-2	NA	NA	FALSE		
bombus_bifarius_NW_022884557.1:1-11513	NA	NA	NA	NA	NA	NA	10648-8378, 6291-4171	11094-10606, 10648-8378	10648-8378, 6291-4171	10648-8378	FALSE		
bombus_bifarius_NW_022884559.1:1-11488	NA	NA	NA	NA	NA	NA	10653-8383, 6296-4176	11372-10611, 10653-8383	10653-8383, 6296-4176	10653-8383	FALSE		
bombus_bifarius_NW_022884564.1:7354-22960	NA	NA	NA	NA	NA	NA	15473-13377	NA	15473-13377	NA	FALSE		
bombus_bifarius_NW_022884582.1:1-14523	NA	NA	NA	NA	NA	3765-1	3765-1	3765-1	14423-13068, 3765-1	NA	FALSE		
bombus_bifarius_NW_022884599.1:75231-96088	NA	NA	NA	NA	NA	NA	3927-7067	3927-7067	3927-7067	NA	FALSE		
bombus_bifarius_NW_022884610.1:1-13567	NA	NA	NA	NA	NA	5359-1259	5359-1259	5359-1259, 438-1	13557-12442, 5359-1259	12442-11846	FALSE		
bombus_bifarius_NW_022884641.1:271486-292208	NA	NA	NA	NA	NA	NA	NA	15838-14468	NA	NA	FALSE		
bombus_bifarius_NW_022884655.1:1-17956	8134:8566	11283:11715	8128:8133/11716:11721	8600:8614	3783-2830	11139-8635	16329-14209, 11139-8635	NA	16329-14209, 11139-8635	NA	FALSE		
bombus_bifarius_NW_022884841.1:1-11958	6320:7131	10120:10931	6314:6319/10932:10937	10093:10107	NA	7075-8628	NA	NA	8597-9469	NA	FALSE		
bombus_bifarius_NW_022884848.1:1-14851	NA	NA	NA	NA	NA	NA	NA	9841-8624	NA	NA	FALSE		
bombus_bifarius_NW_022885224.1:1-15979	NA	NA	NA	NA	NA	NA	15825-14473, 14275-13079	104-472, 15825-14473	14275-13079, 13009-12155	NA	FALSE		
bombus_bifarius_NW_022885323.1:1-11880	NA	NA	NA	NA	NA	NA	6670-4589	NA	6670-4589	NA	FALSE		
bombus_bifarius_NW_022885338.1:5300-23745	NA	NA	NA	NA	NA	NA	15632-13512	NA	15632-13512	18393-17932	FALSE		
bombus_bifarius_NW_022885339.1:1-13105	NA	NA	NA	NA	NA	NA	13075-11444, 11456-9963, 7876-5756	13075-11444	11456-9963, 7876-5756	11456-9963	FALSE		
bombus_bifarius_NW_022885344.1:2438-16930	NA	NA	NA	NA	NA	5887-1787	5887-1787	5887-1787, 1454-336	14440-13541, 5887-1787	NA	FALSE		
bombus_bifarius_NW_022885345.1:1-14185	NA	NA	NA	NA	NA	3893-3	13634-11862, 3893-3	3893-3	13634-11862, 3893-3	NA	FALSE		
bombus_bifarius_NW_022885346.1:1-11907	NA	NA	NA	NA	NA	NA	6713-4593	NA	6713-4593	NA	FALSE		
bombus_bifarius_NW_022885365.1:19196-40053	NA	NA	NA	NA	NA	NA	NA	6020-7237	NA	NA	FALSE		
bombus_bifarius_NW_022885401.1:6287-19742	NA	NA	NA	NA	NA	2750-5002	2750-5002, 5197-7317	2750-5002	5197-7317	NA	FALSE		
bombus_bifarius_NW_022885402.1:1-15102	NA	NA	NA	NA	NA	9513-13601	9513-13601	9513-13601, 13934-15052	223-1365, 9513-13601	NA	FALSE		
bombus_bifarius_NW_022885403.1:1-14034	NA	NA	NA	NA	NA	8449-11559	8449-11559	8449-11559, 12875-13993	8449-11559, 11526-12542	NA	FALSE		
bombus_bifarius_NW_022885405.1:1-18469	NA	NA	NA	NA	NA	17462-15285	17462-15285, 14305-12185	17462-15285	14305-12185	NA	FALSE		
bombus_bifarius_NW_022885409.1:784-15474	NA	NA	NA	NA	NA	NA	NA	NA	14648-14268	14095-13538	FALSE		
bombus_bifarius_NW_022885410.1:1-14875	NA	NA	NA	NA	NA	NA	14843-11730, 9643-8123	14843-11730	14843-11730, 9643-8123	14843-11730, 8123-7779	FALSE		
bombus_bifarius_NW_022885411.1:1-12765	NA	NA	NA	NA	NA	NA	12736-9623, 7536-5416	12736-9623	12736-9623, 7536-5416	12736-9623	FALSE		
bombus_bifarius_NW_022885418.1:697-14149	NA	NA	NA	NA	NA	NA	5092-3152	2819-1701	5092-3152	NA	FALSE		
bombus_bifarius_NW_022885439.1:1-13926	NA	NA	NA	NA	NA	13025-10746	13025-10746, 10551-8431	13025-10746	10551-8431	NA	FALSE		
bombus_bifarius_NW_022885454.1:4926-25783	NA	NA	NA	NA	NA	18144-17179	16193-14031	17204-16800, 16848-16162	16193-14031	16193-14031	FALSE		

Sequence	LTR5	LTR3	TSR	PPT	pos_gag	pos_protease	pos_rnase	pos_rt	pos_integrase	pos_transposase	complete	TE	Structure
bombus_bifarius_NW_022885480.1:1-12867	NA	NA	NA	NA	NA	NA	NA	6845-5628	NA	NA	FALSE		
bombus_bifarius_NW_022885480.1:3762-20552	NA	NA	NA	NA	NA	NA	NA	14836-13619	NA	NA	FALSE		
bombus_bifarius_NW_022885481.1:128-20985	NA	NA	NA	NA	NA	NA	NA	14836-13703	NA	NA	FALSE		
bombus_bifarius_NW_022885483.1:3823-21189	NA	NA	NA	NA	NA	NA	4240-5349	NA	5422-6135	NA	FALSE		
bombus_bifarius_NW_022885512.1:2706-16794	NA	NA	NA	NA	NA	NA	NA	1573-572	NA	NA	FALSE		
bombus_bifarius_NW_022885519.1:1-17853	NA	NA	NA	NA	NA	NA	137-1396, 1342-2766	137-1396, 12155-10938	1342-2766	NA	FALSE		
bombus_bifarius_NW_022885541.1:305-21162	NA	NA	NA	NA	NA	NA	15632-14523	NA	14450-13737	NA	FALSE		
bombus_vancouverensis_nearcticus_NW_022881837.1:2409838-2428486	NA	NA	NA	NA	NA	NA	16975-13937	16975-13937, 2437-1319	16975-13937	NA	FALSE		
bombus_vancouverensis_nearcticus_NW_022881867.1:409639-422058	NA	NA	NA	NA	NA	5182-1082	5182-1082	5182-1082	5182-1082	NA	FALSE		
bombus_vancouverensis_nearcticus_NW_022881884.1:207030-223380	NA	NA	NA	NA	NA	NA	16219-15872	5648-4530	15821-14106	15821-14106	FALSE		
bombus_vancouverensis_nearcticus_NW_022881947.1:9749-31125	NA	NA	NA	NA	NA	NA	21374-18261, 16174-15827	21374-18261, 5809-5216	21374-18261, 15776-14061	21374-18261, 15776-14061	FALSE		
bombus_vancouverensis_nearcticus_NW_022881977.1:1-14772	NA	NA	NA	NA	NA	NA	9167-9760	9167-9760	NA	27-623	FALSE		
bombus_vancouverensis_nearcticus_NW_022881993.1:1-17749	NA	NA	NA	NA	NA	13622-17716	3182-3772, 13622-17716	13622-17716	3934-4527, 13622-17716	250-1101, 4700-5296	FALSE		
bombus_vancouverensis_nearcticus_NW_022882008.1:43925-65301	NA	NA	NA	NA	NA	2997-3740	4028-7240	4028-7240	4028-7240	NA	FALSE		
bombus_vancouverensis_nearcticus_NW_022882008.1:44255-64767	NA	NA	NA	NA	NA	2667-3410	3698-6910	3698-6910	3698-6910	NA	FALSE		
bombus_vancouverensis_nearcticus_NW_022882137.1:1-20247	NA	NA	NA	NA	NA	19978-16571, 5715-3436	19978-16571, 14484-13258, 5715-3436	19978-16571, 5715-3436	19978-16571, 14484-13258, 13218-12364	19978-16571	FALSE		
bombus_vancouverensis_nearcticus_NW_022882137.1:16459-37892	NA	NA	NA	NA	NA	21167-17760, 6891-5809	21167-17760, 15672-14446, 5816-4611	21167-17760, 5816-4611	21167-17760, 15672-14446, 14406-13552	21167-17760	FALSE		
bombus_vancouverensis_nearcticus_NW_022882137.1:34102-45802	NA	NA	NA	NA	NA	6895-5813, 3524-117	5820-4615, 3524-117	5820-4615, 3524-117	3524-117	3524-117	FALSE		
bombus_vancouverensis_nearcticus_NW_022882160.1:24381-45637	3304:4115	9013:9824	3298:3303/9825:9830	4128:4142	NA	9069-4969	9069-4969	9069-4969	9069-4969	NA	FALSE		
bombus_vancouverensis_nearcticus_NW_022882180.1:1-12952	NA	NA	NA	NA	NA	NA	7256-7849	7256-7849	NA	NA	FALSE		
bombus_vancouverensis_nearcticus_NW_022882194.1:1-11680	NA	NA	NA	NA	NA	NA	NA	6291-5722	NA	NA	FALSE		
bombus_vancouverensis_nearcticus_NW_022882231.1:1-16829	NA	NA	NA	NA	NA	NA	NA	4110-6023	NA	NA	FALSE		
bombus_vancouverensis_nearcticus_NW_022882231.1:146-16829	NA	NA	NA	NA	NA	NA	NA	3965-5878	NA	NA	FALSE		
bombus_vancouverensis_nearcticus_NW_022882237.1:1-18716	10005:10816	15550:16360	10001:10004/16361:16364	15523:15537	NA	10760-14860	10760-14860	3906-4799, 10760-14860	10760-14860	NA	FALSE		
bombus_vancouverensis_nearcticus_NW_022882269.1:5423-24771	NA	NA	NA	NA	NA	2789-4132	4739-6967	2789-4132, 4084-4770, 17897-18790	4739-6967	NA	FALSE		
bombus_vancouverensis_nearcticus_NW_022882294.1:4813-25670	NA	NA	NA	NA	NA	NA	NA	15159-14266	NA	NA	FALSE		
bombus_vancouverensis_nearcticus_NW_022882295.1:1-16921	NA	NA	NA	NA	NA	NA	NA	11222-10530	NA	NA	FALSE		
bombus_vancouverensis_nearcticus_NW_022882296.1:1-19235	NA	NA	NA	NA	NA	NA	18579-17941	19184-18825, 13194-12301	NA	NA	FALSE		
bombus_vancouverensis_nearcticus_NW_022882367.1:14520-35092	NA	NA	NA	NA	NA	NA	20535-19723, 16193-14073	20535-19723, 3620-3096	19668-18265, 16193-14073	19668-18265	FALSE		
bombus_vancouverensis_nearcticus_NW_022882383.1:30237-42854	NA	NA	NA	NA	NA	NA	NA	2961-2596	NA	NA	FALSE		

Sequence	LTR5	LTR3	TSR	PPT	pos_gag	pos_protease	pos_rnase	pos_rt	pos_integrase	pos_transposase	complete	TE	Structure
bombus_vancouverensis_nearcticus_NW_022882384.1:14074-35507	NA	NA	NA	NA	NA	NA	21420-18307, 16220-15873	21420-18307, 3003-2638	21420-18307, 15822-14107	21420-18307, 15822-14107	FALSE		
bombus_vancouverensis_nearcticus_NW_022882401.1:44430-58075	NA	NA	NA	NA	NA	NA	NA	5706-6398	NA	NA	FALSE		
bombus_vancouverensis_nearcticus_NW_022882402.1:5086-25943	NA	NA	NA	NA	NA	NA	NA	5705-6922	16215-15175	NA	FALSE		
bombus_vancouverensis_nearcticus_NW_022882403.1:1-11042	NA	NA	NA	NA	NA	NA	NA	7297-7866	6312-5272	NA	FALSE		
bombus_vancouverensis_nearcticus_NW_022882403.1:1912-22448	NA	NA	NA	NA	NA	NA	NA	5386-5955	4401-3361	NA	FALSE		
bombus_vancouverensis_nearcticus_NW_022882514.1:51423-70215	NA	NA	NA	NA	NA	NA	34-3147, 5234-5581	34-3147	34-3147, 5632-7347	34-3147, 5632-7347	FALSE		
bombus_vancouverensis_nearcticus_NW_022882515.1:1-19735	NA	NA	NA	NA	NA	NA	379-981, 4124-4471	NA	4522-6237	1228-2037, 4522-6237	FALSE		
bombus_vancouverensis_nearcticus_NW_022882515.1:1-20326	NA	NA	NA	NA	NA	NA	379-981, 4124-4471	NA	4522-6237	1228-2037, 4522-6237	FALSE		
bombus_vancouverensis_nearcticus_NW_022882515.1:36476-51629	NA	NA	NA	NA	NA	6286-2192	6286-2192	6286-2192, 1859-741	15147-14044, 6286-2192	15147-14044	FALSE		
bombus_vancouverensis_nearcticus_NW_022882562.1:578629-600062	NA	NA	NA	NA	NA	2748-5027, 14107-17940	2748-5027, 5222-7342, 14107-17940	2748-5027, 14107-17940	5222-7342, 14107-17940	NA	FALSE		
bombus_vancouverensis_nearcticus_NW_022882574.1:1-18559	11791:12747	17442:18398	11785:11790/18399:18404	12760:12774	NA	17504-13428	17504-13428	2140-3549, 17504-13428	17504-13428	NA	FALSE		
bombus_vancouverensis_nearcticus_NW_022882580.1:1-12124	NA	NA	NA	NA	NA	NA	814-1161	NA	1212-2927	1212-2927	FALSE		
bombus_vancouverensis_nearcticus_NW_022882628.1:1-14833	NA	NA	NA	NA	NA	9036-13130	9036-13130	9036-13130, 13463-14581	9036-13130	122-718	FALSE		
bombus_vancouverensis_nearcticus_NW_022882664.1:1-18886	NA	NA	NA	NA	NA	12595-16689	2734-3081, 12595-16689	12595-16689, 17022-18140	3908-4846, 12595-16689	30-662, 3908-4846	FALSE		
bombus_vancouverensis_nearcticus_NW_022882666.1:1-18922	NA	NA	NA	NA	NA	NA	NA	4082-5299	NA	NA	FALSE		
bombus_vancouverensis_nearcticus_NW_022882666.1:21805-39612	NA	NA	NA	NA	NA	NA	NA	5650-7236	NA	NA	FALSE		
bombus_vancouverensis_nearcticus_NW_022882666.1:9959-30816	NA	NA	NA	NA	NA	NA	NA	17496-19082	NA	NA	FALSE		
bombus_vancouverensis_nearcticus_NW_022882673.1:49046-70479	NA	NA	NA	NA	NA	NA	21425-18312, 16242-15895	21425-18312, 2967-2602	21425-18312, 15844-14129	21425-18312, 15844-14129	FALSE		
bombus_vancouverensis_nearcticus_NW_022882772.1:20368-41225	580:1565	6157:7143	576:579/7144:7147	6101:6115	NA	NA	3167-4030	3167-4030, 14853-13810	4244-4627	NA	FALSE		
bombus_vancouverensis_nearcticus_NW_022882772.1:32113-51031	NA	NA	NA	NA	NA	NA	NA	3108-2065	NA	NA	FALSE		
bombus_vancouverensis_nearcticus_NW_022882833.1:12866-26312	NA	NA	NA	NA	NA	NA	NA	5668-6930	NA	NA	FALSE		
bombus_vancouverensis_nearcticus_NW_022882833.1:13205-26312	NA	NA	NA	NA	NA	NA	NA	5329-6591	NA	NA	FALSE		
bombus_vancouverensis_nearcticus_NW_022882833.1:212-21054	NA	NA	NA	NA	NA	NA	NA	5714-6931, 18322-19584	NA	NA	FALSE		
bombus_vancouverensis_nearcticus_NW_022882834.1:1-20323	NA	NA	NA	NA	NA	NA	NA	18175-19392	NA	NA	FALSE		
bombus_vancouverensis_nearcticus_NW_022882835.1:1-12512	NA	NA	NA	NA	NA	NA	NA	10361-11578	NA	NA	FALSE		
bombus_vancouverensis_nearcticus_NW_022882835.1:4326-25168	NA	NA	NA	NA	NA	NA	NA	6036-7253	NA	NA	FALSE		
bombus_vancouverensis_nearcticus_NW_022882856.1:1-19012	NA	NA	NA	NA	NA	NA	NA	14420-13158, 1382-165	NA	NA	FALSE		
bombus_vancouverensis_nearcticus_NW_022882857.1:1-20291	NA	NA	NA	NA	NA	NA	NA	14563-13346	NA	NA	FALSE		
bombus_vancouverensis_nearcticus_NW_022882857.1:12447-23609	NA	NA	NA	NA	NA	NA	NA	2117-900	NA	NA	FALSE		

Sequence	LTR5	LTR3	TSR	PPT	pos_gag	pos_protease	pos_rnase	pos_rt	pos_integrase	pos_transposase	complete	TE	Structure
bombus_vancouverensis_nearcticus_NW_022882859.1:1-12782	NA	NA	NA	NA	NA	NA	NA	7086-5869	8071-9111	NA	FALSE		
bombus_vancouverensis_nearcticus_NW_022882859.1:3341-22699	NA	NA	NA	NA	NA	NA	NA	3746-2529	4731-5771	NA	FALSE		
bombus_vancouverensis_nearcticus_NW_022882864.1:1359-14781	NA	NA	NA	NA	NA	NA	NA	5382-5951	NA	NA	FALSE		
bombus_vancouverensis_nearcticus_NW_022882880.1:3091-23198	NA	NA	NA	NA	NA	NA	NA	15214-13628	NA	NA	FALSE		
bombus_vancouverensis_nearcticus_NW_022882892.1:1-15959	NA	NA	NA	NA	NA	NA	NA	9942-8725	NA	NA	FALSE		
bombus_vancouverensis_nearcticus_NW_022882902.1:1-16141	NA	NA	NA	NA	NA	NA	NA	11224-9815	NA	NA	FALSE		
bombus_vancouverensis_nearcticus_NW_022882917.1:1-11603	NA	NA	NA	NA	NA	NA	NA	5906-4689	NA	NA	FALSE		
bombus_vancouverensis_nearcticus_NW_022882917.1:3726-23367	NA	NA	NA	NA	NA	NA	NA	14811-13594	NA	NA	FALSE		
camponotus_floridanus_NW_020229222.1:647273-668172	5820:7757	18439:20370	5816:5819/20371:20374	7815:7829	NA	16886-14445	14461-12365	16886-14445, 14461-12365	2592-2993, 14461-12365	NA	FALSE		
camponotus_floridanus_NW_020229231.1:448380-469279	NA	NA	NA	NA	NA	NA	NA	NA	7998-8510	NA	FALSE		
camponotus_floridanus_NW_020229251.1:512233-533129	NA	NA	NA	NA	NA	NA	NA	NA	12873-12082	12873-12082, 1567-887	FALSE		
camponotus_floridanus_NW_020229252.1:317397-338302	NA	NA	NA	NA	NA	NA	NA	NA	6214-6924	NA	FALSE		
camponotus_floridanus_NW_020229252.1:396893-417804	7141:9518	17290:19667	7137:7140/19668:19671	9599:9613	20473-19892	NA	15423-11743	15423-11743	15423-11743	NA	FALSE		
camponotus_floridanus_NW_020229252.1:444593-461608	NA	NA	NA	NA	NA	NA	5490-9170	5490-9170	5490-9170	NA	FALSE		
camponotus_floridanus_NW_020229254.1:115719-136612	NA	NA	NA	NA	NA	NA	NA	15049-14075	12792-12391	6774-7478	FALSE		
camponotus_floridanus_NW_020229269.1:357946-378845	NA	NA	NA	NA	NA	NA	7140-7511	6194-6766	7722-8489	8432-8740	FALSE		
camponotus_floridanus_NW_020229275.1:7923-28705	NA	NA	NA	NA	NA	NA	5634-7133, 7161-7523	5634-7133	8130-9158, 18780-17881	8130-9158	FALSE		
camponotus_floridanus_NW_020229290.1:29976-50791	NA	NA	NA	NA	NA	NA	6750-9170	5491-6672	6750-9170	NA	FALSE		
camponotus_floridanus_NW_020229290.1:322260-343162	NA	NA	NA	NA	20834-20049	20026-19220, 17575-16088	6948-8534	6948-8534, 18635-18312	6948-8534, 17575-16088	128-889, 6948-8534	FALSE		
camponotus_floridanus_NW_020229290.1:5276-26187	NA	NA	NA	NA	NA	NA	15423-11743	15423-11743	15423-11743	NA	FALSE		
camponotus_floridanus_NW_020229304.1:113108-133923	NA	NA	NA	NA	NA	NA	5953-6906	4961-5818, 5953-6906, 14392-15783, 20532-17890	NA	NA	FALSE		
camponotus_floridanus_NW_020229320.1:149190-170050	NA	NA	NA	NA	NA	NA	17653-15158	17653-15158	17653-15158	15118-14267	FALSE		
camponotus_floridanus_NW_020229320.1:237088-257999	NA	NA	NA	NA	NA	NA	17590-13910	17590-13910	17590-13910	NA	FALSE		
camponotus_floridanus_NW_020229320.1:267838-288749	NA	NA	NA	NA	NA	NA	5490-9170	5490-9170	5490-9170	NA	FALSE		
camponotus_floridanus_NW_020229320.1:277700-297076	NA	NA	NA	NA	NA	NA	17694-15904	17694-15904	NA	15118-14267	FALSE		
camponotus_floridanus_NW_020229332.1:101402-122313	NA	NA	NA	NA	NA	NA	44-3559	44-3559	44-3559	NA	FALSE		
camponotus_floridanus_NW_020229332.1:14756-35616	NA	NA	NA	NA	NA	NA	NA	NA	NA	5656-6591	FALSE		
camponotus_floridanus_NW_020229332.1:67581-88441	NA	NA	NA	NA	NA	NA	NA	NA	15359-14268	15359-14268	FALSE		
camponotus_floridanus_NW_020229332.1:95791-116702	NA	NA	NA	NA	NA	NA	5490-9170	5490-9170	5490-9170	NA	FALSE		
camponotus_floridanus_NW_020229343.1:1-19222	NA	NA	NA	NA	NA	NA	1646-4879	696-1655, 1646-4879	1646-4879, 18305-19222	1646-4879, 18305-19222	FALSE		
camponotus_floridanus_NW_020229343.1:26825-47685	NA	NA	NA	NA	NA	NA	18250-14270	18250-14270	18250-14270	18250-14270	FALSE		
camponotus_floridanus_NW_020229345.1:5840-26700	6225:6575, 6259:6404	20500:20849, 20534:20679	6221:6224/20850:20853, 6254:6258/20680:20684	20456:20470	NA	18236-14271	18236-14271	18236-14271	18236-14271	18236-14271	FALSE		
camponotus_floridanus_NW_020229349.1:103302-124162	NA	NA	NA	NA	NA	NA	NA	NA	13473-14189	13473-14189	FALSE		
camponotus_floridanus_NW_020229357.1:181227-202006	674:2641	15172:17112	670:673/17113:17116	15100:15114	NA	NA	6831-7730	6155-6781	8032-8643, 13088-14092	13088-14092	FALSE		

Sequence	LTR5	LTR3	TSR	PPT	pos_gag	pos_protease	pos_rnase	pos_rt	pos_integrase	pos_transposase	complete	TE	Structure
camponotus_floridanus_NW_020229362.1:110568-131479	1366:3623	11395:13653	1362:1365/13654:13657	11300:11314	NA	NA	5654-9169, 17492-17800, 17809-18138	5654-9169, 16900-17559, 17492-17800	5654-9169, 13809-14735	13809-14735	FALSE		
camponotus_floridanus_NW_020229362.1:88304-109215	16321:17321	19799:20801	16316:16320/20802:20806	19758:19772	NA	NA	6204-9170	6204-9170	6204-9170	NA	FALSE		
camponotus_floridanus_NW_020229378.1:148326-169102	NA	NA	NA	NA	NA	19474-20775	NA	19474-20775	NA	NA	FALSE		
camponotus_floridanus_NW_020229378.1:165877-186782	22:571	17940:18489	NOTFOUND	17864:17878	NA	1923-6176	1923-6176	1923-6176	1923-6176	1923-6176	FALSE		
camponotus_floridanus_NW_020229383.1:29927-50832	NA	NA	NA	NA	NA	1108-1413	NA	NA	NA	NA	FALSE		
camponotus_floridanus_NW_020229383.1:79656-100561	NA	NA	NA	NA	NA	NA	NA	NA	NA	6091-7173	FALSE		
camponotus_floridanus_NW_020229383.1:99533-120438	NA	NA	NA	NA	NA	18907-16121	18907-16121	18907-16121	16182-15718, 15727-14786	7584-8666	FALSE		
camponotus_floridanus_NW_020229391.1:149718-170578	NA	NA	NA	NA	17965-13982	17965-13982	NA	17965-13982	17965-13982	6144-7124	FALSE		
camponotus_floridanus_NW_020229409.1:22949-43860	NA	NA	NA	NA	NA	NA	15423-11743	15423-11743	15423-11743	20481-20912	FALSE		
camponotus_floridanus_NW_020229426.1:10776-31636	NA	NA	NA	NA	NA	NA	19170-17077	19170-17077	17019-15463	17019-15463	FALSE		
camponotus_floridanus_NW_020229429.1:106975-127877	NA	NA	NA	NA	NA	NA	NA	NA	NA	4168-3086	FALSE		
camponotus_floridanus_NW_020229451.1:18954-39814	NA	NA	NA	NA	NA	2627-5704	2627-5704	2627-5704	2627-5704	5647-6591	FALSE		
camponotus_floridanus_NW_020229451.1:57990-78895	NA	NA	NA	NA	NA	3209-4567	NA	3209-4567	NA	NA	FALSE		
camponotus_floridanus_NW_020229454.1:97550-116311	NA	NA	NA	NA	NA	2121-5366	2121-5366	2121-5366	2121-5366, 5351-6148	NA	FALSE		
camponotus_floridanus_NW_020229461.1:46269-67174	NA	NA	NA	NA	NA	NA	NA	NA	4614-6152	4614-6152, 14032-12899	FALSE		
camponotus_floridanus_NW_020229461.1:62557-83462	2521:3086	20332:20897	NOTFOUND	3160:3174	NA	18980-14736	18980-14736	18980-14736	18980-14736	18980-14736	FALSE		
camponotus_floridanus_NW_020229474.1:1-15002	NA	NA	NA	NA	NA	NA	NA	NA	9579-8782	NA	FALSE		
camponotus_floridanus_NW_020229474.1:40818-61723	NA	NA	NA	NA	NA	NA	20813-20241	NA	19567-18497	45-1127, 19567-18497, 14941-13682	FALSE		
camponotus_floridanus_NW_020229489.1:45107-66012	NA	NA	NA	NA	NA	NA	4917-1888	4917-1888	4917-1888	NA	FALSE		
camponotus_floridanus_NW_020229490.1:137965-158750	NA	NA	NA	NA	NA	NA	20716-16958	20716-16958	14318-14860, 20716-16958	13780-14418, 20716-16958	FALSE		
camponotus_floridanus_NW_020229495.1:38502-59413	NA	NA	NA	NA	NA	6323-3765	15258-11743, 6323-3765	15258-11743, 6323-3765	15258-11743	NA	FALSE		
camponotus_floridanus_NW_020229495.1:8771-29676	NA	NA	NA	NA	NA	NA	20880-17032	20880-17032	20880-17032	NA	FALSE		
camponotus_floridanus_NW_020229499.1:1-19462	NA	NA	NA	NA	NA	1229-2494	2416-5193, 13863-15176	1229-2494, 2416-5193	2416-5193, 13863-15176, 15079-15999	2416-5193	FALSE		
camponotus_floridanus_NW_020229499.1:64051-84956	2792:3334	20331:20873	NOTFOUND	3386:3400	NA	18979-18392	17671-14726	17671-14726	17671-14726	17671-14726	FALSE		
camponotus_floridanus_NW_020229499.1:6828-27739	NA	NA	NA	NA	NA	NA	7036-8349	NA	7036-8349, 8252-9172	NA	FALSE		
camponotus_floridanus_NW_020229507.1:10799-31704	NA	NA	NA	NA	NA	NA	NA	NA	20716-19253	20716-19253	FALSE		
camponotus_floridanus_NW_020229515.1:55631-76536	NA	NA	NA	NA	NA	2114-4258	2114-4258	2114-4258, 18545-19588	5067-6134	5067-6134	FALSE		
camponotus_floridanus_NW_020229517.1:10660-31571	NA	NA	NA	NA	NA	2672-6700	2672-6700, 15423-11743	2672-6700, 15423-11743	2672-6700, 15423-11743	2672-6700	FALSE		
camponotus_floridanus_NW_020229517.1:23470-44309	NA	NA	NA	NA	NA	NA	2613-1	2613-1	NA	NA	FALSE		
camponotus_floridanus_NW_020229527.1:31770-52675	NA	NA	NA	NA	NA	NA	NA	NA	NA	9159-8191	FALSE		
camponotus_floridanus_NW_020229535.1:1-19411	NA	NA	NA	NA	NA	2701-6798	2701-6798	2701-6798	2701-6798	6805-7662	FALSE		
camponotus_floridanus_NW_020229537.1:19123-40028	NA	NA	NA	NA	NA	NA	NA	NA	NA	7785-8315	FALSE		
camponotus_floridanus_NW_020229537.1:60193-72325	NA	NA	NA	NA	NA	NA	NA	NA	2738-3268	2187-2837	FALSE		
camponotus_floridanus_NW_020229542.1:10184-31110	NA	NA	NA	NA	NA	NA	1576-1878, 6781-7500	1576-1878, 5825-6391, 15874-16617	2767-3279, 7968-8471	8414-8755	FALSE		
camponotus_floridanus_NW_020229546.1:19308-40213	NA	NA	NA	NA	NA	18821-14796	18821-14796	18821-14796	18821-14796	18821-14796	FALSE		
camponotus_floridanus_NW_020229567.1:1-13280	NA	NA	NA	NA	NA	11345-7101	11345-7101	11345-7101	11345-7101	11345-7101	FALSE		
camponotus_floridanus_NW_020229568.1:1-17890	NA	NA	NA	NA	NA	NA	14573-11748	17672-17004, 14573-11748	14573-11748	14573-11748	FALSE		
camponotus_floridanus_NW_020229571.1:74376-90815	NA	NA	NA	NA	NA	2931-6116	2931-6116	2931-6116	NA	NA	FALSE		

Sequence	LTR5	LTR3	TSR	PPT	pos_gag	pos_protease	pos_rnase	pos_rt	pos_integrase	pos_transposase	complete	TE	Structure
camponotus_floridanus_NW_020229581.1:1925576-1946442	NA	NA	NA	NA	NA	NA	NA	NA	NA	18471-19361, 4917-3832	FALSE		
camponotus_floridanus_NW_020229605.1:12569-33474	NA	NA	NA	NA	NA	7458-8432	NA	8456-8908	NA	NA	FALSE		
camponotus_floridanus_NW_020229605.1:49776-69855	NA	NA	NA	NA	NA	18781-14753	18781-14753	18781-14753	18781-14753	18781-14753	FALSE		
camponotus_floridanus_NW_020229611.1:53830-73755	NA	NA	NA	NA	NA	NA	15331-11750	15331-11750	15331-11750	15331-11750, 1933-851	FALSE		
camponotus_floridanus_NW_020229615.1:22807-43712	NA	NA	NA	NA	NA	NA	NA	NA	5189-6121	NA	FALSE		
camponotus_floridanus_NW_020229616.1:27966-43883	NA	NA	NA	NA	NA	NA	14565-11743	15008-14487, 14565-11743	14565-11743	NA	FALSE		
camponotus_floridanus_NW_020229617.1:5069-25962	NA	NA	NA	NA	NA	NA	NA	14975-14583	13544-13059, 5206-4181	13030-12638, 5206-4181	FALSE		
camponotus_floridanus_NW_020229618.1:2018-22917	NA	NA	NA	NA	NA	NA	NA	NA	13153-12092	13153-12092	FALSE		
camponotus_floridanus_NW_020229629.1:23647-40708	NA	NA	NA	NA	NA	NA	16927-16352, 16311-15901	16927-16352	NA	14947-14264	FALSE		
camponotus_floridanus_NW_020229645.1:253-21158	NA	NA	NA	NA	NA	2114-2899	2875-6576	2875-6576	2875-6576	2875-6576, 20896-20009	FALSE		
camponotus_floridanus_NW_020229663.1:4695-25555	NA	NA	NA	NA	NA	NA	NA	NA	5600-6694	5600-6694	FALSE		
camponotus_floridanus_NW_020229665.1:1-14569	NA	NA	NA	NA	NA	12622-8378	12622-8378	12622-8378	12622-8378	12622-8378	FALSE		
camponotus_floridanus_NW_020229676.1:1-16224	NA	NA	NA	NA	NA	343-4482	343-4482	343-4482	343-4482	NA	FALSE		
camponotus_floridanus_NW_020229679.1:17286-29567	NA	NA	NA	NA	NA	NA	NA	2662-1208	NA	NA	FALSE		
camponotus_floridanus_NW_020229683.1:1-16995	NA	NA	NA	NA	NA	14865-10888	14865-10888	14865-10888	14865-10888	NA	FALSE		
camponotus_floridanus_NW_020229813.1:1-12897	NA	NA	NA	NA	NA	NA	1122-2849	8-997, 1122-2849	2837-3931	2837-3931	FALSE		
cephus_cinctus_NW_014332819.1:1-14740	NA	NA	NA	NA	NA	NA	NA	NA	35-1288	NA	FALSE		
cephus_cinctus_NW_014332969.1:1-12923	NA	NA	NA	NA	NA	7414-7055	6365-4032	6365-4032	4624-3857, 3810-3511	NA	FALSE		
cephus_cinctus_NW_014333061.1:16961-38763	NA	NA	NA	NA	NA	NA	NA	4504-4016, 3956-3591	8014-9177, 2684-1956	8014-9177	FALSE		
cephus_cinctus_NW_014333126.1:1-12084	NA	NA	NA	NA	NA	7870-10314	10336-10899, 10939-12084	7870-10314, 10336-10899	10939-12084	NA	FALSE		
cephus_cinctus_NW_014333170.1:1-14351	NA	NA	NA	NA	NA	NA	NA	10092-10391, 10360-11043, 11269-11637	NA	NA	FALSE		
cephus_cinctus_NW_014333275.1:1-14869	NA	NA	NA	NA	NA	NA	NA	NA	3534-5720	3534-5720	FALSE		
cephus_cinctus_NW_014334081.1:7375-28274	NA	NA	NA	NA	NA	NA	NA	NA	NA	6265-6606, 6555-7313	FALSE		
cinara_cedri_CABPRJ010001454.1:5168-25686	NA	NA	NA	NA	NA	NA	NA	12416-11613	NA	NA	FALSE		
cinara_cedri_CABPRJ010001900.1:67196-87657	NA	NA	NA	NA	NA	NA	1013-1372	NA	NA	NA	FALSE		
culex_pipiens_pallens_NW_024109004.1:555841-577559	3365:3907	11835:12377	3360:3364/12378:12382	11742:11756	4487-11788	4487-11788	NA	4487-11788	4487-11788	NA	FALSE		
culex_pipiens_pallens_NW_024109043.1:277988-299706	NA	NA	NA	NA	2251-4179	2251-4179, 6434-11788	NA	6434-11788	6434-11788	NA	FALSE		
culex_pipiens_pallens_NW_024109046.1:1044778-1066742	8576:9310	14223:14960	8571:8575/14961:14965	9331:9345	NA	NA	NA	NA	NA	NA	FALSE		
culex_pipiens_pallens_NW_024109060.1:1175102-1197015	4897:5252	20627:20982	4893:4896/20983:20986	5253:5267	21825-21031, 17263-10001	17263-10001	NA	17263-10001	17263-10001	NA	FALSE		
culex_pipiens_pallens_NW_024109077.1:813205-834923	9343:9885	17797:18339	9338:9342/18340:18344	9964:9978	17041-9932	17041-9932	NA	17041-9932	17041-9932	NA	FALSE		
culex_pipiens_pallens_NW_024109200.1:1-12385	19:551	8480:9012	14:18/9013:9017	630:644	7707-598	7707-598	NA	7707-598	7707-598	NA	FALSE		
culex_pipiens_pallens_NW_024109213.1:526597-548510	1693:2402	12540:13249	1688:1692/13250:13254	12452:12466	4652-11914	4652-11914	NA	4652-11914	4652-11914	NA	FALSE		
culex_pipiens_pallens_NW_024109334.1:351915-373633	9343:9885	17817:18359	9338:9342/18360:18364	9964:9978	17041-9932	17041-9932	NA	17041-9932	17041-9932	NA	FALSE		
culex_pipiens_pallens_NW_024109674.1:1-15641	NA	NA	NA	NA	10963-3668	10963-3668	NA	10963-3668	NA	NA	FALSE		

Sequence	LTR5	LTR3	TSR	PPT	pos_gag	pos_protease	pos_rnase	pos_rt	pos_integrase	pos_transposase	complete	TE	Structure
culex_pipiens_pallens_NW_024109703.1:104229-126052	8826:9029	17588:17791	8821:8825/17792:17796	17573:17587	NA	16833-17372, 14845-9851	NA	14845-9851	14845-9851	NA	FALSE		
culex_pipiens_pallens_NW_024109703.1:109075-130058	3980:4183	12742:12945	3975:3979/12946:12950	12727:12741	NA	11987-12526, 9999-5005	NA	9999-5005	9999-5005	NA	FALSE		
culex_pipiens_pallens_NW_024109766.1:135893-157053	3246:3589	12044:12387	3241:3245/12388:12392	11990:12004	4326-10013	4326-10013	NA	4326-10013	4326-10013	NA	FALSE		
culex_pipiens_pallens_NW_024110117.1:1-12354	NA	NA	NA	NA	7868-567	7868-567	NA	7868-567	7868-567	NA	FALSE		
cyphomyrmex_costatus_NW_017275046.1:45309-67375	NA	NA	NA	NA	NA	NA	5975-9160	5975-9160	5975-9160, 9130-9993	NA	FALSE		
cyphomyrmex_costatus_NW_017275161.1:35989-58028	NA	NA	NA	NA	NA	18982-20532	5838-8996, 21175-22038	5838-8996, 18982-20532	5838-8996, 21175-22038	9090-9941	FALSE		
cyphomyrmex_costatus_NW_017275161.1:36046-57308	NA	NA	NA	NA	NA	18925-20475	5781-8939	5781-8939, 18925-20475	5781-8939	9033-9884	FALSE		
cyphomyrmex_costatus_NW_017275173.1:8147-29499	NA	NA	NA	NA	NA	NA	15518-12300	15518-12300	15518-12300	1192-1575, 12145-11834	FALSE		
cyphomyrmex_costatus_NW_017275314.1:1-12858	NA	NA	NA	NA	NA	8891-6396	6245-3795	8891-6396, 6245-3795	12835-11861, 6245-3795	12835-11861	FALSE		
cyphomyrmex_costatus_NW_017275315.1:1115609-1137318	NA	NA	NA	NA	NA	NA	15736-14912	16789-15782, 5961-4357, 4297-3857	NA	12492-12136	FALSE		
cyphomyrmex_costatus_NW_017275547.1:6327-28330	NA	NA	NA	NA	21414-22004	5061-9971	5061-9971	5061-9971	5061-9971	5061-9971	FALSE		
cyphomyrmex_costatus_NW_017276402.1:59722-81725	2332:3921	12056:13645	2328:2331/13646:13649	12041:12055	NA	5882-6532	7575-9599	7186-7488, 17978-16254	7575-9599	7575-9599	FALSE		
cyphomyrmex_costatus_NW_017276608.1:1-13335	NA	NA	NA	NA	NA	NA	NA	NA	2972-4387	NA	FALSE		
cyphomyrmex_costatus_NW_017276801.1:1-14728	NA	NA	NA	NA	NA	NA	7848-7414	9896-8490, 8483-7803	6595-4751	NA	FALSE		
cyphomyrmex_costatus_NW_017277232.1:96879-117661	NA	NA	NA	NA	NA	NA	3248-4051	3248-4051	4654-5250	4654-5250	FALSE		
cyphomyrmex_costatus_NW_017277578.1:24303-46321	NA	NA	NA	NA	NA	16125-14371, 5301-2923	16125-14371, 14419-14066, 2871-313	16125-14371, 14419-14066, 2871-313	13986-12970, 12963-12025, 2871-313	NA	FALSE		
cyphomyrmex_costatus_NW_017277687.1:22367-44136	NA	NA	NA	NA	NA	NA	21268-18983, 15548-14298, 14134-12035	15548-14298	21268-18983, 14134-12035	NA	FALSE		
cyphomyrmex_costatus_NW_017277720.1:79783-100622	NA	NA	NA	NA	NA	NA	5003-8902	5003-8902	5003-8902	NA	FALSE		
cyphomyrmex_costatus_NW_017278716.1:1981-23945	NA	NA	NA	NA	NA	18883-17639	16948-16103	18883-17639, 17632-17246	13466-12033	NA	FALSE		
cyphomyrmex_costatus_NW_017278716.1:2026-24005	NA	NA	NA	NA	NA	18838-17594	16903-16058	18838-17594, 17587-17201	13421-11988	NA	FALSE		
cyphomyrmex_costatus_NW_017279084.1:95760-117679	NA	NA	NA	NA	NA	NA	6521-7534, 7552-8064	6521-7534	8100-9467	8100-9467	FALSE		
cyphomyrmex_costatus_NW_017279244.1:502025-523881	NA	NA	NA	NA	NA	NA	8282-8581	NA	NA	8619-8972, 6174-5782	FALSE		
cyphomyrmex_costatus_NW_017279343.1:2274-17755	4340:4982	8971:9613	4335:4339/9614:9618	8927:8941	NA	NA	1090-2628	NA	4325-4993, 8551-9984	NA	FALSE		
cyphomyrmex_costatus_NW_017279637.1:9478-31490	3071:4617	12048:13594	3067:3070/13595:13598	11990:12004	NA	NA	6162-9971	6162-9971, 600-1	6162-9971	NA	FALSE		
cyphomyrmex_costatus_NW_017279671.1:8758-29798	NA	NA	NA	NA	NA	NA	7434-9980	6728-7087	7434-9980	7434-9980	FALSE		
cyphomyrmex_costatus_NW_017279900.1:2276-24261	NA	NA	NA	NA	NA	NA	NA	NA	8425-9141	NA	FALSE		
cyphomyrmex_costatus_NW_017280214.1:140654-161463	NA	NA	NA	NA	NA	NA	18005-17646	18477-18052, 18005-17646	938-1264, 16573-15983	938-1264	FALSE		
cyphomyrmex_costatus_NW_017280575.1:11196-33208	3071:4617	12048:13594	3067:3070/13595:13598	11990:12004	NA	NA	6162-9971	6162-9971	6162-9971	NA	FALSE		
cyphomyrmex_costatus_NW_017280585.1:2668-24323	NA	NA	NA	NA	NA	15817-14150	14086-13316	15817-14150	13359-11656	13359-11656	FALSE		
diachasma_alloeum_NW_021680380.1:312721-334298	NA	NA	NA	NA	16803-17261	5517-6248	6143-9886	6143-9886	6143-9886	6143-9886	FALSE		

Sequence	LTR5	LTR3	TSR	PPT	pos_gag	pos_protease	pos_rnase	pos_rt	pos_integrase	pos_transposase	complete	TE	Structure
diachasma_alloeum_NW_021680380.1:322284-343861	NA	NA	NA	NA	7240-7698	NA	18471-19751	18471-19751	7748-9886, 20344-21267	7748-9886, 17189-18016	FALSE		
diachasma_alloeum_NW_021680383.1:305850-327427	NA	NA	NA	NA	NA	NA	NA	19769-20929	8643-9035	8974-9585	FALSE		
diachasma_alloeum_NW_021680394.1:389647-403825	NA	NA	NA	NA	NA	NA	2565-3212	1809-2540, 2565-3212	3363-4736	3363-4736	FALSE		
diachasma_alloeum_NW_021680423.1:83560-105137	NA	NA	NA	NA	NA	NA	15511-13235	15511-13235	NA	13293-11713	FALSE		
diachasma_alloeum_NW_021680456.1:182768-204339	NA	NA	NA	NA	NA	NA	14251-13667	14667-14245	13615-12671	12667-12056	FALSE		
diachasma_alloeum_NW_021680456.1:643118-664692	NA	NA	NA	NA	NA	NA	14863-11681	16085-14853, 14863-11681	14863-11681	14863-11681	FALSE		
diachasma_alloeum_NW_021680458.1:137869-159344	5:956	14701:15652	NOTFOUND	14634:14648	NA	2636-5341	2636-5341	2636-5341	7581-9506	7581-9506	FALSE		
diachasma_alloeum_NW_021680458.1:244519-265904	NA	NA	NA	NA	NA	NA	NA	NA	13997-13653, 12616-11825	NA	FALSE		
diachasma_alloeum_NW_021680470.1:1-20251	33:1286	11012:12265	NOTFOUND	10983:10997	NA	NA	4692-5693, 5981-6505, 14109-15509	3328-4584, 4692-5693, 14109-15509	7474-8559, 16782-17639	7474-8559, 17661-18269	FALSE		
diachasma_alloeum_NW_021680489.1:404308-425594	NA	NA	NA	NA	NA	668-1576	6740-9886	1576-2079, 6740-9886	4241-6589, 6740-9886	6740-9886	FALSE		
diachasma_alloeum_NW_021680496.1:585162-597517	NA	NA	NA	NA	NA	5549-6262	NA	6232-7203	NA	8892-9566	FALSE		
diachasma_alloeum_NW_021680578.1:94019-115440	1970:3801	12657:14468	1966:1969/14469:14472	3834:3848	NA	NA	NA	5416-7245	NA	8739-9746	FALSE		
diachasma_alloeum_NW_021680643.1:102434-124008	NA	NA	NA	NA	NA	NA	7072-7602	6245-7060	8844-9254	9122-9571	FALSE		
diachasma_alloeum_NW_021680654.1:87945-109519	NA	NA	NA	NA	4795-6168	6336-7019, 18820-19500	NA	18820-19500, 16076-14304	13699-11690	13699-11690	FALSE		
diachasma_alloeum_NW_021680662.1:193109-214686	NA	NA	NA	NA	457-1518	1525-2454, 2539-4116	18697-16853, 16034-12906	2539-4116, 20804-17319, 16034-12906	4349-5395	4349-5395, 12659-11958	FALSE		
diachasma_alloeum_NW_021680662.1:34350-55918	NA	NA	NA	NA	NA	NA	2707-5544	2707-5544	2707-5544	2707-5544	FALSE		
diachasma_alloeum_NW_021680700.1:29016-50596	NA	NA	NA	NA	NA	NA	806-3319	806-3319	3389-4603	3389-4603	FALSE		
diachasma_alloeum_NW_021680731.1:81482-103059	NA	NA	NA	NA	NA	NA	15835-11693	656-1114, 15835-11693, 7006-6353	15835-11693	15835-11693	FALSE		
diachasma_alloeum_NW_021680762.1:81450-103027	NA	NA	NA	NA	NA	NA	NA	NA	13342-12674	12619-12278	FALSE		
diachasma_alloeum_NW_021680791.1:186185-207762	NA	NA	NA	NA	NA	NA	18409-11693	18409-11693	18409-11693	18409-11693	FALSE		
diachasma_alloeum_NW_021680801.1:102450-123784	NA	NA	NA	NA	NA	NA	3929-4777	21117-20779, 20900-19584	8630-9208	NA	FALSE		
diachasma_alloeum_NW_021680810.1:112795-134129	NA	NA	NA	NA	NA	20522-19098	NA	20522-19098	NA	6840-7400	FALSE		
diachasma_alloeum_NW_021680886.1:56031-77500	NA	NA	NA	NA	NA	NA	NA	16911-15571, 15575-15084, 1211-3	12778-9464	12778-9464, 5727-5305	FALSE		
diachasma_alloeum_NW_021680939.1:1-21180	16346:16452	18020:18130	16342:16345/18131:18134	17927:17941	NA	NA	5115-9488	5115-9488	5115-9488	5115-9488	FALSE		
diachasma_alloeum_NW_021680988.1:72166-93743	NA	NA	NA	NA	NA	NA	NA	NA	8081-9880	8081-9880	FALSE		
diachasma_alloeum_NW_021681033.1:71537-92979	7737:9446	17452:19087	7733:7736/19088:19091	9517:9531	NA	NA	14218-13085	2073-3722, 15764-14154, 14218-13085	NA	NA	FALSE		
diachasma_alloeum_NW_021681046.1:165270-177730	NA	NA	NA	NA	2-976	2-976, 2093-2596	NA	1626-2153	3111-3560	NA	FALSE		
diachasma_alloeum_NW_021681065.1:105293-126870	NA	NA	NA	NA	NA	NA	1399-3621, 16624-16974	1399-3621	NA	NA	FALSE		
diachasma_alloeum_NW_021681066.1:9759-31339	NA	NA	NA	NA	NA	NA	19326-17632	19326-17632	17495-16476	17495-16476	FALSE		
diachasma_alloeum_NW_021681102.1:1-16891	NA	NA	NA	NA	NA	1918-4011	1918-4011	1918-4011	4113-5204	4113-5204	FALSE		
diachasma_alloeum_NW_021681230.1:25563-47131	NA	NA	NA	NA	1807-2292	2831-3937, 3956-4864	14513-12360	2831-3937, 3956-4864, 14513-12360	4868-6646, 12372-11611	4868-6646	FALSE		
diachasma_alloeum_NW_021681243.1:1456-7754	NA	NA	NA	NA	NA	NA	4488-115	4488-115	4488-115	4488-115	FALSE		
diachasma_alloeum_NW_021681288.1:9324-26281	NA	NA	NA	NA	NA	NA	10514-11179	NA	NA	NA	FALSE		

Sequence	LTR5	LTR3	TSR	PPT	pos_gag	pos_protease	pos_rnase	pos_rt	pos_integrase	pos_transposase	complete	TE	Structure
eufriesea_mexicana_NW_016907970.1:271392-292669	NA	NA	NA	NA	NA	17806-17471	21239-20901, 16413-15685, 7221-6874	16413-15685, 4543-4229	NA	1247-813	FALSE		
eufriesea_mexicana_NW_016908258.1:98346-119551	NA	NA	NA	NA	NA	NA	NA	12776-13402, 5322-4852, 3695-2970, 2936-2436	NA	NA	FALSE		
eufriesea_mexicana_NW_016909960.1:387551-408741	NA	NA	NA	NA	NA	NA	13609-13226	6918-7223, 14314-13694, 13609-13226	NA	NA	FALSE		
eufriesea_mexicana_NW_016914852.1:8188-29459	NA	NA	NA	NA	NA	12928-13884	15199-15735	14106-14855, 5668-5360	8806-9936, 15680-16561, 4471-3989	NA	FALSE		
eufriesea_mexicana_NW_016935052.1:1-7159	NA	NA	NA	NA	NA	NA	5706-1996	5706-1996	5706-1996	5706-1996	FALSE		
habropoda_laboriosa_NW_017100133.1:26349-47869	2733:3871	12007:13168	2729:2732/13169:13172	11992:12006	1319-678	6060-7385	7979-11950	6060-7385, 7343-7822	7979-11950, 20710-20309	7979-11950, 412-56	FALSE		
habropoda_laboriosa_NW_017100133.1:34843-55991	NA	NA	NA	NA	15480-20417	15480-20417	13397-12957	15480-20417, 14071-13331	124-3456, 15480-20417, 12216-11815	124-3456	FALSE		
habropoda_laboriosa_NW_017100158.1:335788-350905	NA	NA	NA	NA	775-413	6767-6312	NA	NA	14997-14221, 12796-11963, 6280-5441	14997-14221, 12796-11963, 2621-2145	FALSE		
habropoda_laboriosa_NW_017100159.1:434174-455103	NA	NA	NA	NA	NA	NA	NA	NA	NA	8324-7296	FALSE		
habropoda_laboriosa_NW_017100283.1:25130-46059	NA	NA	NA	NA	NA	NA	14918-14424, 14380-10001	17897-17367, 17424-16921	14380-10001	NA	FALSE		
habropoda_laboriosa_NW_017100326.1:56277-78316	NA	NA	NA	NA	NA	18420-17254	17240-16311	18420-17254, 17240-16311	15771-15343, 14306-14001, 13840-11999	13840-11999, 9010-8258, 4615-3569, 3179-1374	FALSE		
habropoda_laboriosa_NW_017100542.1:117724-139301	NA	NA	NA	NA	NA	NA	7120-9858	7120-9858	7120-9858, 19289-18807, 4474-3614	21551-20556, 19289-18807, 16808-16011, 13136-12732, 4923-4585, 3242-1848	FALSE		
habropoda_laboriosa_NW_017100814.1:103119-116698	NA	NA	NA	NA	NA	NA	NA	NA	12312-11884	11884-10001, 1199-759	FALSE		
habropoda_laboriosa_NW_017100933.1:1759-9271	NA	NA	NA	NA	NA	2145-2534	2471-6574	2471-6574	2471-6574	2471-6574	FALSE		
habropoda_laboriosa_NW_017101288.1:13477-15216	NA	NA	NA	NA	NA	NA	NA	NA	NA	856-1740	FALSE		
harpegnathos_saltator_NW_020229876.1:1-14226	1556:2500	7595:8540	1552:1555/8541:8544	2501:2515	NA	10320-9433	NA	NA	7470-6424	NA	FALSE		
harpegnathos_saltator_NW_020229876.1:32814-54541	NA	NA	NA	NA	NA	NA	NA	NA	NA	14530-13925	FALSE		
harpegnathos_saltator_NW_020229899.1:222601-244148	NA	NA	NA	NA	NA	NA	NA	NA	2045-2596, 16797-17555, 18614-17691	3428-4174, 18614-17691	FALSE		
harpegnathos_saltator_NW_020229917.1:686740-708425	NA	NA	NA	NA	NA	NA	21473-20433	21473-20433	NA	19413-18922	FALSE		
harpegnathos_saltator_NW_020229920.1:40437-62161	NA	NA	NA	NA	NA	NA	3472-7824	3472-7824	3472-7824	NA	FALSE		
harpegnathos_saltator_NW_020229932.1:1-19446	NA	NA	NA	NA	NA	2256-2981	4040-6016	4040-6016	6207-7433	6207-7433	FALSE		
harpegnathos_saltator_NW_020229932.1:16410-37708	NA	NA	NA	NA	NA	NA	14435-12987	15378-15013, 15013-14714	NA	12702-12169	FALSE		
harpegnathos_saltator_NW_020229932.1:17295-38029	NA	NA	NA	NA	NA	NA	13550-12102	14493-14128, 14128-13829	NA	11817-11284	FALSE		
harpegnathos_saltator_NW_020229937.1:18248-38862	NA	NA	NA	NA	NA	NA	1885-1235	14145-13660, 3186-1891, 1885-1235	544-80	NA	FALSE		
harpegnathos_saltator_NW_020229937.1:6079-26693	NA	NA	NA	NA	NA	NA	14054-13404	15355-14060, 14054-13404	8108-8734, 12713-12249, 12212-11754, 1026-676	8108-8734, 1026-676	FALSE		
harpegnathos_saltator_NW_020229946.1:1138074-1159537	NA	NA	NA	NA	NA	17198-16578	15393-13162	15393-13162	13125-12481	13125-12481	FALSE		
harpegnathos_saltator_NW_020229948.1:665805-686527	NA	NA	NA	NA	NA	NA	6273-9713	6273-9713	6273-9713, 12779-12114	6273-9713	FALSE		
harpegnathos_saltator_NW_020229960.1:1-14057	2:956	12640:13604	NOTFOUND	957:971	NA	9783-9043	8219-5949	8219-5949	5827-4601	5827-4601	FALSE		

Sequence	LTR5	LTR3	TSR	PPT	pos_gag	pos_protease	pos_rnase	pos_rt	pos_integrase	pos_transposase	complete	TE	Structure
harpegnathos_saltator_NW_020229972.1:1-12426	NA	NA	NA	NA	NA	NA	NA	NA	323-1093, 8120-7662	323-1093, 8708-8184, 8120-7662	FALSE		
harpegnathos_saltator_NW_020229972.1:1-13446	NA	NA	NA	NA	NA	NA	NA	NA	323-1093, 12567-12139, 8120-7662	323-1093, 12567-12139, 8708-8184, 8120-7662	FALSE		
harpegnathos_saltator_NW_020229991.1:196514-217824	NA	NA	NA	NA	17691-16936	16881-15973, 15747-14176, 14503-13595	7144-7542	302-1054, 813-2156, 5958-6275, 6324-6776, 15747-14176	8002-8862, 13839-12430, 4968-4561	NA	FALSE		
harpegnathos_saltator_NW_020229995.1:134876-153584	NA	NA	NA	NA	NA	NA	5132-9775	5132-9775	5132-9775, 16688-16020	5132-9775	FALSE		
harpegnathos_saltator_NW_020229995.1:135197-153584	NA	NA	NA	NA	NA	NA	4811-9454	4811-9454	4811-9454, 16367-15699	4811-9454	FALSE		
harpegnathos_saltator_NW_020230004.1:221725-242453	NA	NA	NA	NA	NA	NA	13728-12460	13728-12460	12441-11212	12441-11212	FALSE		
harpegnathos_saltator_NW_020230004.1:231645-243816	NA	NA	NA	NA	NA	NA	3808-2540	3808-2540	2521-1292	2521-1292	FALSE		
harpegnathos_saltator_NW_020230016.1:128343-149071	NA	NA	NA	NA	NA	3149-3607	NA	6015-6461, 6471-7118	5107-5520, 8560-8979	5107-5520, 12702-13784, 14777-14223	FALSE		
harpegnathos_saltator_NW_020230032.1:113519-128733	NA	NA	NA	NA	NA	NA	15175-12008	15175-12008	5676-6287, 15175-12008	5676-6287, 6312-6713, 15175-12008	FALSE		
harpegnathos_saltator_NW_020230032.1:114572-128733	NA	NA	NA	NA	NA	NA	14122-10955	14122-10955	4623-5234, 14122-10955	4623-5234, 5259-5660, 14122-10955	FALSE		
harpegnathos_saltator_NW_020230045.1:578-22269	NA	NA	NA	NA	NA	3538-5325	5283-7967	3538-5325, 5283-7967	5283-7967	5283-7967	FALSE		
harpegnathos_saltator_NW_020230053.1:19406-40743	NA	NA	NA	NA	NA	16359-15922	14903-12384	15297-14881, 14903-12384	14903-12384	NA	FALSE		
harpegnathos_saltator_NW_020230058.1:277475-298935	NA	NA	NA	NA	NA	NA	6380-7192, 7276-7752	6380-7192	7568-8599	8503-9132	FALSE		
harpegnathos_saltator_NW_020230066.1:1-11220	NA	NA	NA	NA	NA	6990-5479	5467-4166, 4138-3143	5467-4166	3221-2403	2375-1944	FALSE		
harpegnathos_saltator_NW_020230066.1:73054-91720	NA	NA	NA	NA	NA	NA	14271-13744	NA	13775-12837	NA	FALSE		
harpegnathos_saltator_NW_020230068.1:196414-217097	NA	NA	NA	NA	NA	NA	15301-14597	4850-2280	NA	13501-13091	FALSE		
harpegnathos_saltator_NW_020230075.1:6144-27835	NA	NA	NA	NA	NA	NA	16475-13725	16475-13725	16475-13725	16475-13725	FALSE		
harpegnathos_saltator_NW_020230075.1:88500-105200	NA	NA	NA	NA	NA	NA	16253-14412	16642-16256, 16253-14412	16253-14412	14532-13726	FALSE		
harpegnathos_saltator_NW_020230076.1:1-12903	NA	NA	NA	NA	NA	8637-5947	5953-5054	8637-5947	12159-12902, 5156-3450	5156-3450	FALSE		
harpegnathos_saltator_NW_020230076.1:1-13224	NA	NA	NA	NA	NA	8637-5947	5953-5054	8637-5947	12159-13010, 5156-3450	12159-13010, 5156-3450	FALSE		
harpegnathos_saltator_NW_020230098.1:75562-96947	2889:3541	12599:13251	2885:2888/13252:13255	12584:12598	NA	4944-5615	6004-8073, 16431-15874	6004-8073	23-1015, 8121-9272	23-1015, 8121-9272	FALSE		
harpegnathos_saltator_NW_020230104.1:1-14346	NA	NA	NA	NA	NA	10511-9798	9446-7281	9446-7281	7329-6178	NA	FALSE		
harpegnathos_saltator_NW_020230119.1:1-15320	NA	NA	NA	NA	NA	NA	1003-1608	11158-11517, 11527-12771	2156-2566	NA	FALSE		
harpegnathos_saltator_NW_020230121.1:7936-26579	NA	NA	NA	NA	NA	2263-827	6400-8121, 18309-18644, 2263-827, 625-2	5568-6323, 6400-8121, 18309-18644, 2263-827	8481-8987, 625-2	8975-9709, 13636-13986	FALSE		
harpegnathos_saltator_NW_020230125.1:1-15299	NA	NA	NA	NA	NA	NA	NA	NA	NA	865-1395	FALSE		
harpegnathos_saltator_NW_020230140.1:66716-88173	1137:2385	12497:13745	1133:1136/13746:13749	12482:12496	NA	3977-4552	4637-9421	4637-9421	4637-9421	4637-9421, 19353-18583	FALSE		
harpegnathos_saltator_NW_020230144.1:1-12156	NA	NA	NA	NA	NA	7569-7111	4822-4463	7024-4889	4448-2382	4448-2382	FALSE		
harpegnathos_saltator_NW_020230150.1:1-12922	NA	NA	NA	NA	NA	NA	6518-4923	7355-6528, 6518-4923	4914-3187	4914-3187	FALSE		
harpegnathos_saltator_NW_020230159.1:19375-40058	NA	NA	NA	NA	NA	5045-5914	7045-7452	6110-6751	8456-9385	NA	FALSE		
harpegnathos_saltator_NW_020230163.1:295360-313191	NA	NA	NA	NA	NA	436-1131	14448-13510	15646-14966, 15009-14542	1465-2553, 13491-12178, 4780-3893	13491-12178, 4780-3893	FALSE		
harpegnathos_saltator_NW_020230163.1:296413-313191	NA	NA	NA	NA	NA	NA	13395-12457	14593-13913, 13956-13489	412-1500, 12438-11125, 3727-2840	12438-11125, 3727-2840	FALSE		
harpegnathos_saltator_NW_020230163.1:76064-96792	NA	NA	NA	NA	NA	NA	7592-9775	NA	7592-9775, 17763-17380	7592-9775, 18321-17842	FALSE		

Sequence	LTR5	LTR3	TSR	PPT	pos_gag	pos_protease	pos_rnase	pos_rt	pos_integrase	pos_transposase	complete	TE	Structure
harpegnathos_saltator_NW_020230166.1:255575-277092	NA	NA	NA	NA	NA	18013-16397	16333-15554, 15517-14213	18013-16397, 16333-15554	15517-14213	NA	FALSE		
harpegnathos_saltator_NW_020230176.1:2817-24277	6369:8272	18797:20700	6365:6368/20701:20704	8273:8287	NA	16801-15836	14214-13768, 13749-12205	15257-14208, 14214-13768	13749-12205, 5721-5014	13749-12205, 5721-5014	FALSE		
harpegnathos_saltator_NW_020230176.1:3018-23962	6168:8071	18596:20499	6164:6167/20500:20503	8072:8086	NA	16600-15635	14013-13567, 13548-12004	15056-14007, 14013-13567	13548-12004, 5520-4813	13548-12004, 5520-4813	FALSE		
harpegnathos_saltator_NW_020230194.1:208097-228822	NA	NA	NA	NA	NA	NA	7196-8413	NA	18234-17389, 11858-10812	11858-10812	FALSE		
harpegnathos_saltator_NW_020230195.1:63438-84163	NA	NA	NA	NA	NA	NA	12465-11971	12991-12653	14329-14886, 16981-16526, 15675-15079, 9562-8762	14329-14886, 15675-15079, 9562-8762	FALSE		
harpegnathos_saltator_NW_020230200.1:224132-236568	NA	NA	NA	NA	NA	NA	NA	NA	1729-1274	NA	FALSE		
harpegnathos_saltator_NW_020230206.1:201876-216209	2963:4093	12311:13472	NOTFOUND	12266:12280	NA	5691-9692	5691-9692	5691-9692	5691-9692	5691-9692	FALSE		
harpegnathos_saltator_NW_020230212.1:137266-151580	NA	NA	NA	NA	NA	NA	13559-12891	NA	6186-5497, 536-9	11785-10955, 6543-6202	FALSE		
harpegnathos_saltator_NW_020230224.1:9-18143	NA	NA	NA	NA	NA	17126-16737	14608-12008	15650-15132, 15201-14602	14608-12008	14608-12008	FALSE		
harpegnathos_saltator_NW_020230229.1:170048-186793	NA	NA	NA	NA	NA	NA	14470-12029	15395-14568	14470-12029	6610-7659, 14470-12029	FALSE		
harpegnathos_saltator_NW_020230234.1:1-16464	NA	NA	NA	NA	NA	NA	10178-7929	10178-7929	16156-16464, 10178-7929	7925-7011	FALSE		
harpegnathos_saltator_NW_020230243.1:163997-185334	NA	NA	NA	NA	NA	4978-5988	6912-7604	4978-5988, 17509-17958	7863-8384, 2336-1950, 2019-1672	2019-1672	FALSE		
harpegnathos_saltator_NW_020230253.1:81074-93940	NA	NA	NA	NA	NA	NA	NA	NA	12845-11757	12845-11757	FALSE		
harpegnathos_saltator_NW_020230291.1:1-12133	NA	NA	NA	NA	NA	NA	5672-2826	6634-5672, 5672-2826	5672-2826	NA	FALSE		
harpegnathos_saltator_NW_020230314.1:1-15926	NA	NA	NA	NA	NA	NA	NA	NA	2409-4169	2409-4169, 8597-9190	FALSE		
harpegnathos_saltator_NW_020230314.1:1224-22915	NA	NA	NA	NA	NA	NA	NA	NA	1186-2946	1186-2946, 7374-7967	FALSE		
harpegnathos_saltator_NW_020230316.1:119983-140711	NA	NA	NA	NA	NA	4590-5930	7338-8144	6135-6731, 6777-7256	8291-9775, 14949-15275	8291-9775, 14949-15275	FALSE		
harpegnathos_saltator_NW_020230316.1:120304-141764	NA	NA	NA	NA	NA	4269-5609	7017-7823	5814-6410, 6456-6935	7970-9454, 14628-14954	7970-9454, 14628-14954, 21343-20522	FALSE		
harpegnathos_saltator_NW_020230316.1:614844-635572	NA	NA	NA	NA	NA	20298-20729	7619-8134	6127-6903, 19330-19731	8195-8893, 16062-15058, 1658-1164	8820-9608, 16062-15058	FALSE		
harpegnathos_saltator_NW_020230316.1:847739-868464	NA	NA	NA	NA	NA	NA	14322-12727	16425-14332, 14322-12727	12718-10991	12718-10991	FALSE		
harpegnathos_saltator_NW_020230318.1:1-16461	NA	NA	NA	NA	NA	NA	51-914	51-914	862-2736	862-2736	FALSE		
harpegnathos_saltator_NW_020230338.1:1-16607	NA	NA	NA	NA	NA	NA	613-1233	15-623	1258-2889	1258-2889, 16493-15594	FALSE		
harpegnathos_saltator_NW_020230345.1:192595-214322	NA	NA	NA	NA	NA	NA	5752-7824, 16784-17179	NA	5752-7824, 17471-18151	NA	FALSE		
harpegnathos_saltator_NW_020230345.1:193009-213929	NA	NA	NA	NA	NA	NA	5338-7410, 16370-16765	NA	5338-7410, 17057-17737	NA	FALSE		
harpegnathos_saltator_NW_020230369.1:1-15863	NA	NA	NA	NA	NA	NA	2709-6353	2709-6353	2709-6353, 12992-12534	2709-6353, 13580-13056, 12992-12534	FALSE		
harpegnathos_saltator_NW_020230381.1:1-10656	NA	NA	NA	NA	NA	NA	4970-2007	4970-2007	NA	1999-965	FALSE		
harpegnathos_saltator_NW_020230394.1:25562-45620	7334:7543	19818:20029	NOTFOUND	7628:7642	NA	NA	15158-13110	15158-13110	12131-11460	12131-11460	FALSE		
harpegnathos_saltator_NW_020230399.1:1-13877	NA	NA	NA	NA	NA	NA	5616-5122	6142-5804	7476-8033, 10129-9674, 9272-8226, 2335-1940	7476-8033, 9272-8226, 2335-1940	FALSE		
harpegnathos_saltator_NW_020230402.1:1-17758	8579:8871	13811:14103	8573:8578/14104:14109	13796:13810	NA	9901-13083	9901-13083	9901-13083	9901-13083, 16620-17078	9901-13083, 16620-17078	FALSE		
harpegnathos_saltator_NW_020230411.1:26907-48367	6484:8342	19180:21052	NOTFOUND	8343:8357	NA	17184-16726	15386-13254	15386-13254	13233-12025	13233-12025, 1324-863	FALSE		

Sequence	LTR5	LTR3	TSR	PPT	pos_gag	pos_protease	pos_rnase	pos_rt	pos_integrase	pos_transposase	complete	TE	Structure
harpegnathos_saltator_NW_020230415.1:1410245-1430970	NA	NA	NA	NA	NA	NA	NA	853-1278	11471-10806, 6401-6069	11471-10806	FALSE		
harpegnathos_saltator_NW_020230418.1:1-13509	NA	NA	NA	NA	NA	10129-9572	7828-6911	7828-6911	4850-3735	4850-3735	FALSE		
harpegnathos_saltator_NW_020230423.1:94422-115906	NA	NA	NA	NA	NA	18628-18044	15614-14835	16443-15838	14875-14093	NA	FALSE		
harpegnathos_saltator_NW_020230461.1:1-20167	NA	NA	NA	NA	NA	NA	4304-5605, 5633-6628	4304-5605	6550-7368, 14103-13603	7396-7827	FALSE		
harpegnathos_saltator_NW_020230463.1:2868-17647	NA	NA	NA	NA	NA	3537-7967	3537-7967	3537-7967	3537-7967	3537-7967	FALSE		
harpegnathos_saltator_NW_020230471.1:62210-83901	NA	NA	NA	NA	2083-761	709-2	NA	NA	NA	NA	FALSE		
harpegnathos_saltator_NW_020230484.1:15975-30417	NA	NA	NA	NA	NA	3742-7485	3742-7485	3742-7485	3742-7485	7514-8173	FALSE		
harpegnathos_saltator_NW_020230484.1:16179-30417	NA	NA	NA	NA	NA	3538-7281	3538-7281	3538-7281	3538-7281	7310-7969	FALSE		
harpegnathos_saltator_NW_020230493.1:381240-402655	NA	NA	NA	NA	377-1549	1450-2073	2646-3227, 3240-3605, 14812-14009	2104-2718, 2646-3227, 18499-17447, 15355-14858	3699-4667, 7623-8720, 13975-12839	7623-8720, 12817-11999	FALSE		
harpegnathos_saltator_NW_020230493.1:49006-70502	NA	NA	NA	NA	NA	NA	14626-13427	16696-15311, 15157-14660	NA	13411-12056	FALSE		
harpegnathos_saltator_NW_020230500.1:27083-42440	NA	NA	NA	NA	NA	NA	14850-12007	15172-14804, 14850-12007	63-392, 14850-12007	63-392, 14850-12007	FALSE		
harpegnathos_saltator_NW_020230519.1:139179-160870	NA	NA	NA	NA	NA	18156-13726	18156-13726	18156-13726	18156-13726	18156-13726	FALSE		
harpegnathos_saltator_NW_020230519.1:170990-191712	2535:4094	12247:13828	NOTFOUND	12215:12229	NA	1473-2225, 5692-9693	5692-9693	5692-9693	5692-9693	5692-9693	FALSE		
harpegnathos_saltator_NW_020230519.1:176590-195243	8244:9883	16952:18590	8240:8243/18591:18594	16897:16911	NA	92-4093	92-4093	92-4093	92-4093	92-4093	FALSE		
harpegnathos_saltator_NW_020230528.1:733301-754764	NA	NA	NA	NA	NA	19807-18263	16615-15560, 15517-15146	16615-15560	142-732, 2431-3099, 17732-16644, 13477-12011	1836-2318, 17732-16644, 13477-12011	FALSE		
harpegnathos_saltator_NW_020230528.1:734357-755085	NA	NA	NA	NA	NA	18751-17207	15559-14504, 14461-14090	15559-14504	1375-2043, 16676-15588, 12421-10955	780-1262, 16676-15588, 12421-10955	FALSE		
harpegnathos_saltator_NW_020230551.1:125995-140722	2965:3618	12667:13338	2961:2964/13339:13342	12652:12666	NA	5048-5761	6725-7885	6113-6466	7943-8560, 8529-9380	NA	FALSE		
harpegnathos_saltator_NW_020230556.1:89736-111226	NA	NA	NA	NA	NA	NA	14929-13013	15784-14936, 14929-13013	1364-1717, 8054-9100, 7422-6619	979-1593, 8054-9100, 12826-12497, 7422-6619	FALSE		
harpegnathos_saltator_NW_020230556.1:90027-110965	NA	NA	NA	NA	NA	NA	14638-12722	15493-14645, 14638-12722	1073-1426, 7763-8809, 7131-6328	688-1302, 7763-8809, 12535-12206, 7131-6328	FALSE		
harpegnathos_saltator_NW_020230560.1:663929-678664	699:2022	13400:14724	NOTFOUND	13385:13399	NA	4584-5675	6609-7640, 7619-9775	6609-7640	7619-9775	7619-9775	FALSE		
harpegnathos_saltator_NW_020230560.1:664250-678664	378:1701	13079:14403	NOTFOUND	13064:13078	NA	4263-5354	6288-7319, 7298-9454	6288-7319	7298-9454	7298-9454	FALSE		
harpegnathos_saltator_NW_020230571.1:1-12981	NA	NA	NA	NA	NA	NA	10359-9658	12128-10389	8992-7805	NA	FALSE		
harpegnathos_saltator_NW_020230571.1:1-13059	NA	NA	NA	NA	NA	NA	10359-9658	12128-10389	8992-7805	NA	FALSE		
harpegnathos_saltator_NW_020230577.1:1-13014	NA	NA	NA	NA	NA	8742-6682	6746-3561	8742-6682, 6746-3561	6746-3561	6746-3561	FALSE		
harpegnathos_saltator_NW_020230579.1:1-13541	NA	NA	NA	NA	NA	NA	NA	NA	NA	352-1035	FALSE		
harpegnathos_saltator_NW_020230579.1:1-14759	NA	NA	NA	NA	NA	NA	NA	NA	NA	352-1035	FALSE		
harpegnathos_saltator_NW_020230582.1:867030-888487	NA	NA	NA	NA	NA	NA	16596-16291	17242-16688, 16596-16291	24-830, 7570-6731, 4218-3322	24-830, 7757-7443, 7570-6731	FALSE		
harpegnathos_saltator_NW_020230585.1:78731-99459	NA	NA	NA	NA	NA	NA	NA	NA	8830-9267	NA	FALSE		
harpegnathos_saltator_NW_020230596.1:31975-52697	3410:3653, 3485:3653	12521:12711, 12521:12786	NOTFOUND	12472:12486	NA	NA	NA	6370-8064	8202-9098	NA	FALSE		
harpegnathos_saltator_NW_020230607.1:108221-122456	NA	NA	NA	NA	NA	NA	14175-13624	NA	13378-12008	13378-12008	FALSE		
harpegnathos_saltator_NW_020230607.1:109274-122456	NA	NA	NA	NA	NA	NA	13122-12571	NA	12325-10955	12325-10955	FALSE		
harpegnathos_saltator_NW_020230610.1:23315-44712	3658:3949	8888:9180	3654:3657/9181:9184	3950:3964	NA	7857-4840	7857-4840	7857-4840	7857-4840, 2947-2609	1062-1415, 1721-2098, 7857-4840	FALSE		

Sequence	LTR5	LTR3	TSR	PPT	pos_gag	pos_protease	pos_rnase	pos_rt	pos_integrase	pos_transposase	complete	TE	Structure
harpegnathos_saltator_NW_020230621.1:1-12576	NA	NA	NA	NA	NA	NA	NA	NA	469-1545	469-1545	FALSE		
harpegnathos_saltator_NW_020230623.1:29116-44079	NA	NA	NA	NA	NA	NA	NA	NA	14914-14411	14531-13725	FALSE		
harpegnathos_saltator_NW_020230626.1:5441-27057	NA	NA	NA	NA	NA	1563-2831	2774-5953	2774-5953	2774-5953	2774-5953	FALSE		
harpegnathos_saltator_NW_020230643.1:1-13992	5080:5364	9701:9985	5074:5079/9986:9991	5365:5379	NA	9270-5881	132-2174, 9270-5881	9270-5881	132-2174, 9270-5881	132-2174, 9270-5881	FALSE		
harpegnathos_saltator_NW_020230665.1:1-19796	NA	NA	NA	NA	NA	NA	4285-4815	3899-4291	6875-7990, 18057-17641	6875-7990, 19225-19794	FALSE		
harpegnathos_saltator_NW_020230700.1:1574255-1590046	NA	NA	NA	NA	NA	NA	14448-14089	14898-14515	14074-12008	14074-12008	FALSE		
harpegnathos_saltator_NW_020230700.1:1575308-1590046	NA	NA	NA	NA	NA	NA	13395-13036	13845-13462	13021-10955	13021-10955	FALSE		
heligmosomoides_polygyrus_FMJQ01000052.1:340083-362053	NA	NA	NA	NA	NA	NA	NA	21589-21969	13403-14170, 17705-18166	17705-18166	FALSE		
lasius_niger_LBMM01004455.1:1-14290	NA	NA	NA	NA	NA	3280-5574	3280-5574	3280-5574	5763-6926	NA	FALSE		
lasius_niger_LBMM01007617.1:1-8448	NA	NA	NA	NA	NA	NA	13-2625	13-2625	13-2625	13-2625	FALSE		
lasius_niger_LBMM01008064.1:1-7907	NA	NA	NA	NA	NA	1600-5568	1600-5568	1600-5568	1600-5568	1600-5568	FALSE		
lasius_niger_LBMM01008323.1:1-7613	NA	NA	NA	NA	NA	NA	7610-5907	NA	7610-5907	7610-5907	FALSE		
lasius_niger_LBMM01009292.1:1-6629	NA	NA	NA	NA	NA	NA	4-2175	NA	4-2175	4-2175	FALSE		
lasius_niger_LBMM01011353.1:1-5137	NA	NA	NA	NA	NA	NA	NA	NA	NA	5114-4716	FALSE		
lasius_niger_LBMM01012068.1:1-4741	NA	NA	NA	NA	NA	NA	NA	NA	4666-2873	NA	FALSE		
leptinotarsa_decemlineata_NW_019291774.1:52358-72249	NA	NA	NA	NA	NA	17128-16334	NA	16019-15249	14937-13705	14937-13705	FALSE		
leptinotarsa_decemlineata_NW_019293082.1:6662-28632	NA	NA	NA	NA	NA	661-1194	2124-2756, 5779-6144	1210-1560, 1684-2019, 5420-5809, 5779-6144	NA	3083-3391	FALSE		
monomorium_pharaonis_NC_050467.1:14762714-14784741	NA	NA	NA	NA	NA	3175-7344	3175-7344, 21906-20176	3175-7344	3175-7344, 21906-20176	3175-7344, 15386-15955, 21906-20176	FALSE		
monomorium_pharaonis_NC_050467.1:15387722-15409533	NA	NA	NA	NA	NA	NA	2788-7287, 21807-18790	2788-7287, 21807-18790	2788-7287, 21807-18790	2788-7287, 21807-18790	FALSE		
monomorium_pharaonis_NC_050467.1:15437447-15459573	NA	NA	NA	NA	NA	NA	17566-14822	17566-14822	17566-14822	17566-14822	FALSE		
monomorium_pharaonis_NC_050467.1:27696364-27718427	NA	NA	NA	NA	NA	NA	17657-13350	17657-13350	17657-13350	17657-13350	FALSE		
monomorium_pharaonis_NC_050467.1:30529541-30551568	NA	NA	NA	NA	NA	NA	NA	NA	2481-3014	19789-19046	FALSE		
monomorium_pharaonis_NC_050467.1:33747655-33769604	NA	NA	NA	NA	NA	NA	NA	NA	13782-11929	NA	FALSE		
monomorium_pharaonis_NC_050467.1:36550230-36571345	2715:2914	14368:14567	NOTFOUND	14353:14367	NA	NA	5735-8974	5735-8974, 17702-18592, 18630-19748	NA	9089-9751	FALSE		
monomorium_pharaonis_NC_050467.1:36657895-36679007	NA	NA	NA	NA	NA	NA	6580-8553	6580-8553	8633-9406	NA	FALSE		
monomorium_pharaonis_NC_050467.1:7386574-7408601	NA	NA	NA	NA	NA	18829-14660	18829-14660	18829-14660	18829-14660	18829-14660	FALSE		
monomorium_pharaonis_NC_050467.1:7876929-7898899	780:3392	14759:17372	776:779/17373:17376	14674:14688	NA	NA	5938-9981	5938-9981	5938-9981	5938-9981	FALSE		
monomorium_pharaonis_NC_050468.1:26162733-26184796	NA	NA	NA	NA	NA	NA	4408-8715	4408-8715	4408-8715	4408-8715	FALSE		
monomorium_pharaonis_NC_050468.1:28958273-28980300	NA	NA	NA	NA	NA	3196-7365	3196-7365	3196-7365	3196-7365	3196-7365	FALSE		
monomorium_pharaonis_NC_050469.1:16869508-16891634	NA	NA	NA	NA	NA	NA	2785-7284	2785-7284	2785-7284	2785-7284	FALSE		
monomorium_pharaonis_NC_050470.1:13413982-13436045	NA	NA	NA	NA	NA	17645-15954	16059-13339	17645-15954, 16059-13339	16059-13339	16059-13339	FALSE		
monomorium_pharaonis_NC_050470.1:19355462-19377432	NA	NA	NA	NA	20728-17828	20728-17828	NA	20728-17828	NA	NA	FALSE		
monomorium_pharaonis_NC_050470.1:19558027-19579997	614:3392	14715:17493	610:613/17494:17497	14630:14644	NA	NA	5002-9981	5002-9981	5002-9981	5002-9981	FALSE		
monomorium_pharaonis_NC_050471.1:15703300-15725363	NA	NA	NA	NA	NA	NA	6088-8715	4409-6100, 6088-8715	6088-8715	6088-8715	FALSE		
monomorium_pharaonis_NC_050471.1:17680608-17701567	6278:7397	17310:18425	6274:6277/18426:18429	7398:7412	NA	NA	15287-11184	15287-11184	15287-11184	NA	FALSE		
monomorium_pharaonis_NC_050471.1:21890839-21912701	NA	NA	NA	NA	NA	NA	14626-13637	15540-14683	21327-20950, 13659-12799	12799-12110	FALSE		
monomorium_pharaonis_NC_050472.1:14263688-14284803	NA	NA	NA	NA	NA	NA	NA	NA	9089-9928, 2308-1967	20498-19674	FALSE		
monomorium_pharaonis_NC_050472.1:14264957-14285448	NA	NA	NA	NA	NA	NA	NA	NA	7820-8659, 1039-698	19229-18405	FALSE		

Sequence	LTR5	LTR3	TSR	PPT	pos_gag	pos_protease	pos_rnase	pos_rt	pos_integrase	pos_transposase	complete	TE	Structure
monomorium_pharaonis_NC_050473.1:23068164-23090287	19630:19852	21792:22027	19626:19629/22028:22031	19908:19922	NA	NA	2802-7298, 18225-19127	2802-7298	2802-7298	2802-7298	FALSE		
monomorium_pharaonis_NC_050473.1:23069442-23089954	18352:18823, 18352:18574	20038:20509, 20276:20509	18348:18351/20510:20513	18879:18893, 18630:18644	NA	NA	1524-6020, 16947-17849	1524-6020	1524-6020	1524-6020	FALSE		
monomorium_pharaonis_NC_050474.1:14832849-14853358	NA	NA	NA	NA	NA	18171-16474	16429-14810	18171-16474	16429-14810	NA	FALSE		
monomorium_pharaonis_NC_050475.1:9448937-9470895	NA	NA	NA	NA	NA	16157-14901	14998-14381, 14339-13791	16157-14901, 14998-14381	13470-11938	20330-20683	FALSE		
monomorium_pharaonis_NC_050475.1:9476740-9498551	NA	NA	NA	NA	NA	NA	2818-7314	2818-7314	2818-7314	2818-7314	FALSE		
monomorium_pharaonis_NC_050476.1:9341906-9362439	NA	NA	NA	NA	NA	17496-18350	NA	NA	NA	NA	FALSE		
monomorium_pharaonis_NW_023415500.1:1-15000	NA	NA	NA	NA	NA	NA	8472-7420	8472-7420	7469-5286	NA	FALSE		
monomorium_pharaonis_NW_023415643.1:63505-84437	NA	NA	NA	NA	NA	NA	17779-14513	17779-14513	17779-14513	17779-14513	FALSE		
monomorium_pharaonis_NW_023415798.1:1-20727	NA	NA	NA	NA	NA	NA	2771-7267	2771-7267	2771-7267, 12778-11696	2771-7267	FALSE		
monomorium_pharaonis_NW_023415798.1:21424-43316	NA	NA	NA	NA	NA	NA	NA	3811-4149	NA	21842-20940	FALSE		
monomorium_pharaonis_NW_023415798.1:30504-52396	NA	NA	NA	NA	NA	20396-21892	15180-11860	20396-21892, 15180-11860	15180-11860	NA	FALSE		
monomorium_pharaonis_NW_023415798.1:45056-65665	NA	NA	NA	NA	NA	5844-10034	5844-10034	5844-10034	5844-10034	NA	FALSE		
monomorium_pharaonis_NW_023415798.1:46337-65665	NA	NA	NA	NA	NA	4563-8753	4563-8753	4563-8753	4563-8753	NA	FALSE		
monomorium_pharaonis_NW_023415829.1:26074-48044	NA	NA	NA	NA	NA	16128-11938	16128-11938	16128-11938	16128-11938	NA	FALSE		
monomorium_pharaonis_NW_023415829.1:35357-56442	NA	NA	NA	NA	NA	6845-2655	6845-2655	6845-2655	6845-2655	NA	FALSE		
monomorium_pharaonis_NW_023415834.1:44008-66134	NA	NA	NA	NA	NA	NA	18862-14816	18862-14816	18862-14816	18862-14816	FALSE		
monomorium_pharaonis_NW_023415910.1:1-16946	NA	NA	NA	NA	NA	NA	12282-11245	14146-12188, 12282-11245	10678-9650	10678-9650	FALSE		
myzus_persicae_NW_019100660.1:666-22465	NA	NA	NA	NA	NA	NA	NA	NA	NA	1172-1558, 1659-2045, 20242-19445, 9595-9065	FALSE		
myzus_persicae_NW_019100944.1:9246-21278	NA	NA	NA	NA	NA	NA	NA	NA	7977-8708	7977-8708	FALSE		
nasonia_vitripennis_NC_045757.1:5910918-5932150	NA	NA	NA	NA	NA	NA	7267-8319	6709-7221, 7267-8319	8580-9227	NA	FALSE		
nasonia_vitripennis_NC_045758.1:18185757-18206944	NA	NA	NA	NA	NA	4611-7640	4611-7640	4611-7640, 14977-15501	NA	8350-9303	FALSE		
nasonia_vitripennis_NC_045760.1:29070475-29091716	376:2372	11576:13572	372:375/13573:13576	11499:11513	NA	5273-11338	5273-11338	5273-11338	5273-11338	NA	FALSE		
nasonia_vitripennis_NW_022279588.1:2525574-2546731	NA	NA	NA	NA	NA	NA	14297-13854	NA	NA	NA	FALSE		
nasonia_vitripennis_NW_022279591.1:4011282-4032484	NA	NA	NA	NA	NA	19185-19565	20787-21203	7474-8145, 19599-20456	8981-10438	NA	FALSE		
nasonia_vitripennis_NW_022279599.1:38911-60110	NA	NA	NA	NA	NA	NA	NA	NA	NA	2972-3394	FALSE		
nasonia_vitripennis_NW_022279601.1:324881-346035	1222:1614	11862:12259	1218:1221/12260:12263	11774:11788	NA	4832-5839	7174-9333, 13623-14612	5864-7081, 13064-13783	7174-9333	7174-9333	FALSE		
nasonia_vitripennis_NW_022279605.1:1071613-1092812	NA	NA	NA	NA	NA	NA	5317-8154	5317-8154, 1220-3	5317-8154	NA	FALSE		
nasonia_vitripennis_NW_022279605.1:284908-306113	NA	NA	NA	NA	NA	16479-15925	20979-19498, 14426-13827	20979-19498	13413-13054	13017-12145	FALSE		
nasonia_vitripennis_NW_022279608.1:2229318-2250487	NA	NA	NA	NA	NA	NA	58-1581	15767-16636, 19509-20681	14846-14412	58-1581, 5023-6036, 14396-13944	FALSE		
nasonia_vitripennis_NW_022279608.1:223060-244319	NA	NA	NA	NA	NA	NA	NA	5153-5533, 11655-12515	8645-8989	NA	FALSE		
nasonia_vitripennis_NW_022279608.1:3186817-3208064	NA	NA	NA	NA	NA	16555-14372	14362-13598	16555-14372	13224-12739	NA	FALSE		
nasonia_vitripennis_NW_022279610.1:2091634-2112887	NA	NA	NA	NA	NA	NA	NA	5917-5495	NA	NA	FALSE		
nasonia_vitripennis_NW_022279610.1:2292842-2314008	NA	NA	NA	NA	18058-15383	NA	15235-13958	15235-13958	21117-20692	18058-15383	FALSE		
nasonia_vitripennis_NW_022279613.1:423011-444411	NA	NA	NA	NA	NA	NA	7417-8010, 14431-13976	15401-14463	13838-13488	NA	FALSE		
nasonia_vitripennis_NW_022279625.1:1-19981	NA	NA	NA	NA	NA	NA	NA	NA	NA	14624-13188	FALSE		

Sequence	LTR5	LTR3	TSR	PPT	pos_gag	pos_protease	pos_rnase	pos_rt	pos_integrase	pos_transposase	complete	TE	Structure
nasonia_vitripennis_NW_022279625.1:812064-833230	NA	NA	NA	NA	NA	21054-16729	2787-3467, 21054-16729	1963-2703, 2787-3467, 21054-16729	3729-5117, 21054-16729	21054-16729	FALSE		
nasonia_vitripennis_NW_022279626.1:3104615-3125766	NA	NA	NA	NA	NA	NA	6867-7958	5571-6434	NA	NA	FALSE		
nasonia_vitripennis_NW_022279630.1:550931-572202	9012:9409	19898:20307	9008:9011/20308:20311	9483:9497	NA	16414-11918	16414-11918	16414-11918	16414-11918	NA	FALSE		
nasonia_vitripennis_NW_022279636.1:85436-106701	NA	NA	NA	NA	NA	16730-16062	14990-14289	NA	13084-12386	NA	FALSE		
nasonia_vitripennis_NW_022279638.1:2080894-2102117	NA	NA	NA	NA	NA	NA	21200-20349	5277-6878, 21200-20349	19257-18784	NA	FALSE		
nasonia_vitripennis_NW_022279639.1:1954181-1975446	9011:9408	19850:20247	9007:9010/20248:20251	9482:9496	NA	16443-14431	14016-13006	389-688, 16443-14431	12888-12349	5668-4472	FALSE		
nasonia_vitripennis_NW_022279643.1:2955304-2976500	NA	NA	NA	NA	NA	NA	NA	NA	12993-12667	12605-11811	FALSE		
nasonia_vitripennis_NW_022279643.1:644771-665886	NA	NA	NA	NA	NA	NA	NA	4877-5440, 5591-6301	NA	NA	FALSE		
nasonia_vitripennis_NW_022279643.1:957368-978582	NA	NA	NA	NA	NA	15478-14750	NA	NA	NA	13006-12350	FALSE		
nasonia_vitripennis_NW_022279644.1:348229-369371	NA	NA	NA	NA	NA	16450-15332	14664-13624	15389-14670, 14664-13624	3858-4934	12742-12284	FALSE		
nasonia_vitripennis_NW_022279652.1:1046253-1067518	NA	NA	NA	NA	NA	4819-6384	6362-8662, 15870-16301	6362-8662, 19691-19389	6362-8662	NA	FALSE		
nasonia_vitripennis_NW_022279655.1:658175-679284	NA	NA	NA	NA	NA	NA	NA	8955-8509	NA	1679-3	FALSE		
nasonia_vitripennis_NW_022279660.1:196426-217619	NA	NA	NA	NA	NA	NA	18461-17079	18461-17079	7607-8443, 15197-14205	8434-9282	FALSE		
nasonia_vitripennis_NW_022279661.1:166949-188196	299:1863	11535:13097	295:298/13098:13101	1936:1950	NA	4779-9350	4779-9350	4779-9350, 15460-14207	4779-9350	4779-9350	FALSE		
nasonia_vitripennis_NW_022279665.1:97411-118616	1347:2684	15885:17210, 18998:20323	1343:1346/17211:17214, 1343:1346/20324:20327	2691:2705	NA	4673-6433	6418-9141	6418-9141	NA	NA	FALSE		
nasonia_vitripennis_NW_022279684.1:43770-65017	4024:5390	16540:17917	4020:4023/17918:17921	16519:16533	NA	NA	NA	NA	NA	20859-20206	FALSE		
nasonia_vitripennis_NW_022279685.1:79223-100410	NA	NA	NA	NA	NA	15975-13438	15975-13438, 13344-12334	15975-13438	12349-11720	12349-11720	FALSE		
nasonia_vitripennis_NW_022279846.1:17935-37341	NA	NA	NA	NA	NA	NA	NA	NA	NA	15316-14399	FALSE		
odontomachus_brunneus_NW_022639451.1:8350559-8372469	NA	NA	NA	NA	NA	NA	NA	NA	15576-14425	14380-13670	FALSE		
odontomachus_brunneus_NW_022639451.1:8359666-8381474	NA	NA	NA	NA	NA	NA	21316-18179	21316-18179	21316-18179, 6469-5318	21316-18179	FALSE		
odontomachus_brunneus_NW_022639451.1:8378952-8400862	NA	NA	NA	NA	NA	NA	4382-3	4382-3	6676-5018	6676-5018	FALSE		
odontomachus_brunneus_NW_022639453.1:3400998-3422761	NA	NA	NA	NA	NA	NA	NA	NA	13175-12765, 12802-11957	NA	FALSE		
odontomachus_brunneus_NW_022639498.1:1995746-2017302	NA	NA	NA	NA	NA	NA	14453-13743	15401-15075, 15068-14700	13013-12699	8801-9100, 16387-18147, 12590-12171	FALSE		
odontomachus_brunneus_NW_022639505.1:295997-316533	NA	NA	NA	NA	NA	NA	NA	1130-744	16277-16642, 14182-13769	NA	FALSE		
odontomachus_brunneus_NW_022639507.1:840316-862202	NA	NA	NA	NA	NA	16322-12249	16322-12249	16322-12249	16322-12249	16322-12249	FALSE		
odontomachus_brunneus_NW_022639518.1:1-18356	NA	NA	NA	NA	NA	12667-10064	15336-17996, 12667-10064	12667-10064, 400-2	15336-17996, 9763-8492	15336-17996	FALSE		
odontomachus_brunneus_NW_022639518.1:9880-31799	NA	NA	NA	NA	NA	2788-185	5457-8117, 2788-185	2788-185	5457-8117	5457-8117	FALSE		
odontomachus_brunneus_NW_022639536.1:1466609-1487139	NA	NA	NA	NA	NA	NA	16736-14619	16736-14619	NA	NA	FALSE		
odontomachus_brunneus_NW_022639555.1:52774-74339	NA	NA	NA	NA	NA	NA	NA	NA	12944-12141, 8938-8264	8938-8264	FALSE		

Sequence	LTR5	LTR3	TSR	PPT	pos_gag	pos_protease	pos_rnase	pos_rt	pos_integrase	pos_transposase	complete	TE	Structure
odontomachus_brunneus_NW_022639555.1:739702-760982	NA	NA	NA	NA	NA	NA	NA	NA	2410-1982	2410-1982	FALSE		
odontomachus_brunneus_NW_022639561.1:172762-194675	NA	NA	NA	NA	18248-17322	4981-5523, 18248-17322	17376-15892	6251-6838, 6947-7279, 17376-15892	15862-13325	NA	FALSE		
odontomachus_brunneus_NW_022639561.1:32801-54618	NA	NA	NA	NA	NA	21382-16811	21382-16811	21382-16811	21382-16811	21382-16811	FALSE		
odontomachus_brunneus_NW_022639566.1:404389-424883	NA	NA	NA	NA	NA	1716-2072	2829-6230	2241-2822, 2829-6230, 20400-19984	2829-6230	NA	FALSE		
odontomachus_brunneus_NW_022639595.1:1-14665	NA	NA	NA	NA	NA	NA	10810-11343	9382-9828, 10810-11343	NA	NA	FALSE		
odontomachus_brunneus_NW_022639618.1:49485-70249	NA	NA	NA	NA	NA	NA	NA	5573-5100	6677-7192, 7812-8831	NA	FALSE		
odontomachus_brunneus_NW_022639618.1:749319-771229	NA	NA	NA	NA	NA	NA	16137-15226	17119-16184	15298-13649, 7395-7096	15298-13649	FALSE		
odontomachus_brunneus_NW_022639620.1:602546-624438	NA	NA	NA	NA	NA	16958-16038	15913-13781	15913-13781	NA	NA	FALSE		
odontomachus_brunneus_NW_022639620.1:619913-641733	NA	NA	NA	NA	NA	20206-20619, 18818-17946	16831-15794	17946-16831	15295-14966	14973-14029	FALSE		
odontomachus_brunneus_NW_022639624.1:664321-685964	NA	NA	NA	NA	NA	1978-5070	1978-5070	1978-5070	NA	NA	FALSE		
odontomachus_brunneus_NW_022639624.1:673821-695731	NA	NA	NA	NA	NA	NA	NA	19166-19636	NA	NA	FALSE		
odontomachus_brunneus_NW_022639624.1:728147-747103	NA	NA	NA	NA	NA	NA	NA	NA	2520-3578	2520-3578	FALSE		
odontomachus_brunneus_NW_022639642.1:93943-115655	NA	NA	NA	NA	NA	NA	6866-7693, 7778-8140, 1372-170	6866-7693, 3202-1376	8242-8844, 8860-9777, 16365-16994, 19046-19747, 1372-170	NA	FALSE		
odontomachus_brunneus_NW_022639661.1:277997-298596	NA	NA	NA	NA	NA	20186-19368	16240-13802	16240-13802	13700-11421	11399-10587	FALSE		
odontomachus_brunneus_NW_022639669.1:72066-92686	NA	NA	NA	NA	NA	5001-5885	NA	NA	NA	NA	FALSE		
odontomachus_brunneus_NW_022639673.1:378199-398798	NA	NA	NA	NA	NA	NA	6024-7976	6024-7976	8566-9432, 15847-15140	8566-9432	FALSE		
odontomachus_brunneus_NW_022639675.1:439560-461110	9539:9817	11195:11473	9535:9538/11474:11477	9853:9867	NA	NA	NA	NA	12058-9977	NA	FALSE		
odontomachus_brunneus_NW_022639676.1:72725-94497	NA	NA	NA	NA	NA	3709-6393	3709-6393	3709-6393, 20346-16975	NA	NA	FALSE		
odontomachus_brunneus_NW_022639695.1:289284-311281	NA	NA	NA	NA	NA	20296-18836	14357-12411	14357-12411	3778-4587, 2013-1606	3778-4587	FALSE		
odontomachus_brunneus_NW_022639696.1:178607-200424	NA	NA	NA	NA	NA	17254-15008	14942-14055, 14238-12202	21004-20537, 14942-14055	1984-2325, 14238-12202	14238-12202	FALSE		
odontomachus_brunneus_NW_022639702.1:360525-382423	NA	NA	NA	NA	NA	NA	NA	15383-14733, 14598-13702	2927-2385, 2475-2173	NA	FALSE		
odontomachus_brunneus_NW_022639713.1:1741-33828	NA	NA	NA	NA	NA	NA	27410-29800	27410-29800	5297-6211, 27410-29800, 17810-17133	29757-30659	FALSE		
odontomachus_brunneus_NW_022639713.1:35740-287791	NA	NA	NA	NA	NA	45681-46358, 240476-237921, 28029-27688	47485-48090, 48802-49698, 49500-50270, 137965-138408, 149007-149897, 159661-160308, 240476-237921, 237569-236289, 36246-35896, 22768-19067	40010-42397, 46881-47537, 48159-48794, 48802-49698, 134674-134985, 150140-150658, 158832-159371, 198453-201014, 213108-213683, 240476-237921, 105808-105308, 105470-104640, 97519-96671, 68126-66990, 22768-19067, 7621-6587, 6352-5168	49500-50270, 120974-122122, 150481-151230, 160319-162283, 236219-235092, 176856-176074, 170272-169967, 99282-98854, 22768-19067	120974-122122, 160319-162283, 99282-98854, 22768-19067	FALSE		

Sequence	LTR5	LTR3	TSR	PPT	pos_gag	pos_protease	pos_rnase	pos_rt	pos_integrase	pos_transposase	complete	TE	Structure
odontomachus_brunneus_NW_022639725.1:16613-182950	NA	NA	NA	NA	24590-21438	24590-21438, 20832-16279	104801-103632, 3913-2654	154709-156145, 106903-104861, 24590-21438, 20832-16279, 7081-6476	145333-144308, 35739-35224, 35217-34852, 27521-26883, 16131-15229, 2711-2358	39507-40502, 113635-117168, 145333-144308, 27521-26883, 2183-1878	FALSE		
odontomachus_brunneus_NW_022639725.1:184811-250824	NA	NA	NA	NA	NA	NA	NA	62864-61515, 61324-59864, 5731-5300	13048-13521, 51134-52159, 44225-41535, 36054-35749, 17289-16783	13048-13521, 51134-52159, 36414-36070	FALSE		
odontomachus_brunneus_NW_022639758.1:16980-67016	NA	NA	NA	NA	NA	NA	18729-22871, 3084-1786	18729-22871, 31463-32260, 32101-33237, 3084-1786	18729-22871, 1782-136	18729-22871	FALSE		
odontomachus_brunneus_NW_022639771.1:534-183913	53074:53538, 53823:54514, 115581:115857	64821:65283, 65063:65709, 121356:121632	NOTFOUND, 115577:115580/121633:121636	64806:64820, 64979:64993, 115858:115872	54839-55249, 174925-175626	55620-56816, 166229-167599, 175648-176064, 177388-177903	57054-57809, 166229-167599, 119385-118015	55620-56816, 151789-152727, 152687-153919, 167636-168124, 176829-177251, 120056-119445, 119385-118015, 69486-69124, 8867-8268	24023-24439, 57054-57809, 58126-62937, 101284-101880, 178433-178942, 155335-154991, 117575-117216, 20483-19326	117307-116807, 48327-48028, 47975-47364, 30793-30053, 20483-19326, 16710-16324	FALSE		
odontomachus_brunneus_NW_022639775.1:24330-159548	NA	NA	NA	NA	91774-94056, 70930-70346	33647-33066	7033-8274, 11244-12185, 17266-16958	6278-7084, 7033-8274, 11244-12185, 38512-36758, 32934-32539, 18358-17900, 17861-17556, 17266-16958	8852-9598, 54629-55693, 52704-52276	54629-55693, 91774-94056, 101111-102130, 52704-52276	FALSE		
odontomachus_brunneus_NW_022639779.1:160044-184894	NA	NA	NA	NA	NA	NA	5096-5587	4114-4719	5587-6909	6987-7580	FALSE		
odontomachus_brunneus_NW_022639783.1:13564-131780	60284:60770	70963:71448	60280:60283/71449:71452	60838:60852	NA	78408-80225	78408-80225	23108-24091, 31901-33676, 33725-34294, 56432-58078, 78408-80225, 89626-90498, 90861-91907, 9963-9457	94450-94857, 103489-103842, 65029-64703	93810-94388	FALSE		
odontomachus_brunneus_NW_022639792.1:43928-101282	NA	NA	NA	NA	NA	15906-14356	14858-14256	14858-14256	8171-7545	NA	FALSE		
odontomachus_brunneus_NW_022639821.1:51853-84182	NA	NA	NA	NA	NA	20348-21814, 4074-136	22312-23181, 17555-16350, 4074-136	20348-21814, 21735-22304, 17948-17559, 17555-16350, 4074-136	15113-14187, 4074-136	27254-27907, 15113-14187	FALSE		
odontomachus_brunneus_NW_022639839.1:7956-41960	NA	NA	NA	NA	NA	6405-5230	5216-4023	19940-20413, 6405-5230, 5216-4023	4183-2180	24597-25076, 29827-29450, 15732-15433, 4183-2180	FALSE		
odontomachus_brunneus_NW_022639844.1:11023-68247	NA	NA	NA	NA	NA	55147-55503, 34870-30707, 5444-4578	34870-30707, 3518-1908	34870-30707, 4556-3615	34870-30707, 3518-1908	34870-30707	FALSE		
odontomachus_brunneus_NW_022639904.1:2628-39030	NA	NA	NA	NA	NA	15362-15727, 10876-6713	10876-6713	10876-6713	10876-6713	18845-21574, 10876-6713	FALSE		
ooceraea_biroi_NC_039506.1:11348881-11370119	NA	NA	NA	NA	NA	NA	1804-2205, 4493-5497, 13583-12750	1046-1636, 14059-13595	12606-11509	3-1046, 12606-11509	FALSE		
ooceraea_biroi_NC_039506.1:11827169-11848896	NA	NA	NA	NA	NA	NA	18577-18104	19027-18596	5752-6645, 18104-17076	5752-6645, 6476-6784, 18104-17076	FALSE		
ooceraea_biroi_NC_039506.1:11837949-11859034	NA	NA	NA	NA	15922-16257	NA	7797-7324	8247-7816	11284-11931, 7324-6296	11284-11931, 7324-6296	FALSE		

Sequence	LTR5	LTR3	TSR	PPT	pos_gag	pos_protease	pos_rnase	pos_rt	pos_integrase	pos_transposase	complete	TE	Structure
ooceraea_biroi_NC_039507.1:11447594-11469066	NA	NA	NA	NA	NA	2679-3212	5635-6207, 15119-13809, 13687-12146	4406-4834, 15119-13809	13687-12146	NA	FALSE		
ooceraea_biroi_NC_039507.1:12274875-12296350	NA	NA	NA	NA	NA	16324-14288	14197-12002	4799-5173, 16324-14288, 14197-12002	14197-12002	NA	FALSE		
ooceraea_biroi_NC_039508.1:8291222-8312682	NA	NA	NA	NA	NA	NA	14832-13201	14832-13201	5851-6498, 13134-12706	12660-11896	FALSE		
ooceraea_biroi_NC_039508.1:9269104-9289988	NA	NA	NA	NA	NA	NA	14172-12325	15258-14572	14172-12325	NA	FALSE		
ooceraea_biroi_NC_039509.1:7582967-7604154	NA	NA	NA	NA	NA	NA	5116-8598	5116-8598	5116-8598	NA	FALSE		
ooceraea_biroi_NC_039510.1:7834312-7855610	NA	NA	NA	NA	NA	NA	4141-5181, 5310-7328	4141-5181	5310-7328	5310-7328	FALSE		
ooceraea_biroi_NC_039510.1:7940589-7961587	NA	NA	NA	NA	NA	NA	NA	NA	17211-18242	17211-18242, 19520-18948, 18914-18498	FALSE		
ooceraea_biroi_NC_039511.1:163901-184929	NA	NA	NA	NA	1367-627	NA	16787-15645	16787-15645	19351-20367	19351-20367, 14935-14258	FALSE		
ooceraea_biroi_NC_039511.1:164228-185685	NA	NA	NA	NA	1040-300	NA	16460-15318	16460-15318	19024-20040	19024-20040, 14608-13931	FALSE		
ooceraea_biroi_NC_039511.1:31217-53061	NA	NA	NA	NA	NA	NA	5964-9962	5964-9962	5964-9962	NA	FALSE		
ooceraea_biroi_NC_039512.1:13381039-13402265	NA	NA	NA	NA	NA	2415-6377, 16109-15141	2415-6377	2415-6377, 15271-14351	2415-6377, 13897-12647, 12553-11894	2415-6377	FALSE		
ooceraea_biroi_NC_039513.1:2268289-2290133	7808:9761	17440:19391	NOTFOUND	9845:9859	NA	NA	14751-11884	15044-14724, 14751-11884, 6340-6029	14751-11884	2813-2346	FALSE		
ooceraea_biroi_NC_039513.1:671496-693340	NA	NA	NA	NA	NA	NA	5964-9962, 21742-21065, 16086-14008	5964-9962, 21742-21065, 20074-19103, 16086-14008	5964-9962, 16086-14008	16086-14008	FALSE		
ooceraea_biroi_NC_039514.1:5269297-5290325	NA	NA	NA	NA	NA	NA	5386-7068, 7143-7445, 1594-314	5386-7068, 2063-1710, 1594-314	7889-9088, 1594-314, 410-3	NA	FALSE		
ooceraea_biroi_NC_039514.1:5378634-5400040	NA	NA	NA	NA	NA	NA	1767-2591	1111-1881, 1767-2591	2946-4076, 20270-20638	2946-4076, 17593-18456, 18333-18728	FALSE		
ooceraea_biroi_NC_039514.1:5395961-5417367	NA	NA	NA	NA	NA	NA	NA	NA	2943-3311	266-1129, 1006-1401, 3176-4072	FALSE		
ooceraea_biroi_NC_039515.1:798936-820069	NA	NA	NA	NA	18966-21026	18966-21026	NA	NA	363-845, 4188-4874, 18100-17579	4188-4874	FALSE		
ooceraea_biroi_NC_039515.1:9499359-9521203	NA	NA	NA	NA	NA	NA	NA	NA	19167-19718	NA	FALSE		
ooceraea_biroi_NC_039517.1:12981705-13003237	NA	NA	NA	NA	NA	NA	8067-8990	6517-6852	19876-20610	8965-9396	FALSE		
ooceraea_biroi_NC_039519.1:1822259-1843491	NA	NA	NA	NA	NA	NA	NA	NA	18360-17359	NA	FALSE		
ooceraea_biroi_NW_020825074.1:1-21728	NA	NA	NA	NA	NA	NA	7858-8202, 20160-16612, 7638-5737	20160-16612	8415-9845, 20160-16612, 7638-5737	NA	FALSE		
ooceraea_biroi_NW_020825074.1:18532-39013	NA	NA	NA	NA	NA	5358-8201	5358-8201	5358-8201	NA	NA	FALSE		
ooceraea_biroi_NW_020825076.1:1-11943	NA	NA	NA	NA	NA	NA	1341-2441, 11188-9446	1039-1341, 1341-2441, 11619-11092	2516-3946, 11188-9446	NA	FALSE		
ooceraea_biroi_NW_020825080.1:11149-32336	2028:2843	11658:12474	NOTFOUND	11560:11574	NA	NA	5543-7900, 17092-20589	5543-7900, 17092-20589	8110-9744, 17092-20589	NA	FALSE		
ooceraea_biroi_NW_020825080.1:21776-40906	8:1864	11058:12912	3:7/12913:12917	10984:10998	NA	NA	6465-9962	6465-9962	6465-9962	NA	FALSE		
ooceraea_biroi_NW_020825081.1:1-16336	NA	NA	NA	NA	NA	12652-13542	2253-4751, 13401-15128	2253-4751, 13401-15128	2253-4751	NA	FALSE		
ooceraea_biroi_NW_020825094.1:1-21228	NA	NA	NA	NA	NA	NA	NA	NA	NA	14050-13157	FALSE		
ooceraea_biroi_NW_020825120.1:12135-33163	NA	NA	NA	NA	NA	NA	13883-13407	NA	13304-11943	13304-11943	FALSE		
ooceraea_biroi_NW_020825123.1:1-15191	NA	NA	NA	NA	NA	NA	15011-13110	15011-13110	NA	NA	FALSE		
ooceraea_biroi_NW_020825123.1:1-19088	NA	NA	NA	NA	NA	16231-15341	15320-13110	15320-13110	NA	NA	FALSE		
ooceraea_biroi_NW_020825124.1:384-21412	NA	NA	NA	NA	NA	NA	6800-8089	5115-6728	NA	8155-9084	FALSE		
ooceraea_biroi_NW_020825129.1:1-17597	NA	NA	NA	NA	NA	NA	61-2997	61-2997	3051-4079	NA	FALSE		
ooceraea_biroi_NW_020825138.1:1-15075	NA	NA	NA	NA	NA	13871-15073	598-3192	598-3192, 13871-15073	598-3192	NA	FALSE		

Sequence	LTR5	LTR3	TSR	PPT	pos_gag	pos_protease	pos_rnase	pos_rt	pos_integrase	pos_transposase	complete	TE	Structure
ooceraea_biroi_NW_020825140.1:182-14448	NA	NA	NA	NA	NA	NA	14155-13064	NA	13009-12605, 12701-11901	NA	FALSE		
ooceraea_biroi_NW_020825155.1:4269-15506	NA	NA	NA	NA	NA	NA	NA	NA	2269-3288	2269-3288	FALSE		
osmia_lignaria_NW_023009276.1:422649-443371	NA	NA	NA	NA	14566-14039	NA	NA	20657-20049, 17512-16535, 16492-15890	NA	NA	FALSE		
osmia_lignaria_NW_023009287.1:448580-469302	NA	NA	NA	NA	593-1096	NA	NA	NA	NA	1716-2768, 18922-18128, 14687-14061	FALSE		
osmia_lignaria_NW_023009290.1:450903-471625	NA	NA	NA	NA	NA	NA	12840-10783	NA	12840-10783	12840-10783	FALSE		
osmia_lignaria_NW_023009291.1:1-12676	NA	NA	NA	NA	NA	7299-4156	7299-4156	7299-4156	4085-2736	4085-2736	FALSE		
osmia_lignaria_NW_023009291.1:400425-421081	NA	NA	NA	NA	NA	NA	6045-6353, 2602-905	5536-6069, 6045-6353, 2602-905	NA	713-33	FALSE		
osmia_lignaria_NW_023009298.1:209391-230113	NA	NA	NA	NA	2924-3427	7974-6733	NA	4033-5052, 5085-5600, 6739-6059	1682-1260	NA	FALSE		
osmia_lignaria_NW_023009300.1:20015-40767	NA	NA	NA	NA	NA	NA	7291-9972	6420-7223, 7291-9972	1085-1669, 7291-9972	1806-2168, 7291-9972	FALSE		
osmia_lignaria_NW_023009301.1:47531-68253	NA	NA	NA	NA	NA	7642-6767	5804-4917	6773-5823, 5804-4917	4826-3570	NA	FALSE		
osmia_lignaria_NW_023009302.1:121618-142340	4359:5215	17745:18601	4355:4358/18602:18605	5282:5296	NA	15427-14423, 602-174	14598-10783	14598-10783	14598-10783	14598-10783	FALSE		
osmia_lignaria_NW_023009307.1:171517-192239	13433:13903	19508:19978	13428:13432/19979:19983	19493:19507	13986-19205	13986-19205	13510-11540	1003-1986, 13986-19205, 20437-20084, 13510-11540	13986-19205, 13510-11540	238-786	FALSE		
osmia_lignaria_NW_023009314.1:1-10897	NA	NA	NA	NA	NA	NA	5745-5242	6995-6180, 6198-5776	5456-4770	NA	FALSE		
osmia_lignaria_NW_023009314.1:117871-138593	NA	NA	NA	NA	NA	NA	14925-11683, 7708-7295	14925-11683	14925-11683, 11634-10783, 7214-6834	11634-10783	FALSE		
osmia_lignaria_NW_023009315.1:65095-85817	NA	NA	NA	NA	NA	5378-6376	6840-9941	6840-9941	6840-9941	6840-9941	FALSE		
osmia_lignaria_NW_023009328.1:1-13133	NA	NA	NA	NA	NA	13064-12069	10868-9675	11888-10968	10868-9675, 4029-3193	4029-3193	FALSE		
osmia_lignaria_NW_023009330.1:3995-24693	NA	NA	NA	NA	17411-16851	NA	128-2419	NA	128-2419	128-2419, 16001-16306	FALSE		
osmia_lignaria_NW_023009332.1:92768-113520	NA	NA	NA	NA	NA	NA	14531-10782	14531-10782	20398-18989, 14531-10782	14531-10782	FALSE		
osmia_lignaria_NW_023009350.1:51488-72210	NA	NA	NA	NA	NA	5694-6836	6839-9184	6839-9184, 19720-18737	6839-9184	20485-19937	FALSE		
osmia_lignaria_NW_023009381.1:1-11405	NA	NA	NA	NA	NA	3176-7561	3176-7561	3176-7561	3176-7561	3176-7561	FALSE		
photinus_pyralis_NW_022170249.1:4480665-4502395	NA	NA	NA	NA	NA	NA	7370-8341	NA	7370-8341	8428-9528, 18038-18871	FALSE		
rhopalosiphum_maidis_NC_040877.1:46824282-46846234	8927:9523	17085:17681	8923:8926/17682:17685	9528:9542	NA	15734-12348	21901-21509, 15734-12348	15734-12348	21522-20251, 15734-12348	21522-20251, 5492-4455, 325-2	FALSE		
rhopalosiphum_maidis_NC_040877.1:46844280-46866241	9041:9554	18672:19215	NOTFOUND	9559:9573	NA	4040-3540	16735-13979, 2296-1511	16735-13979, 2945-2325	1524-253	20279-20698, 7146-5812, 1524-253	FALSE		
rhopalosiphum_maidis_NC_040877.1:47840604-47862373	8617:9474	17630:18487	8613:8616/18488:18491	9479:9493	NA	15475-12569	NA	15475-12569	NA	NA	FALSE		
rhopalosiphum_maidis_NC_040877.1:92857576-92879288	224:404	4173:4353, 5261:5441	218:223/4354:4359, 218:223/5442:5447	422:436	NA	NA	NA	NA	NA	NA	FALSE		

Sequence	LTR5	LTR3	TSR	PPT	pos_gag	pos_protease	pos_rnase	pos_rt	pos_integrase	pos_transposase	complete	TE	Structure
rhopalosiphum_maidis_NC_040877.1:92880502-92902454	25:1408, 25:1447, 25:1664, 40:1444, 40:1463, 45:1447, 45:1644, 45:1626, 45:1605, 64:1408, 64:1447, 79:1444, 79:1463, 103:1427, 3236:3832	20460:21937, 20381:21937, 20341:21937, 20167:21937, 20220:21855, 20220:21875, 20278:21914, 20283:21858, 20186:21878, 20283:21878, 20480:21937, 20401:21937, 20416:21855, 20416:21875, 20416:21914, 20460:21917, 12437:13033	20:24/21938:21942, 36:39/21856:21859, 36:39/21876:21879, 36:39/21915:21918, 39:44/21859:21864, 39:44/21879:21884, 59:63/21938:21942, 75:78/21856:21859, 75:78/21876:21879, 75:78/21915:21918, 97:102/21918:21923, 3232:3235/13034:13037	1464:1478, 1681:1695, 12418:12432	NA	5183-9607	5183-9607	5183-9607	5183-9607	NA	FALSE		
rhopalosiphum_maidis_NC_040877.1:94098479-94120431	3237:3832, 14195:16286	12437:13033, 19512:21901, 19512:21921	3233:3236/13034:13037, 14189:14194/21902:21907, 14189:14194/21922:21927	12418:12432, 19460:19474	NA	5183-9607	5183-9607	5183-9607	5183-9607	NA	FALSE		
rhopalosiphum_maidis_NC_040878.1:35055120-35076742	2367:2950	12060:12643	2363:2366/12644:12647	12041:12055	NA	3112-8820	3112-8820	3112-8820	3112-8820	NA	FALSE		
rhopalosiphum_maidis_NC_040879.1:12076943-12098607	NA	NA	NA	NA	NA	NA	13977-12619	NA	13977-12619	13977-12619	FALSE		
rhopalosiphum_maidis_NC_040879.1:40494618-40516570	6864:7460	12412:13008	6860:6863/13009:13012	12393:12407	NA	NA	NA	NA	8652-9608	NA	FALSE		
rhopalosiphum_maidis_NC_040879.1:60708057-60729283	NA	NA	NA	NA	NA	16268-15591	13304-12921	13304-12921	12691-12305, 12283-11720	NA	FALSE		
rhopalosiphum_maidis_NC_040879.1:61765758-61787533	NA	NA	NA	NA	NA	4934-5917	7592-8974	6912-7571, 7592-8974	7592-8974, 8943-9914	NA	FALSE		
rhopalosiphum_maidis_NC_040879.1:70379156-70401105	3234:3831	12475:13072	3230:3233/13073:13076	12456:12470	NA	5182-9606	5182-9606	5182-9606	5182-9606	NA	FALSE		

Sequence	LTR5	LTR3	TSR	PPT	pos_gag	pos_protease	pos_rnase	pos_rt	pos_integrase	pos_transposase	complete	TE	Structure
rhopalosiphum_maidis_NC_040879.1:72009723-72031675	8935:9531	18124:18720	8931:8934/18721:18724	9536:9550	NA	16773-15229	15095-12348	15095-12348	15095-12348	NA	FALSE		
rhopalosiphum_maidis_NC_040879.1:72475153-72496418	3238:3834	12434:13030	3234:3237/13031:13034	12415:12429	NA	5185-7488	7569-9608	5185-7488	7569-9608	NA	FALSE		
rhopalosiphum_maidis_NC_040879.1:75205173-75226948	NA	NA	NA	NA	NA	4949-5932	7592-8974	6912-7571, 7592-8974	7592-8974, 8943-9914	NA	FALSE		
rhopalosiphum_maidis_NC_040879.1:8453848-8475641	2715:3302	12397:12972	2711:2714/12973:12976	12378:12392	NA	NA	5224-9153	5224-9153, 12989-13384, 17356-17868	5224-9153	NA	FALSE		
rhopalosiphum_maidis_NC_040880.1:38535855-38557477	2331:2948	12060:12679	2327:2330/12680:12683	12041:12055	NA	3111-8819	3111-8819	3111-8819	3111-8819, 17631-18194	NA	FALSE		
rhopalosiphum_maidis_NC_040880.1:45629677-45651611	NA	NA	NA	NA	NA	NA	7637-8320	7637-8320	NA	NA	FALSE		
rhopalosiphum_maidis_NC_040880.1:47121106-47143058	3234:3830	12439:13035	3230:3233/13036:13039	12420:12434	NA	5181-9605	5181-9605	5181-9605	5181-9605	NA	FALSE		
solenopsis_invicta_NC_052664.1:10493380-10515158	NA	NA	NA	NA	NA	5901-9986	5901-9986	5901-9986	5901-9986	NA	FALSE		
solenopsis_invicta_NC_052664.1:10504963-10526741	NA	NA	NA	NA	NA	5901-9986	5901-9986	5901-9986	5901-9986	NA	FALSE		
solenopsis_invicta_NC_052664.1:1941242-1963014	NA	NA	NA	NA	NA	5027-9949	5027-9949	5027-9949	5027-9949	5027-9949	FALSE		
solenopsis_invicta_NC_052664.1:3011431-3033056	NA	NA	NA	NA	NA	16721-11820	16721-11820	16721-11820	64-897, 16721-11820, 6694-6143	64-897, 16721-11820, 6694-6143	FALSE		
solenopsis_invicta_NC_052664.1:4277734-4299506	NA	NA	NA	NA	NA	16775-16086	14978-13899	16123-14879, 14978-13899	20711-21391, 13039-11834	13039-11834	FALSE		
solenopsis_invicta_NC_052665.1:102260-123849	NA	NA	NA	NA	NA	NA	15244-12548	15244-12548	15244-12548	12544-11648	FALSE		
solenopsis_invicta_NC_052665.1:22742704-22764479	NA	NA	NA	NA	NA	NA	4847-9943	4847-9943	4847-9943	4847-9943	FALSE		
solenopsis_invicta_NC_052665.1:23572027-23593799	NA	NA	NA	NA	NA	16740-14275	13993-11825	16740-14275	13993-11825	1220-1939, 13993-11825	FALSE		
solenopsis_invicta_NC_052665.1:24630886-24652631	NA	NA	NA	NA	NA	NA	6496-9918	6496-9918, 19234-18887	32-1603, 6496-9918	6496-9918	FALSE		
solenopsis_invicta_NC_052666.1:10903523-10925295	NA	NA	NA	NA	NA	16741-11825	16741-11825	16741-11825	16741-11825	16741-11825	FALSE		
solenopsis_invicta_NC_052666.1:11014619-11036394	NA	NA	NA	NA	NA	NA	16930-11834	16930-11834	16930-11834	16930-11834	FALSE		
solenopsis_invicta_NC_052666.1:12250090-12271862	NA	NA	NA	NA	NA	5035-6228	7376-9949	6354-7370	7376-9949	7376-9949	FALSE		
solenopsis_invicta_NC_052666.1:1814255-1836012	7497:9578	17512:19587	7493:7496/19588:19591	9617:9631	2383-2039, 1773-784	16372-13691	16372-13691	16372-13691	13752-11911	751-398	FALSE		
solenopsis_invicta_NC_052666.1:4003076-4024851	NA	NA	NA	NA	NA	NA	4843-8847	4843-8847	8927-9943	8927-9943	FALSE		
solenopsis_invicta_NC_052666.1:4946471-4968264	NA	NA	NA	NA	NA	15903-12610	15903-12610	15903-12610	15903-12610, 12781-12260	NA	FALSE		
solenopsis_invicta_NC_052666.1:4956544-4978484	8201:9909	18679:20364	8197:8200/20365:20368	9910:9924	NA	16042-15449, 5830-2537	15414-11956, 5830-2537	15414-11956, 5830-2537	15414-11956, 5830-2537, 2708-2187	21736-21074	FALSE		
solenopsis_invicta_NC_052666.1:4970435-4992231	8120:9548	17812:19240	8116:8119/19241:19244	9549:9563	NA	15890-11820, 2151-1558	15890-11820, 1523-3	15890-11820, 1523-3	15890-11820	20029-19217, 8118-7183	FALSE		
solenopsis_invicta_NC_052666.1:8414253-8436055	2394:2907	18435:18943	2389:2393/18944:18948	2908:2922	17490-17188	6947-6378, 5789-4845	14157-13186	15919-14237, 6469-5741, 5789-4845	53-361, 13081-11915, 4627-3326	605-1132	FALSE		
solenopsis_invicta_NC_052667.1:16684564-16706339	NA	NA	NA	NA	NA	NA	4847-9943	4847-9943	4847-9943	4847-9943	FALSE		
solenopsis_invicta_NC_052667.1:18119598-18141373	NA	NA	NA	NA	NA	NA	4847-9943	4847-9943, 21633-20164	4847-9943	4847-9943	FALSE		
solenopsis_invicta_NC_052667.1:7919798-7941534	NA	NA	NA	NA	NA	NA	NA	7252-7713, 7703-8248	8665-9129	9190-9918, 16262-15927	FALSE		
solenopsis_invicta_NC_052668.1:10420919-10442694	NA	NA	NA	NA	NA	4847-9943	4847-9943	4847-9943	4847-9943	4847-9943	FALSE		
solenopsis_invicta_NC_052668.1:28235465-28257240	NA	NA	NA	NA	NA	NA	14374-11834	16930-14441	14374-11834	14374-11834	FALSE		
solenopsis_invicta_NC_052668.1:28482480-28503674	NA	NA	NA	NA	NA	NA	6578-6988, 7691-9412	5621-6511, 6578-6988	7014-7733, 7691-9412	7691-9412	FALSE		

Sequence	LTR5	LTR3	TSR	PPT	pos_gag	pos_protease	pos_rnase	pos_rt	pos_integrase	pos_transposase	complete	TE	Structure
solenopsis_invicta_NC_052668.1:468951-490708	7512:9581	17482:19561	7508:7511/19562:19565	9620:9634	NA	16155-13471	16155-13471	16155-13471	13359-11911	NA	FALSE		
solenopsis_invicta_NC_052668.1:5051407-5073053	2172:3691	5745:7263	2168:2171/7264:7267	5710:5724	NA	NA	NA	NA	NA	NA	FALSE		
solenopsis_invicta_NC_052669.1:16573341-16594969	NA	NA	NA	NA	NA	NA	7229-7615, 7567-8409	5070-7223, 7229-7615	8434-9807	8434-9807	FALSE		
solenopsis_invicta_NC_052669.1:17832911-17854548	NA	NA	NA	NA	NA	NA	NA	5935-7047	8766-9542, 18841-17897	8766-9542, 18841-17897	FALSE		
solenopsis_invicta_NC_052670.1:5310888-5332681	NA	NA	NA	NA	NA	20445-15979	20445-15979	20445-15979	20445-15979	NA	FALSE		
solenopsis_invicta_NC_052671.1:15744482-15766254	1846:3122	8460:9717	1842:1845/9718:9721	8445:8459	3302-4132, 21756-20818	4003-7347, 20986-17777	4003-7347, 20986-17777	4003-7347, 20986-17777	700-1575, 4003-7347, 20986-17777	700-1575, 20986-17777	FALSE		
solenopsis_invicta_NC_052671.1:15796218-15817918	1663:1942	12048:12335	NOTFOUND	12033:12047	NA	NA	5908-8295	5908-8295	8341-9447	1531-725	FALSE		
solenopsis_invicta_NC_052671.1:17507042-17528643	NA	NA	NA	NA	NA	NA	NA	1496-1963	NA	NA	FALSE		
solenopsis_invicta_NC_052671.1:19301788-19323575	NA	NA	NA	NA	NA	NA	15931-11915	15931-11915	15931-11915	NA	FALSE		
solenopsis_invicta_NC_052671.1:19311917-19333704	NA	NA	NA	NA	NA	NA	15931-11915	15931-11915	15931-11915	NA	FALSE		
solenopsis_invicta_NC_052671.1:19916090-19937280	NA	NA	NA	NA	15808-18657	15808-18657, 18766-20988	NA	15808-18657	18766-20988	NA	FALSE		
solenopsis_invicta_NC_052671.1:24187127-24208188	NA	NA	NA	NA	21061-19934	19636-16586	4847-9943, 19636-16586	4847-9943, 19636-16586	4847-9943, 19636-16586	4847-9943, 19636-16586	FALSE		
solenopsis_invicta_NC_052671.1:24306723-24328336	NA	NA	NA	NA	NA	NA	5728-7704	3477-5768, 5728-7704	5728-7704	7704-8576, 16061-14955	FALSE		
solenopsis_invicta_NC_052671.1:25733565-25755340	NA	NA	NA	NA	NA	4846-6219	6347-9943	6347-9943	6347-9943	6347-9943	FALSE		
solenopsis_invicta_NC_052673.1:164394-186169	NA	NA	NA	NA	NA	NA	4847-9943	4847-9943	4847-9943	4847-9943	FALSE		
solenopsis_invicta_NC_052673.1:5120268-5142040	NA	NA	NA	NA	NA	NA	14953-11825	14953-11825	14953-11825	14953-11825	FALSE		
solenopsis_invicta_NC_052673.1:5628922-5650694	NA	NA	NA	NA	NA	NA	16549-12122	16549-12122	16549-12122	16549-12122	FALSE		
solenopsis_invicta_NC_052674.1:12037214-12058989	NA	NA	NA	NA	NA	NA	16930-11834	16930-11834	16930-11834	16930-11834	FALSE		
solenopsis_invicta_NC_052674.1:12073072-12094841	NA	NA	NA	NA	NA	NA	NA	NA	12770-12048	19636-19938, 12770-12048	FALSE		
solenopsis_invicta_NC_052674.1:13254534-13276321	2085:4291	12218:14425	2081:2084/14426:14429	12166:12180	NA	NA	5822-9874	5822-9874	5822-9874	20936-21469	FALSE		
solenopsis_invicta_NC_052674.1:15398921-15419967	NA	NA	NA	NA	NA	NA	6974-8332	5980-7014, 6974-8332	20115-19405	19401-19006	FALSE		
solenopsis_invicta_NC_052675.1:1081577-1103349	167:3372	16692:19889	163:166/19890:19893	16629:16643	NA	NA	7097-9949	7097-9949	7097-9949	7097-9949	FALSE		
solenopsis_invicta_NC_052675.1:1106550-1128337	2794:2970	12219:12397	NOTFOUND	12167:12181	NA	NA	6359-9874	6359-9874	6359-9874	16481-18208	FALSE		
solenopsis_invicta_NC_052675.1:4229908-4251476	43:322	4538:4817	38:42/4818:4822	4440:4454	430-783, 873-2159	NA	3462-4487, 14639-14247, 14229-12364	2855-3547, 3462-4487, 15453-14809, 14639-14247	873-2159, 19769-19347, 14229-12364	19769-19347	FALSE		
solenopsis_invicta_NC_052676.1:12336004-12357779	NA	NA	NA	NA	NA	NA	15078-13084	15078-13084, 9727-8126	13126-11834	13126-11834	FALSE		
solenopsis_invicta_NC_052676.1:13106660-13128432	NA	NA	NA	NA	NA	NA	14944-11825	16558-14993, 14944-11825	14944-11825	14944-11825	FALSE		
solenopsis_invicta_NC_052676.1:13179098-13200903	8102:9569	18866:20333	8098:8101/20334:20337	9570:9584	NA	NA	17303-14826	17303-14826, 2130-1	14059-12944, 12778-12362	14059-12944	FALSE		
solenopsis_invicta_NC_052676.1:13249384-13271159	NA	NA	NA	NA	NA	NA	6804-8744	6804-8744, 16431-17129, 17006-17626	1079-2161, 8726-9400	NA	FALSE		
solenopsis_invicta_NC_052676.1:15819420-15841207	NA	NA	NA	NA	NA	NA	NA	NA	8997-10001	NA	FALSE		
solenopsis_invicta_NC_052676.1:9925954-9947693	NA	NA	NA	NA	NA	16740-16159	14804-12990	14804-12990	19109-17997, 12850-11834, 1045-737	19109-17997, 12850-11834	FALSE		

Sequence	LTR5	LTR3	TSR	PPT	pos_gag	pos_protease	pos_rnase	pos_rt	pos_integrase	pos_transposase	complete	TE	Structure
solenopsis_invicta_NC_052677.1:2337112-2358884	NA	NA	NA	NA	NA	5031-5948	6062-9949	6062-9949	6062-9949	6062-9949	FALSE		
solenopsis_invicta_NC_052677.1:389378-411150	NA	NA	NA	NA	NA	5033-5650	7031-9949	6066-7043, 7031-9949	7031-9949, 17470-16418	7031-9949	FALSE		
solenopsis_invicta_NC_052677.1:3960402-3982177	NA	NA	NA	NA	NA	NA	7170-8702	NA	8736-9794	8736-9794, 4773-3361	FALSE		
solenopsis_invicta_NC_052677.1:7471997-7493556	7125:9336	17226:19433	7121:7124/19434:19437	9374:9388	NA	19636-21558	14386-11672	19636-21558, 15686-14382, 14386-11672	14386-11672, 7014-6145	NA	FALSE		
solenopsis_invicta_NC_052677.1:7676175-7697932	7497:9578	17492:19565	7493:7496/19566:19569	9617:9631	NA	16165-13691	16165-13691	16165-13691	13752-11911	NA	FALSE		
solenopsis_invicta_NC_052677.1:88042-109811	NA	NA	NA	NA	NA	NA	15421-11822	15421-11822	15421-11822	15421-11822	FALSE		
solenopsis_invicta_NW_024105255.1:31777-53384	NA	NA	NA	NA	NA	5315-5740, 16554-16231	NA	15365-14703, 14578-14180	2958-1846	12328-11669, 2958-1846	FALSE		
solenopsis_invicta_NW_024105270.1:1675-23465	NA	NA	NA	NA	NA	21364-16916	21364-16916	21364-16916	21364-16916	21364-16916	FALSE		
solenopsis_invicta_NW_024105270.1:26630-48420	NA	NA	NA	NA	NA	21359-19926	19905-19096, 19077-18646	19905-19096	18640-17558	NA	FALSE		
solenopsis_invicta_NW_024105278.1:1-13651	NA	NA	NA	NA	NA	NA	6490-4991	6490-4991	4953-3778	4953-3778	FALSE		
solenopsis_invicta_NW_024105292.1:28425-46047	NA	NA	NA	NA	NA	736-2079	2182-5199	2182-5199	2182-5199	NA	FALSE		
solenopsis_invicta_NW_024105314.1:1-14859	NA	NA	NA	NA	9173-10528	10835-14443	7861-4859	10835-14443, 7861-4859	10835-14443, 7861-4859	10835-14443	FALSE		
trachymyrmex_cornetzi_NW_017295680.1:14241-35668	NA	NA	NA	NA	NA	16738-18108	NA	18531-18929, 7872-6370	NA	NA	FALSE		
trachymyrmex_cornetzi_NW_017295770.1:1-10360	NA	NA	NA	NA	NA	NA	8541-8164	10209-8866, 8860-8279	7369-6782	6740-6105	FALSE		
trachymyrmex_cornetzi_NW_017295795.1:252432-274441	NA	NA	NA	NA	NA	NA	18074-17538, 14531-12063	18814-18404	14531-12063	14531-12063	FALSE		
trachymyrmex_cornetzi_NW_017296401.1:32795-54645	NA	NA	NA	NA	NA	NA	17281-16421	NA	NA	15841-13301	FALSE		
trachymyrmex_cornetzi_NW_017296727.1:21634-43610	NA	NA	NA	NA	NA	5870-7060, 17847-21977	NA	5870-7060	13462-13001	NA	FALSE		
trachymyrmex_cornetzi_NW_017296752.1:149407-171161	NA	NA	NA	NA	NA	NA	NA	356-3	NA	NA	FALSE		
trachymyrmex_cornetzi_NW_017296913.1:1-18721	NA	NA	NA	NA	NA	NA	14203-11561	14203-11561	11237-10569	10636-9890	FALSE		
trachymyrmex_cornetzi_NW_017296947.1:1-13602	NA	NA	NA	NA	NA	NA	6576-5911, 5731-4253	7152-6640, 6576-5911	5731-4253	4242-3640	FALSE		
trachymyrmex_cornetzi_NW_017297008.1:7680-29368	NA	NA	NA	NA	NA	21368-21012	14954-14055, 13861-12920	7922-8380, 14954-14055	12784-12281, 362-3	NA	FALSE		
trachymyrmex_cornetzi_NW_017298523.1:40052-61338	NA	NA	NA	NA	NA	NA	14297-13476	14946-14428	NA	13215-12355	FALSE		
trachymyrmex_cornetzi_NW_017298683.1:63360-84628	NA	NA	NA	NA	NA	NA	NA	NA	20459-20968	20459-20968	FALSE		
trachymyrmex_cornetzi_NW_017299818.1:65641-83881	NA	NA	NA	NA	NA	NA	6070-9912	6070-9912	6070-9912	6070-9912	FALSE		
trachymyrmex_cornetzi_NW_017299849.1:2372-23547	NA	NA	NA	NA	NA	NA	NA	NA	16514-17068	NA	FALSE		
trachymyrmex_cornetzi_NW_017299954.1:30849-48919	NA	NA	NA	NA	NA	NA	17623-16385, 6261-5911	17623-16385	15666-15166, 5921-5418	15666-15166, 5410-4748	FALSE		
trachymyrmex_cornetzi_NW_017299990.1:2620-24572	NA	NA	NA	NA	NA	16344-15679	15194-12522	15691-15146, 15194-12522	15194-12522	NA	FALSE		
trachymyrmex_cornetzi_NW_017300084.1:65556-87310	NA	NA	NA	NA	NA	NA	NA	NA	15124-11792	15124-11792	FALSE		
trachymyrmex_cornetzi_NW_017300137.1:32569-54530	8524:9857	17744:19077	8520:8523/19078:19081	9858:9872	NA	NA	14483-13293	15156-14401	13165-12734	12788-12069	FALSE		
trachymyrmex_cornetzi_NW_017300267.1:694591-716489	5834:6338	12697:13190	5829:5833/13191:13195	6347:6361	NA	NA	NA	NA	NA	NA	FALSE		
trachymyrmex_cornetzi_NW_017300318.1:80131-101582	NA	NA	NA	NA	NA	17675-17244	14484-13561	NA	13192-12728	12764-12024	FALSE		
trachymyrmex_cornetzi_NW_017300325.1:3967-25235	NA	NA	NA	NA	NA	NA	14195-13284	15097-14225	NA	12377-11493	FALSE		
trachymyrmex_septentrionalis_NW_017304482.1:1032631-1053950	NA	NA	NA	NA	NA	16455-11392	16455-11392	16455-11392	16455-11392	16455-11392	FALSE		
trachymyrmex_septentrionalis_NW_017304522.1:85803-107308	NA	NA	NA	NA	NA	NA	NA	7662-7315	5982-7118, 18573-19025	5982-7118	FALSE		
trachymyrmex_septentrionalis_NW_017305061.1:36176-57645	NA	NA	NA	NA	NA	16605-11542	16605-11542	5795-6103, 16605-11542	16605-11542	16605-11542	FALSE		
trachymyrmex_septentrionalis_NW_017305485.1:1-15843	NA	NA	NA	NA	NA	NA	247-2067	247-2067	2067-4046	2067-4046	FALSE		

Sequence	LTR5	LTR3	TSR	PPT	pos_gag	pos_protease	pos_rnase	pos_rt	pos_integrase	pos_transposase	complete	TE	Structure
trachymyrmex_septentrionalis_NW_017305562.1:1-17599	NA	NA	NA	NA	NA	NA	3125-4240	2508-3125, 3125-4240	4599-5936	4599-5936	FALSE		
trachymyrmex_zeteki_NW_017255882.1:1-2980	NA	NA	NA	NA	NA	NA	NA	NA	93-1151	93-1151	FALSE		
trachymyrmex_zeteki_NW_017255942.1:1-13287	NA	NA	NA	NA	NA	10093-10500	NA	NA	145-1290	NA	FALSE		
trachymyrmex_zeteki_NW_017255991.1:1-16608	NA	NA	NA	NA	NA	NA	5249-6256	4980-5327, 5249-6256	6479-7414, 16581-15928	7357-7842, 16581-15928	FALSE		
trachymyrmex_zeteki_NW_017257259.1:27709-45645	NA	NA	NA	NA	NA	14961-11014	14961-11014	14961-11014	14961-11014	NA	FALSE		
trachymyrmex_zeteki_NW_017257447.1:26906-47880	NA	NA	NA	NA	NA	NA	13178-12459	14539-13928	12474-11995	20528-20088	FALSE		
trachymyrmex_zeteki_NW_017257537.1:1196-14752	NA	NA	NA	NA	NA	NA	4836-7079	4836-7079	7197-8864	7197-8864	FALSE		
trachymyrmex_zeteki_NW_017257537.1:71-14752	NA	NA	NA	NA	NA	NA	5961-8204	5961-8204	8322-9989	8322-9989	FALSE		
trachymyrmex_zeteki_NW_017257566.1:49152-66061	NA	NA	NA	NA	NA	NA	16832-14862, 14862-13690	14862-13690	14862-13690	879-1802	FALSE		
trachymyrmex_zeteki_NW_017257583.1:18326-32821	NA	NA	NA	NA	NA	1132-536	14456-14055	14456-14055	13987-12632	NA	FALSE		
trachymyrmex_zeteki_NW_017257772.1:2604-24367	NA	NA	NA	NA	NA	7419-6679	14440-13757	14871-14332	13761-12169	13761-12169	FALSE		
trachymyrmex_zeteki_NW_017257775.1:48631-65369	NA	NA	NA	NA	NA	NA	NA	NA	3705-4460, 14623-14928	9300-9998	FALSE		
tribolium_castaneum_NW_015451025.1:1-16782	5894:6132, 8364:8567	11409:11647, 11192:11395	5890:5893/11648:11651, 8360:8363/11396:11399	6142:6156, 8571:8585	NA	10077-9559	228-707	NA	NA	759-1646	FALSE		
tribolium_castaneum_NW_015451657.1:19368-40111	NA	NA	NA	NA	NA	2088-2903	NA	8899-10023, 10010-10354	NA	NA	FALSE		
trichogramma_brassicae_CADCXV010000013.1:1-10527	NA	NA	NA	NA	NA	NA	NA	1731-2174	NA	NA	FALSE		
trichogramma_brassicae_CADCXV010000052.1:1-16865	NA	NA	NA	NA	NA	NA	76-1044, 1005-1385	76-1044	2327-3604	2327-3604	FALSE		
trichogramma_brassicae_CADCXV010000052.1:1-17258	NA	NA	NA	NA	NA	NA	76-1044, 1005-1385	76-1044	2327-3604	2327-3604	FALSE		
trichogramma_brassicae_CADCXV010000331.1:1-16576	NA	NA	NA	NA	NA	NA	NA	477-803	NA	NA	FALSE		
trichogramma_brassicae_CADCXV010000358.1:1-11582	NA	NA	NA	NA	NA	NA	5544-5053	6647-5985, 6061-5612	NA	NA	FALSE		
trichogramma_brassicae_CADCXV010000358.1:369305-390255	NA	NA	NA	NA	20414-18501	NA	17183-15483	17183-15483	15370-14036	15370-14036	FALSE		
trichogramma_brassicae_CADCXV010000691.1:1-15425	NA	NA	NA	NA	NA	NA	184-984	NA	984-1889	984-1889	FALSE		
trichogramma_brassicae_CADCXV010000792.1:22416-43924	NA	NA	NA	NA	NA	NA	15343-16449	15343-16449	NA	NA	FALSE		
trichogramma_brassicae_CADCXV010000792.1:32662-54611	1487:1936	12855:13305	1483:1486/13306:13309	12840:12854	NA	NA	5097-6203, 15900-14866	5097-6203, 16665-15955	14217-13147	NA	FALSE		
trichogramma_brassicae_CADCXV010000929.1:28961-49731	NA	NA	NA	NA	NA	17687-11949	17687-11949	17687-11949	17687-11949	17687-11949	FALSE		
trichogramma_brassicae_CADCXV010000945.1:13940-34461	NA	NA	NA	NA	NA	NA	1517-3994, 4088-5374	1517-3994	4088-5374, 20517-19393	NA	FALSE		
trichogramma_brassicae_CADCXV010000985.1:8840-30348	566:1521	13848:14800	562:565/14801:14804	13833:13847	NA	NA	NA	5497-6099	7482-8042	NA	FALSE		
trichogramma_brassicae_CADCXV010001009.1:26117-42915	NA	NA	NA	NA	NA	1451-1990	4003-4452	2110-3675, 14871-15224	4853-5479	NA	FALSE		
trichogramma_brassicae_CADCXV010001107.1:46316-67155	NA	NA	NA	NA	NA	2452-3066, 13676-14677	4162-5361	4162-5361	5616-6776	NA	FALSE		
trichogramma_brassicae_CADCXV010001107.1:66596-87318	NA	NA	NA	NA	NA	NA	17493-16702, 16598-15888	17493-16702	15399-14788	NA	FALSE		
trichogramma_brassicae_CADCXV010001198.1:50734-72242	NA	NA	NA	NA	NA	1408-1938	2023-3732	2023-3732	3644-5143	NA	FALSE		
trichogramma_brassicae_CADCXV010001206.1:1-17307	NA	NA	NA	NA	NA	NA	11884-11450	12301-11966, 11884-11450	10856-9657	10856-9657	FALSE		
trichogramma_brassicae_CADCXV010001282.1:4977-19834	NA	NA	NA	NA	NA	NA	NA	NA	2014-2850	NA	FALSE		
trichomalopsis_sarcophagae_NNAY01006280.1:1-9122	NA	NA	NA	NA	NA	3159-4460	NA	NA	NA	4592-5488	FALSE		
trichomalopsis_sarcophagae_NNAY01027560.1:1-3860	NA	NA	NA	NA	NA	NA	NA	NA	218-1897	218-1897	FALSE		
vespa_mandarinia_NW_023395894.1:105455-127017	7433:8145, 7776:8145, 8118:9976	19680:20375, 19680:20034, 17818:19676	7429:7432/20376:20379, 7772:7775/20035:	8195:8209, 17734:17748	NA	15903-12031	15903-12031	15903-12031	15903-12031	15903-12031	FALSE		

Sequence	LTR5	LTR3	TSR	PPT	pos_gag	pos_protease	pos_rnase	pos_rt	pos_integrase	pos_transposase	complete	TE	Structure
			20038, 8114:8117/19677:19680										
vespa_mandarinia_NW_023395894.1:16858-38420	41:203, 1975:3746	13817:13979, 11588:13359	37:40/13980:13983, 1971:1974/13360:13363	13718:13732, 11573:11587	NA	5661-9533	5661-9533	5661-9533	5661-9533	5661-9533	FALSE		
vespa_mandarinia_NW_023395894.1:225301-246863	NA	NA	NA	NA	NA	5661-9533	5661-9533	5661-9533	5661-9533	5661-9533	FALSE		
vespa_mandarinia_NW_023395894.1:22755-44317	7478:7873, 8118:9976	21086:21483, 17818:19676	7474:7477/21484:21487, 8114:8117/19677:19680	21023:21037, 17734:17748	NA	15903-12031	15903-12031	15903-12031	15903-12031	15903-12031	FALSE		
vespa_mandarinia_NW_023395894.1:47865-69427	8112:9976	17818:19676	8108:8111/19677:19680	17734:17748	NA	15903-12031	15903-12031	15903-12031	15903-12031	15903-12031	FALSE		
vespa_mandarinia_NW_023395894.1:60547-82109	8118:9976	17818:19685	8114:8117/19686:19689	17734:17748	NA	15903-12031	15903-12031	15903-12031	15903-12031	15903-12031	FALSE		
vespa_mandarinia_NW_023395894.1:78940-100502	8118:9976	17818:19676	8114:8117/19677:19680	17734:17748	NA	15903-12031	15903-12031	15903-12031	15903-12031	15903-12031	FALSE		
vespa_mandarinia_NW_023395894.1:92775-114337	8118:9976	17818:19676	8114:8117/19677:19680	17734:17748	NA	15903-12031	15903-12031	15903-12031	15903-12031	15903-12031	FALSE		
vespa_mandarinia_NW_023395902.1:16746-38302	860:1877, 1542:1877, 1900:3715	13431:14447, 13431:13765, 11582:13442	855:859/14448:14452, 1537:1541/13766:13770, 1896:1899/13443:13446	13351:13365, 3784:3798	NA	5629-8940	5629-8940	5629-8940	5629-8940	NA	FALSE		
vespa_mandarinia_NW_023395914.1:467797-489359	8114:9976	17818:19680	8110:8113/19681:19684	9977:9991	NA	15903-12556	15903-12556	15903-12556	15903-12556	NA	FALSE		
vespa_mandarinia_NW_023395951.1:18246-39808	8112:9976	17818:19684	8108:8111/19685:19688	17734:17748	NA	15903-12031	15903-12031	15903-12031	15903-12031	15903-12031	FALSE		
vespa_mandarinia_NW_023395951.1:53642-75204	174:1873, 515:1873, 856:1873, 1197:1873, 1538:1873, 1880:3746	13456:15154, 13456:14813, 13456:14473, 13456:14132, 13456:13791, 11588:13452	169:173/15155:15159, 510:514/14814:14818, 851:855/14474:14478, 1192:1196/14133:14137, 1533:1537/13792:13796, 1876:187	13360:13374, 11573:11587	NA	5661-9533	5661-9533	5661-9533	5661-9533	5661-9533	FALSE		

Sequence	LTR5	LTR3	TSR	PPT	pos_gag	pos_protease	pos_rnase	pos_rt	pos_integrase	pos_transposase	complete	TE	Structure
			9/13453:13456										
vespa_mandarinia_NW_023395973.1:170490-192052	34:1875, 1518:1875, 1878:3746	13659:15502, 14341:16183, 14682:16524, 15023:16865, 15704:17548, 16045:17889, 16726:18571, 17067:18912, 17409:19252, 17750:19593, 18091:19933, 18773:20615, 19114:20956, 19454:21297, 13445:13798, 11588:13456	30:33/15503:15506, 30:33/16184:16187, 30:33/16525:16528, 30:33/16866:16869, 30:33/17549:17552, 30:33/17890:17893, 30:33/18572:18575, 30:33/18913:18916, 30:33/19253:19256, 30:33/19594:19597, 30:33/19934:19937, 30:33/20616:20619, 30:33/20957:20960, 30:33/21298:21301, 1513:1517/13799:13803, 1874:1877/13457:13460	13606:13620, 14288:14302, 14629:14643, 14970:14984, 15651:15665, 15992:16006, 16673:16687, 17014:17028, 17356:17370, 17697:17711, 18027:18041, 18720:18734, 19061:19075, 19401:19415, 13365:13379, 11573:11587	NA	5661-9533	5661-9533	5661-9533	5661-9533	5661-9533	FALSE		
vespa_mandarinia_NW_023395985.1:37305-58873	NA	NA	NA	NA	12319-12807	NA	NA	6703-7032, 17400-17999	NA	NA	FALSE		
vespa_mandarinia_NW_023395985.1:51269-72837	NA	NA	NA	NA	9251-8763	NA	NA	3436-4035, 14867-14538	NA	NA	FALSE		
vespa_mandarinia_NW_023396000.1:53508-75070	2634:4492	11588:13446	2630:2633/13447:13450	11573:11587	NA	6407-7426	7410-9533	6407-7426	7410-9533	7410-9533	FALSE		

Sequence	LTR5	LTR3	TSR	PPT	pos_gag	pos_protease	pos_rnase	pos_rt	pos_integrase	pos_transposase	complete	TE	Structure
vespa_mandarinia_NW_023396008.1:60170-81732	17:661, 17:320, 17:1002, 699:3388, 1040:3388, 1722:3388, 2063:3388, 2404:3388, 2745:3388	2745:3388, 3085:3388, 2404:3388, 5291:7980, 5291:7639, 5291:6958, 19709:21376, 5291:6617, 19709:21035, 5291:6276, 19709:20694, 5291:5935, 19709:20353	12:16/3389:3393, 694:698/7981:7985, 1035:1039/7640:7644, 1717:1721/6959:6963, 1717:1721/21377:21381, 2058:2062/6618:6622, 2058:2062/21036:21040, 2399:2403/6277:6281, 2399:2403/20695:20699, 2740:2744/5936:5940, 2740:2744/20354:20358	2720:2734, 357:371, 2379:2393, 5266:5280, 19684:19698	NA	15903-12031	15903-12031	15903-12031	15903-12031	15903-12031	FALSE		
vespa_mandarinia_NW_023396008.1:74594-96156	NA	NA	NA	NA	NA	15903-12031	15903-12031	15903-12031	15903-12031	15903-12031	FALSE		
vespa_mandarinia_NW_023396008.1:89018-110580	8110:9976	17818:19684	8106:8109/19685:19688	17734:17748	NA	15903-12031	15903-12031	15903-12031	15903-12031	15903-12031	FALSE		
vespa_mandarinia_NW_023396016.1:67240-88808	NA	NA	NA	NA	9251-8763	NA	NA	14897-14319	NA	NA	FALSE		
vespa_mandarinia_NW_023396039.1:37914-59476	55:2824, 396:3165, 733:3506, 5412:6134, 5412:5799, 5435:6127, 8157:9976	5412:8184, 7441:8184, 7782:8184, 19629:20325, 17818:19633	50:54/8185:8189, 391:395/8185:8189, 728:732/8185:8189, 5407:5411/8185:8189, 5430:5434/20326:20330, 8153:8156/19634:19637	5342:5356, 7386:7400, 7729:7743, 6155:6169, 17734:17748	NA	15903-12031	15903-12031	15903-12031	15903-12031	15903-12031	FALSE		
vespa_mandarinia_NW_023396044.1:27658-49220	41:179, 41:343, 41:684, 1890:3746	1744:1883, 14325:14628, 13984:14628, 11588:13444	37:40/1884:1887, 36:40/14629:14633, 1886:188	218:232, 14283:14297, 13935:13949, 11573:11587	NA	5661-9533	5661-9533	5661-9533	5661-9533	5661-9533	FALSE		

Sequence	LTR5	LTR3	TSR	PPT	pos_gag	pos_protease	pos_rnase	pos_rt	pos_integrase	pos_transposase	complete	TE	Structure
			9/13445:13448										
vespa_mandarinia_NW_023396044.1:40423-61985	879:1863, 1219:1863, 1889:3746	13462:14446, 13462:14105, 11588:13445	874:878/14447:14451, 1214:1218/14106:14110, 1885:1888/13446:13449	1874:1888, 11573:11587	NA	5661-9533	5661-9533	5661-9533	5661-9533	5661-9533	FALSE		
vespa_mandarinia_NW_023396044.1:54003-75565	1889:3746, 13488:14131	11588:13446, 15192:15836	1885:1888/13447:13450, 13483:13487/15837:15841	11573:11587, 15150:15164	NA	5661-9533	5661-9533	5661-9533	5661-9533	5661-9533	FALSE		
vespa_mandarinia_NW_023396044.1:61731-83293	5760:6403, 5760:7603, 5760:6921, 6782:8108, 7464:8108, 8119:9976	7464:8108, 19708:21553, 20390:21553, 19708:21034, 19708:20352, 17818:19676	5755:5759/8109:8113, 5756:5759/21554:21557, 6777:6781/21035:21039, 7459:7463/20353:20357, 8115:8118/19677:19680	7422:7436, 7642:7656, 6960:6974, 19666:19680, 17734:17748	NA	15903-12031	15903-12031	15903-12031	15903-12031	15903-12031	FALSE		
