# Supplementary material for: Ancient origin of Jingchuvirales derived glycoproteins integrated in arthropod genomes
Source: Genet Mol Biol. 2023 Apr 7;46(1):e20220218. doi: 10.1590/1678-4685-GMB-2022-0218 (PMC10084718; doi:10.1590/1678-4685-GMB-2022-0218)
Supplement: Figure S1 - [file 1415-4757-GMB-46-1-e20220218-s6.pdf]

*Genetics and Molecular Biology*
https://doi.org/10.1590/1678-4685-GMB-2022-0218

# Supplementary Material to "Ancient origin of Jingchuvirales derived glycoproteins integrated in arthropod genomes"

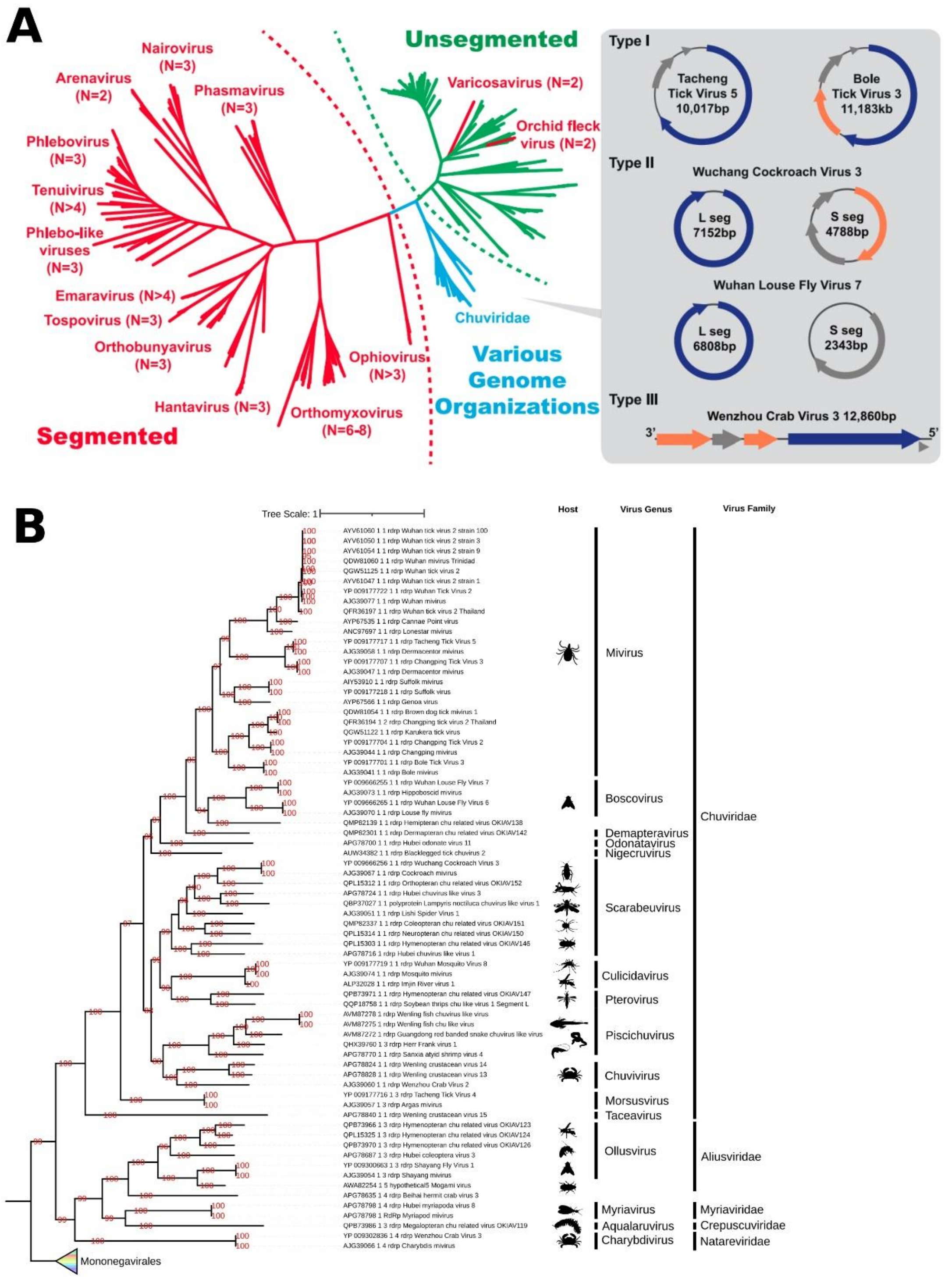


**Figure S1** - Jingchuvirales phylogeny from different studies. A - Figure 4 from Ci-Xiu Li, Mang Shi, Jun-Hua Tian, Xian-Dan Lin, Yan-Jun Kang, Liang-Jun Chen, Xin-Cheng Qin, Jianguo Xu, Edward C Holmes, Yong-Zhen Zhang (2015). Unprecedented genomic diversity of RNA viruses in arthropods reveals the ancestry of negative-sense RNA viruses eLife 4:e05378https://doi.org/10.7554/eLife.05378. B - Schema from Figure 2, present study.
